# Heterozygous Mapping Strategy (HetMappS) for High Resolution Genotyping-By-Sequencing Markers: A Case Study in Grapevine

**DOI:** 10.1371/journal.pone.0134880

**Published:** 2015-08-05

**Authors:** Katie E. Hyma, Paola Barba, Minghui Wang, Jason P. Londo, Charlotte B. Acharya, Sharon E. Mitchell, Qi Sun, Bruce Reisch, Lance Cadle-Davidson

**Affiliations:** 1 Bioinformatics Facility, Institute of Biotechnology, Cornell University, Ithaca, New York, United States of America; 2 Genomic Diversity Facility, Institute of Biotechnology, Cornell University, Ithaca, New York, United States of America; 3 Plant Breeding and Genetics Section, School of Integrative Plant Science, Cornell University, Ithaca, New York, United States of America; 4 USDA-ARS Grape Genetics Research Unit, Geneva, New York, United States of America; 5 Horticulture Section, School of Integrative Plant Science, Cornell University, Geneva, New York, United States of America; Agriculture and Agri-Food Canada, CANADA

## Abstract

Genotyping by sequencing (GBS) provides opportunities to generate high-resolution genetic maps at a low genotyping cost, but for highly heterozygous species, missing data and heterozygote undercalling complicate the creation of GBS genetic maps. To overcome these issues, we developed a publicly available, modular approach called HetMappS, which functions independently of parental genotypes and corrects for genotyping errors associated with heterozygosity. For linkage group formation, HetMappS includes both a reference-guided synteny pipeline and a reference-independent *de novo* pipeline. The *de novo* pipeline can be utilized for under-characterized or high diversity families that lack an appropriate reference. We applied both HetMappS pipelines in five half-sib F_1_ families involving genetically diverse *Vitis spp*. Starting with at least 116,466 putative SNPs per family, the HetMappS pipelines identified 10,440 to 17,267 phased pseudo-testcross (Pt) markers and generated high-confidence maps. Pt marker density exceeded crossover resolution in all cases; up to 5,560 non-redundant markers were used to generate parental maps ranging from 1,047 cM to 1,696 cM. The number of markers used was strongly correlated with family size in both *de novo* and synteny maps (r = 0.92 and 0.91, respectively). Comparisons between allele and tag frequencies suggested that many markers were in tandem repeats and mapped as single loci, while markers in regions of more than two repeats were removed during map curation. Both pipelines generated similar genetic maps, and genetic order was strongly correlated with the reference genome physical order in all cases. Independently created genetic maps from shared parents exhibited nearly identical results. Flower sex was mapped in three families and correctly localized to the known sex locus in all cases. The HetMappS pipeline could have wide application for genetic mapping in highly heterozygous species, and its modularity provides opportunities to adapt portions of the pipeline to other family types, genotyping technologies or applications.

## Introduction

High throughput sequencing provides opportunities for generating high-resolution genetic maps at a low per-sample genotyping cost. While whole genome sequencing is currently a feasible and cost-effective approach to genotyping organisms with smaller genomes, the genotyping of larger genomes benefit from reduced representation library (RRL) approaches. The low cost and feasibility make this an ideal marker system for use in both model and non-model organisms, including specialty crops like grapevine.

While reduced representation approaches such as genotyping-by-sequencing (GBS) (reviewed in [1]) have been applied successfully for genetic map creation in inbred lines [2–4], organisms with highly heterozygous genomes, particularly high-diversity species, present additional computational challenges. The primary challenge for analysis of GBS data from heterozygous species stems from its primary advantage: GBS is a highly multiplexed, shallow sequencing strategy designed to simplify library production and minimize per sample cost. Shallow sequencing coverage results in missing data, genotyping error, and under-calling of heterozygous sites. While imputation of missing genotypes is practical in homozygous samples with known haplotypes and sequence order, imputation is error-prone for heterozygous samples of diverse materials or those lacking a suitable reference genome to infer order [5, 6].

Traditionally, genetic map construction in heterozygous crosses has utilized low-resolution markers with a low amount of missing data, infrequent genotyping error, and high quality parental genotype data [7]. In contrast, GBS queries thousands of markers with a high proportion of missing data and variable genotyping error, and some datasets lack parental genotypes. Recent genotyping methods have been developed to overcome the problems of low sequence coverage and the lack of parental genotypes [8], but are optimized for inbred samples and not for heterozygous, outcrossing species. GBS data generated from crosses between two heterozygous parents are further complicated by difficulty in linkage group (LG) formation and phasing of markers, especially when high quality genotypes of the parents is not available. Methods are available for LG formation and phasing, but most are either computationally infeasible for large data sets [9], and thus require filtering SNPs prior to analysis [10, 11], or have high error rates associated with low coverage, missing data and genotype error [12, 13]. Other software provide tools for LG formation but require markers to be previously phased [14]. Methods for haplotype discovery in diverse non-pedigree related populations can be applied, but many of these methods are computationally intractable for large datasets, or are strongly affected by genotyping error [15].

Grapevine exemplifies the high genetic diversity and heterozygosity of many specialty crops. Most specialty crop geneticists use low-throughput marker platforms, of which simple sequence repeats (SSRs) are most common. SSRs have the advantage of being transferable across diverse germplasm [16, 17] and can provide more polymorphisms per marker than biallelic SNPs [18]. However, logistical limits on multiplexing in data collection and analysis make SSRs an expensive marker technology for genetic mapping, primarily due to time involved and labor costs. As a result, SSR maps are typically low resolution, ranging from 100 to 600 markers per genome [19–22]. Linkage disequilibrium decay in grapevines is rapid, with multilocus r^2^ values declining down to 0.1 within 2.7 cM when measured as r^2^ between SSRs [23] and faster than humans, *Arabidopsis* and maize when measured as r^2^ between SNPs [24]. In marker assisted selection it is desirable to maintain linkage between marker and QTLs in a diverse genetic background, hence denser genetic maps are needed. SNP genotyping microarrays provided an improved option for higher resolution maps with 1,000 markers or more and low labor cost, but high technology cost. However, genotyping via microarrays suffers from ascertainment bias, and in high diversity species like grapevine, markers useful for one family are often not transferable even to closely related families because of flanking unknown SNPs [25, 26]. Recently, NGS has been successfully applied to marker discovery and genetic mapping in grapevines, but these SNP sets have been filtered down to meet the limits of the genetic mapping software used [10, 27, 28].

Hermaphroditism is a predominant domestication-related trait that gave rise to cultivated *Vitis vinifera* [29], with wild grapevines being dioecious. Flower sex is due to a single major locus with three alleles controlling male (M), hermaphrodite (H) and female (*f*) flower sex, where M is dominant over H, which is dominant over *f*. Genetic mapping has located the sex locus to chromosome 2, closely linked to the SSR locus VVIB23 [30–33]. Two independent research groups have fine mapped the sex locus between 4.91 and 5.05 Mbp [34] and between 4.89 and 5.04 Mbp [35] of the 12x.0 version of the PN40024 reference genome [36, 37].

In the current study, we developed a modular computational pipeline for constructing and curating genetic maps from grape GBS data, with the option of using synteny to assign markers to chromosomes, or *de novo* assignment to LGs. We selected families expected to be challenging—wide crosses involving several highly diverse *Vitis* spp. The method does not rely on known parental genotypes, but rather on the predictable genetic hallmarks of F_1_ progeny, and this, along with utilizing a measure of genotype quality to mitigate potential heterozygote under-calling and correct putative genotyping errors, allows us to retain a higher number of markers than relying on high quality parental genotypes alone. We present results of the synteny based and *de novo* pipelines and curation for five half-sib families, and demonstrate localization of the flower sex locus on chromosome 2.

## Materials and Methods

### Plant material and phenotype

Five half sib, interspecific F_1_ families were generated (S1 Fig) using the following parental genotypes: *V*. *vinifera* ‘Chardonnay’ (hermaphrodite), *V*. *cinerea* B9 (male), *V*. *rupestris* B38 (female) and the hybrids ‘Horizon’ (‘Seyval’ x ‘Schuyler’, whose pedigree includes *V*. *vinifera*, *V*. *labrusca*, *V*. *aestivalis* and *V*. *rupestris*, hermaphrodite) and Illinois 547–1 (*V*. *rupestris* B38 x *V*. *cinerea* B9, male). The ‘Horizon’ x Illinois 547–1 family resulted from a cross made in 1988 [30], and was enlarged with additional seedlings from a cross made in 1996. Additionally, two crosses were made in 2008: *V*. *rupestris* B38 x ‘Chardonnay’ and *V*. *rupestris* B38 x ‘Horizon’; and two crosses were made in 2009: ‘Horizon’ x *V*. *cinerea* B9 and ‘Chardonnay’ x *V*. *cinerea* B9. Seeds were stratified and germinated the following year, and seedlings were grown in an irrigated vineyard nursery for one season followed by transplantation to a permanent vineyard in Geneva, New York. No specific permissions were required for these plantings on Cornell University’s agricultural land, which did not involve endangered or protected species.

For each progeny vine, flower sex was determined by visual observation during flowering time and fruit set, during two consecutive years. Fruit formation was confirmed later in the season. Male vines (including the parents Illinois 547–1 and *V*. *cinerea* B9) rarely set fruit, whereas hermaphrodite vines (including the parents ‘Chardonnay’ and ‘Horizon’) and the female parent *V*. *rupestris* B38 produced berries with fertile seeds.

### Sample collection and DNA extraction

For each vine, a single small leaf (less than 1cm diameter) was harvested and placed in one tube of a Costar 96-well cluster tube collection plate (Corning, Corning NY, USA). Each 96-well plate received up to 91 unique samples plus two sets of duplicated and a blank well to serve as quality controls. The location of the blank well was unique for each plate in order to independently confirm the identity of plate after sequencing. Leaf tissue was maintained at 4°C from harvest until delivery to the laboratory. Upon delivery, two stainless steel genogrinder beads were placed in each tube and the entire plate was frozen at -80°C. When completely frozen, two 96-well plates were agitated at 2x400 speed for 1 minute in a Geno/Grinder 2000 (OPS Diagnostics LLC, Lebanon NJ, USA). Plates were then stored at -80°C until processing with DNeasy 96-well DNA extraction kits (Qiagen, Valencia CA, USA). The following modifications were made from the manufacturer’s protocol to improve DNA quality and quantity: 1) PVP-40 (2% w/v) was added to the AP1 lysis buffer prior to heating of the buffer, and 2) the agitation step following AP1 addition was amended to include visual inspection of each 8-tube strip for complete re-suspension of the tissue pellet by hand or vortex. For the F_1_ family *V*. *rupestris* B38 x ‘Chardonnay’, whole genome amplification was performed using 10 ng of DNA and the Illustra GenomiPhi V2 DNA Amplification Kit (GE Healthcare) to obtain 1.0 μg of dried DNA per sample. Then, 48-plex GBS library preparation and sequencing was completed, as described previously [10]. For the other four F_1_ families, DNA was quantified using the QuantiFlor dsDNA System (Promega) and processed as described below.

### Library preparation

384-plex GBS libraries were prepared using a protocol modified from [38]. *Ape*KI barcode and common adapters (3ng each) were transferred into four 96-well plates, a different barcode adapter per well, and dried in a speed-vac. Sequences and barcodes comprising these four 96-plex adapters are presented in S1 Table. DNA (100ng each) from four 96-well DNA plates described above was transferred to one of the four 96-plex adapter layouts, such that each DNA sample was assigned a unique barcode adapter. DNA digestion, adapter ligation, 96-well sample pooling, sample clean-up and PCR were performed as described in [38] except DNA samples were digested with 1U *Ape*KI, and 18μL pooled template DNA was used in each of four PCRs (one PCR per 96-plex adapter set). The resulting four 96-plex GBS libraries were quantified using a fluorometer (Qubit, Life Technologies, Grand Island NY, USA), diluted to 2 nM and combined in equal volumes for sequencing. Single-end sequences (100bp) were collected on the HiSeq2000 (Illumina Inc., San Diego CA, USA) at the Institute of Biotechnology, Genomics Facility, Cornell University, Ithaca, NY.

### SNP calling

A dataset derived from GBS sequencing of 8,353 *Vitis* spp. samples from the USDA-NIFA Specialty Crops Research Initiative VitisGen project (www.vitisgen.org) was used to call SNPs with the TASSEL-GBS pipeline, version 3.0.139 [39], an extension to the Java program TASSEL [40]. This VitisGen dataset included the four F_1_ families (*V*. *rupestris* B38 x ‘Horizon’ with 215 individuals, ‘Horizon’ x Illinois 547–1 with 366 individuals, *V*. *vinifera* ‘Chardonnay’ x *V*. *cinerea* B9 with 148 individuals and ‘Horizon’ x *V*. *cinerea* B9 with 162 individuals) described above. Sequence data for the F_1_ family *V*. *rupestris* B38 x *V*. *vinifera* ‘Chardonnay’ was previously generated, and SNPs were called independently for this family as described previously [10].

Tags (sorted, trimmed and collapsed de-barcoded sequence reads) were filtered and merged to generate a list of unique sequence tags for the VitisGen dataset. Then, tags were aligned to the 12X.0 *Vitis vinifera* reference genome PN40024 [36, 37] using BWA version 0.6.2-r126 [41], with a maximum edit distance of 0.04. Only tags aligned to unique positions were used by the TASSEL-GBS pipeline during SNP calling. Chromosome names in the SAM file were modified for compatibility with the TASSEL-GBS pipeline in the following manner: Leading “EG:” was removed from chromosome names, trailing “_random” was replaced with “00”, and “Un” was replaced with “999”. The BWA generated SAM file was converted to the Tags on Physical Map (TOPM), and individual Tags-by-Taxa (TBT) files were created for each individual sequencing lane.

SNPs were called from the TOPM and TBT files using the TASSEL-GBS pipeline. Genotype assignment during SNP calling follows [42], where likelihood scores for each possible genotype were calculated according to formula 3.8, and the most likely genotype was assigned. The GQ (genotype quality) score or phred-scaled confidence that the true genotype is the one provided in GT, is calculated according to GATK software [43]. Plugins and values used are described in S2 Table.

Resulting VCF files containing genotype information were compressed with bgzip and indexed with tabix version 0.2.5 (r964) [44]. Separate files for each chromosome were concatenated into a single chromosome with the vcftools (version 0.1.9) utility vcf-concat. Individual datasets for each F_1_ family were extracted from the VitisGen dataset using VCFtools (version 0.1.10) [45]. The VCF files produced by the TASSEL-GBS pipeline differ from the standard format in that the allele order is listed in major/minor order, rather than in reference/alternate order.

### Quality control within F_1_ families

To identify potential sources of contamination such as pollen impurity or mislabeling, each bi-parental family was considered for quality control separately. Genotypes were filtered on genotype quality (GQ ≥ 98), and relatedness was calculated using VCFtools, invoking options—GQ 98 and—relatedness [45]. Relatedness of each progeny against each parent was visualized using R [46]. Progeny that failed to have near zero relatedness to both parents and or failed to cluster with most other progeny were flagged for removal. Mendelian errors were also calculated with the PLINK option—mendel [47]. The proportion of male incompatible Mendelian errors (eg progeny genotypes carrying an allele not present in the male) to female incompatible Mendelian errors (eg progeny genotypes carrying an allele not present in the female) was calculated; progeny that showed elevated paternal to maternal incompatibilities were marked as pollen contaminants (including self-pollination) and flagged for removal. Individuals were removed based on the intersection of the relatedness and Mendelian errors results.

### HetMappS pipeline

**Fig 1:**
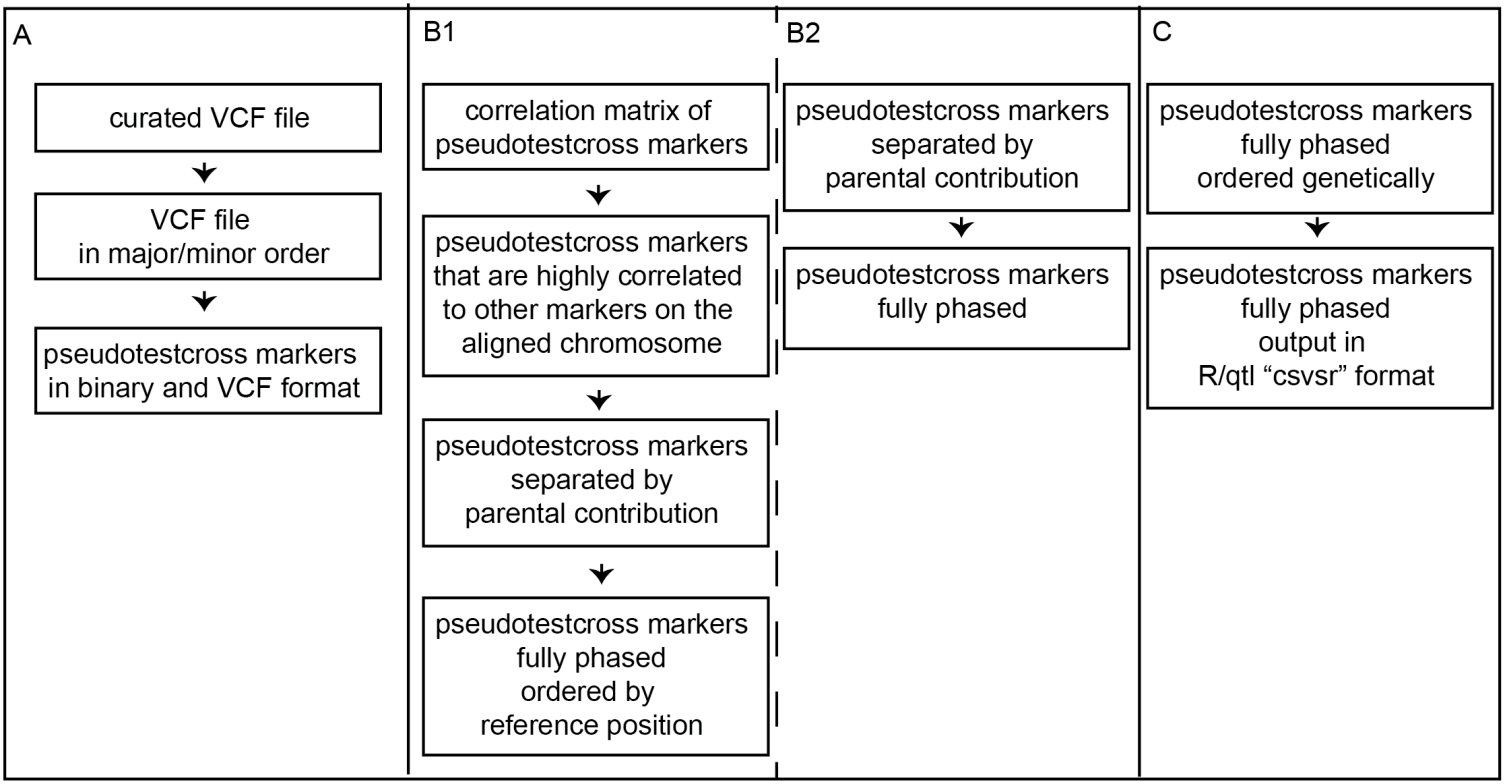
Overview of the HetMappS pipelines. (A) shared initial steps resulting in identification of pseudo-testcross markers, (B) linkage group creation and phasing steps, either (B1) synteny or (B2) *de novo*, and (C) genetic ordering and formatting for R/qtl.

Quality-filtered GBS SNP datasets for each F_1_ family were analyzed independently with the HetMappS pipeline using two approaches (Fig 1). Following (A) pseudo-testcross marker identification, marker grouping and ordering for each family was performed using one of two pipelines: (B1) a synteny pipeline, for which markers were initially separated into chromosomal groups based on alignment to the reference genome, filtered based on linkage, and then phased; or (B2) *de novo* genetic map pipeline, with linkage group (LG) formation based solely on progeny genotypes, regardless of alignment position, followed by phasing within LG. For both pipelines, although optionally for the synteny pipeline, (C) genetic ordering was carried out using MSTMap [48], and resulting maps were exported as cross files for analysis in R/qtl [14]. Documentation for HetMappS, developed for a Linux operating system, can be found in S1 File.

#### Parent-independent identification of pseudo-testcross (Pt) markers

Pseudo-testcross (Pt) markers (markers that are heterozygous in one parent and homozygous in the other, eg, AA x AB [7]) were identified based on segregation patterns within the progeny. Inferring Pt markers based on progeny data eliminated the need for deeply sequenced parental genotypes of high quality and thereby maximized the number of retained markers. When provided, parental and grandparental genotypes with genotype quality (GQ) > 98 were retained for downstream validation.

Genotyping errors, including those associated with low coverage were then removed or corrected in the following manner. First, progeny genotypes were curated to remove alleles with less than 5% allele frequency (putative sequencing errors) and to remove SNPs with a genotyping rate less than 50%. SNPs were selected based on their allele frequencies, genotype frequencies and levels of heterozygosity. SNPs with two segregating alleles (of frequency ≥ 5%), two or more segregating genotypes (of frequency ≥ 5%), minor allele frequencies of 0.25 ± 0.125, and major allele frequencies of 0.75 ± 0.125 were retained. For retained SNPs, genotypes were error corrected as follows: 1) AA genotypes (homozygous for major allele) with GQ < 98 were converted to missing and marked as masked, as they are potentially heterozygous genotypes that have been under sampled, 2) BB genotypes (homozygous minor allele) with GQ ≥ 98 were converted to missing and marked as errors, as this genotype cannot exist for Pt markers, 3) BB genotypes with GQ < 98 were assumed to be under sampled heterozygotes, converted to AB (heterozygous) and marked as corrected. Finally, SNPs were curated again to remove sites with genotyping rate less than 50% or with an error rate greater than 5%, measured as proportion of genotypes BB with GQ ≥ 98 (S2 Fig). The remaining markers are presumed Pt markers. A vcf file and “binary” file (with genotypes encoded as 1 or 0, indicating presence or absence of the minor allele) were generated for progeny and progenitor datasets. Progenitor and progeny genotypes were analyzed independently. All progenitor genotypes with GQ < 98 were filtered, and remaining genotypes at putative Pt loci were output.

#### Synteny option (reference-guided approach)

For the HetMappS Synteny Pipeline, Pt markers were initially grouped into physical chromosomes based on the coordinates of the reference genome; thus, SNPs identified from alignments with unassembled portions of the reference genome were not considered for further analysis. As described below, for each physical chromosome, a clustering method was used to separate maternal and paternal LGs and to determine phases within each LG.

The genetic linkage of SNPs within each chromosomal group (as defined from physical alignment) was tested to discard mis-assigned markers using an r^2^ matrix based on presence/absence of the minor allele. A filtering parameter (“diff”) was set up as follows: Mean correlation between each query SNP with target SNPs across each of the 19 grapevine chromosomes was calculated and ranked. A query SNP was retained only if the mean correlation with the reference-assigned chromosome was at least two times (diff = 2) higher than the second mean chromosome correlation. All other SNPs were discarded.

Following correlation-based filtering, SNPs were separated into two or more LGs per chromosome, corresponding to segregation of the minor alleles, using the R package “WGCNA” [49]. Originally created for analysis of microarray data, this package was used to produce an adjacency matrix with the square of the marker correlations derived from binary genotype data. A topological overlap matrix is calculated from this adjacency matrix using the WGCNA function “TOMdist” and used for hierarchical clustering. For each chromosome, LGs were created by cutting of the resulting dendrogram into distinct groups of SNPs.

After LG formation, the same procedure was used to phase SNPs within each LG. However, the adjacency matrix was calculated as the marker correlation, with negative correlations converted to 0, as markers within the same phase are expected to be only positively correlated. Potentially mis-assigned markers, correlated with SNPs in both parental groups, were removed using “diff” = 2.

#### *De novo* option (non-reference approach)

The *de novo* pipeline differs from the above in that this approach does not rely on the physical position of the detected SNPs in the *Vitis* reference genome to create LGs. As such, this tool is a valuable addition for researchers studying heterozygous species where the reference genome is under construction, not accurate, or expected to be too diverged for the synteny pipeline.

LGs are formed through the clustering method described for the synteny-based LG formation, but across the entire dataset rather than within each chromosome. In order to determine the combination of cut height and minimum cluster size that results in LGs representing each chromosome for each parental map, the resulting dendrogram was cut for all pairwise combinations of a number of different static heights (0.95, 0.9375, 0.925, 0.9125, 0.9, 0.8875, 0.875, 0.8625 and 0.85,) and minimum cluster sizes (50, 100, 150, 200, 250, and 300). Then, LGs defined for each set of parameters were tested for mis-assigned markers using a correlation-based filtering similar to that described for chromosome assignment, but using LG assignment in lieu of chromosome alignment (here diff = 2, using correlation squared). For each family, a cut height and minimum cluster size of 50 was chosen for which at least 38 LGs were created, corresponding to the 19 chromosomes from each parent, and that maximized the proportion of markers assigned to a LG. After LG formation, the assignment of phases within each LG was perform as described above for the synteny pipeline, with the exception of filtering for mis-assigned markers, which was already performed as described above, during the LG formation step in the *de novo* pipeline.

#### Standardization of genetic maps

LGs were renamed and grouped into parental maps of 19 LGs, according to the grapevine community standards. First, correspondence of LG and its marker’s physical position on the 12X.0 PN40024 reference genome [36, 37] was determined. In the synteny pipeline this information is used to generate LGs, and was incorporated in the pipeline LG name, in a format ‘chr number’_’cluster’, where each chromosome/cluster combination represents a single LG. In the *de novo* pipeline, LGs were named arbitrarily, as the physical position was not considered to generate them. The *de novo* pipeline’s random LG name was replaced by a physical chromosome number (as contained in the input VCF file) according to the chromosome with highest representation in the physical position of the SNPs. Each LG was assigned to the parent in which segregation of the minor allele was observed for the majority of the markers. LGs originating from the female parent were labeled from 1 to 19, and LGs originating from the male parent were labeled from 20 to 38.

Within each LG, phased clusters were renamed when grandparental information was available or when two LGs were joined. In the first case, the origin of each phased cluster can be assigned to a biological grandparent in which segregation of the minor allele was observed for the majority of the markers. Here, for the ‘Horizon’ x Illinois 547–1 family, *V*. *rupestris* B38 and *V*. *cinerea* B9 genotypes were used to validate proper phasing for LGs corresponding to the Illinois 547–1 parental contribution for each chromosome.

In the second case, when markers from the same physical chromosome are separated in two or more LG and no grandparental genotype is available, it is not possible to know *a priori* which two phases are in coupling. If these LG are joined with their phases in repulsion, an expansion of the genetic distances will be observed. Here, both combinations of phases for the two joining LG were tested, and the one that provide the shorter genetic distance was selected.

When there are only two LGs per chromosome and grandparent genotypes data is unavailable, the phases remain arbitrary.

#### Ordering markers

Within each parental map, markers can be ordered by either the physical positions (synteny pipeline) or genetically.

Physical coordinates were assigned at the SNP calling step based on the *V*. *vinifera* reference genome [36] version 12X.0 [37] and then converted from the 12x.0 to 12x.2 [50] version, using one of the following approaches: For SNP coordinates, the 200 base pair context sequence flanking each SNP was retrieved from the 12x version, and was aligned to the 12x.2 version, using bwa version 0.7.5a-r405 [41]. The new coordinates were inferred from the alignment coordinates. Only uniquely aligned sequences were converted. For sequence tags, both the start and stop positions were converted, as described for SNP coordinates.

Genetic ordering of the markers was achieved by computing the minimum spanning tree of an associated graph with MSTMap [48], with the following parameters: population_type = DH, distance_function = “kosambi”, cut_off_p_value = 2, no_map_dist = 15, no_map_size = 5, missing_threshold = 0.5, estimation_before_clustering = no, detect_bad_data = yes, objective_function = ML. Following the initial ordering step, redundant markers, suspicious genotypes identified by MSTMap, and markers that resulted in double crossovers for more than 20% of progeny were discarded. The remaining markers were re-ordered with the same MSTMap parameters as above.

Maps were then converted to R/qtl’s “csvsr” format for curation and QTL mapping. Data were encoded in both the 4-way cross format (“4way”) and the backcross (“BC”) format in order to access the full functionality of R/qtl [14].

### Curation of genetic maps

Genetic maps in backcross format were independently curated in R/qtl in five steps. A detailed procedure for map curation as well as the documentation for the functions created for curation purposes are described in pipeline documentation (S1 File).

1. Markers with ≥50% missing data and individuals with either >50% missing genotypes or with twice as many crossovers as the family mean were removed.
2. For each LG, marker order was evaluated by visual inspection of rf/LOD plots in R/qtl. In cases where a group of markers were in higher linkage with non-adjacent markers, marker order was re-assigned using the R/qtl function ‘switch.order’, or re-estimated using the R/qtl function ‘orderMarkers’ after manually dropping conflicting markers identified by either high mean recombination fraction or low mean LOD related to their neighboring markers.
3. Genotyping error was determined as the value that maximizes the log likelihood estimate as described [51]. The order of backwards LGs was inverted for correspondence with the SNP’s physical position, and genetic distances for all LG were re-estimated using the Kosambi function.
4. The effect of dropping one marker at a time was estimated using ‘droponemarker’ function in a sliding window of nine markers within each LG. A LOD difference threshold was determined for each map by plotting a histogram of the results and selecting the LOD difference value that removes the upper tail of the distribution. Suspicious markers that increase genetic distances over 2 cM and had LOD difference values above the selected threshold were removed. Genetic distances and recombination fractions were re-estimated.
5. Finally, each LG was manually inspected for suspicious markers, including those creating gaps larger than 2cM or with high mean recombination fraction or low mean LOD related to their neighboring markers (S3 Table). Suspicious markers were removed, maps were rippled with a window of seven SNPs, and genetic distances were recalculated using the Kosambi mapping function.

Minor allele frequency (MAF) and minor tag frequency (MTF) were calculated for both, SNPs in final map and SNPs removed during curation using the VCF file generated at the SNP call stage. For each marker MAF was determined using—freq2 command in VCFtools [45], and MTF was calculated as the read depth of the alternative allele divided by read depth of all alleles at the locus.

### Mapping the flower sex locus

R/qtl software [14] was used to map the flower sex locus in the three F_1_ families segregating for the trait, using both synteny and *de novo* curated maps in a 4-way format. Multipoint genotype probabilities were calculated using the calc.prob function with step = 1. A one-dimension scan was performed using the scanone function, with a binomial model, the Haley-Knott regression method and an error.prob = 0.01. LOD significance thresholds were determined by permutation tests (1,000). Makeqtl and fitqtl functions were used to fit a single locus model. Locus position was refined using the refineqtl function, and the presence of additional loci was tested with the addqtl command. QTL confidence intervals were determined using a 1.8 LOD threshold.

## Results

### GBS SNP calling and family level quality control

From the 8,353 VitisGen samples included in SNP calling, 16,708,678 tags were identified. From these, 9,179,721 tags (54.94%) aligned to unique positions in the PN40024 reference genome, 1,190,427 (7.12%) aligned to multiple positions, and 6,338,530 (37.94%) could not be aligned. Overall genome coverage of all VitisGen tags uniquely aligned to the 12x.0 assembly was 13,774,386 bp, representing nearly 2.8% of it. In total, there were 301,506 sequenced intervals ranging from 12 to 979 bp in length, with a mean length of 103. Of these intervals, 262,082 (86%) were variable, resulting in a total of 1,881,000 putative SNPs from the 8,353 samples. The four F_1_ families described here had a range of 852,885 to 1,219,257 tags present in each dataset, with alignment rates ranging from 66% to 73% (S5 Table), resulting in between 300,773 and 449,840 unfiltered SNPs (S6 Table).

**Fig 2:**
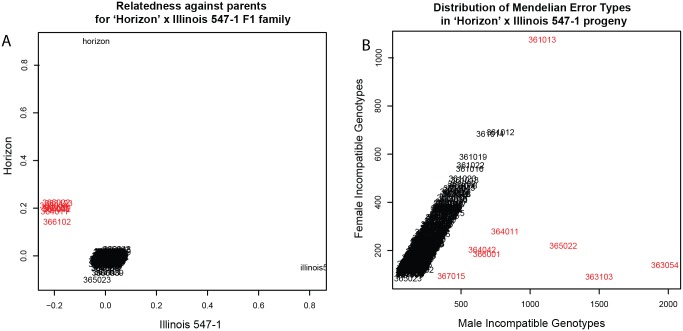
Relatedness to parents and Mendelian errors in the F_1_ family 'Horizon' x Illinois 547–1. A) Analysis of progeny relatedness to parents demonstrated that most progeny had expected relatedness values near (0,0), whereas 8 individuals were more related to ‘Horizon’ (emasculated hermaphrodite parent) and less related to Illinois547-1 (pollen parent) and were thus removed for downstream analysis. B) Mendelian error analysis indicated that 7 of these same individuals were enriched for male incompatible genotypes.

All progeny were tested for relatedness to the parents and for Mendelian errors, in order to identify pollen contaminants, self-hybridization, and mislabeling. Individuals derived from pollen contamination or self-hybridization were more related to the mother and less to the father than true progeny, as shown for eight outliers in the ‘Horizon’ x Illinois 547–1 dataset (Fig 2). Additionally, seven of these eight individuals had a high ratio of male incompatible Mendelian errors compared to female incompatible Mendelian errors. From zero to eight individuals were removed from each family, 1.7% of progeny across the families, (S3 Fig) resulting in the filtered number of progeny (S6 Table). After removing individuals, SNPs that were invariant or missing in all remaining individuals were removed, resulting in the filtered number of SNPs (S6 Table).

### Parent-independent identification of pseudo-testcross (Pt) markers

Pt markers were identified based on expected segregation of alleles (3:1) and genotypes (1:1) across progeny, filtering genotypes based on genotype quality scores to correct genotyping errors associated with heterozygote under-calling. Results were consistent across the four VitisGen F_1_ families, with a mean of 39% of SNP sites being removed due to low genotyping rate (< 50%) (Table 1). After first removing putative sequencing errors (alleles with < 5% MAF), 32% of SNPs became monomorphic. After further targeted genotype correction, 14% had incorrect segregation patterns, 12% had low genotyping rate, and only a 0.07% of the sites were rejected due to high error rate (i.e. sites with > 5% of high quality homozygote minor allele genotypes). At the end of this step, between 12,089 and 17,002 Pt markers were retained for construction of genetic maps, representing on average 4.2% of the starting number of putative SNPs.

**Table 1:** Number of SNPs after each stage of pseudo-testcross (Pt) marker identification for four F_1_ families.

Stage	*V*. *rupestris* B38 x ‘Horizon’	‘Horizon’ x Illinois 547–1	‘Chardonnay’ x *V*. *cinerea* B9	‘Horizon’ x *V*. *cinerea* B9
**Initial**	337,365	449,840	300,773	331,356
**Low genotyping rate**	119,850 (36%)	177,955 (40%)	116,467 (39%)	130,042 (39%)
**Monomorphic after sequencing error correction**	115,865 (34%)	160,424 (36%)	84,141 (28%)	92,409 (28%)
**Incorrect segregation patterns**	45,381 (13%)	43,938 (10%)	51,775 (17%)	47,630 (14%)
**Low genotyping rate after genotype error correction**	41,141 (12%)	50,169 (11%)	36,084 (12%)	45,757 (14%)
**High error rate**	290 (0.09%)	352 (0.08%)	187 (0.06%)	181 (0.05%)
**HetMappS Pt Markers Output**	14,838 (4.4%)	17,002 (3.8%)	12,089 (4.0%)	15,337 (4.6%)

### Results of HetMappS synteny pipeline

The synteny pipeline was executed using the HetMappS Pt markers identified above (Table 1). From each family, 6% of markers aligned to ‘random’ chromosomes (12X.0 version of PN40024 reference genome) were discarded. Another 5–7% were removed due to stronger genetic linkage with another chromosome than to the chromosome to which they aligned. Between 5 and 11% of markers could not be resolved, because they were correlated to multiple chromosomes, and the difference between correlations did not meet the thresholds imposed. The majority of SNPs (77–82%) were found to agree with the reference-assigned chromosome and were retained for downstream analyses (Table 2).

**Table 2:** Number of SNPs at each stage of correlation based chromosome assignment for four F_1_ families analyzed with the synteny pipeline.

Stage	*V*. *rupestris* B38 x ‘Horizon’	‘Horizon’ x Illinois 547–1	‘Chardonnay’ x *V*. *cinerea* B9	‘Horizon’ x *V*. *cinerea* B9
**Initial**	14,838	17,002	12,089	15,337
**Markers on random chromosomes**	902 (6%)	1,060 (6%)	731 (6%)	897 (6%)
**In linkage with another chromosome (“Disagree”)**	979 (7%)	1,196 (7%)	664 (5%)	901 (6%)
**In linkage with multiple chromosomes (“Unresolved”)**	1,344 (9%)	773 (5%)	1,378 (11%)	1,466 (10%)
**In linkage with aligned chromosome (“Agree”)**	11,613 (78%)	13,973 (82%)	9,316 (77%)	12,073 (79%)

**Fig 3:**
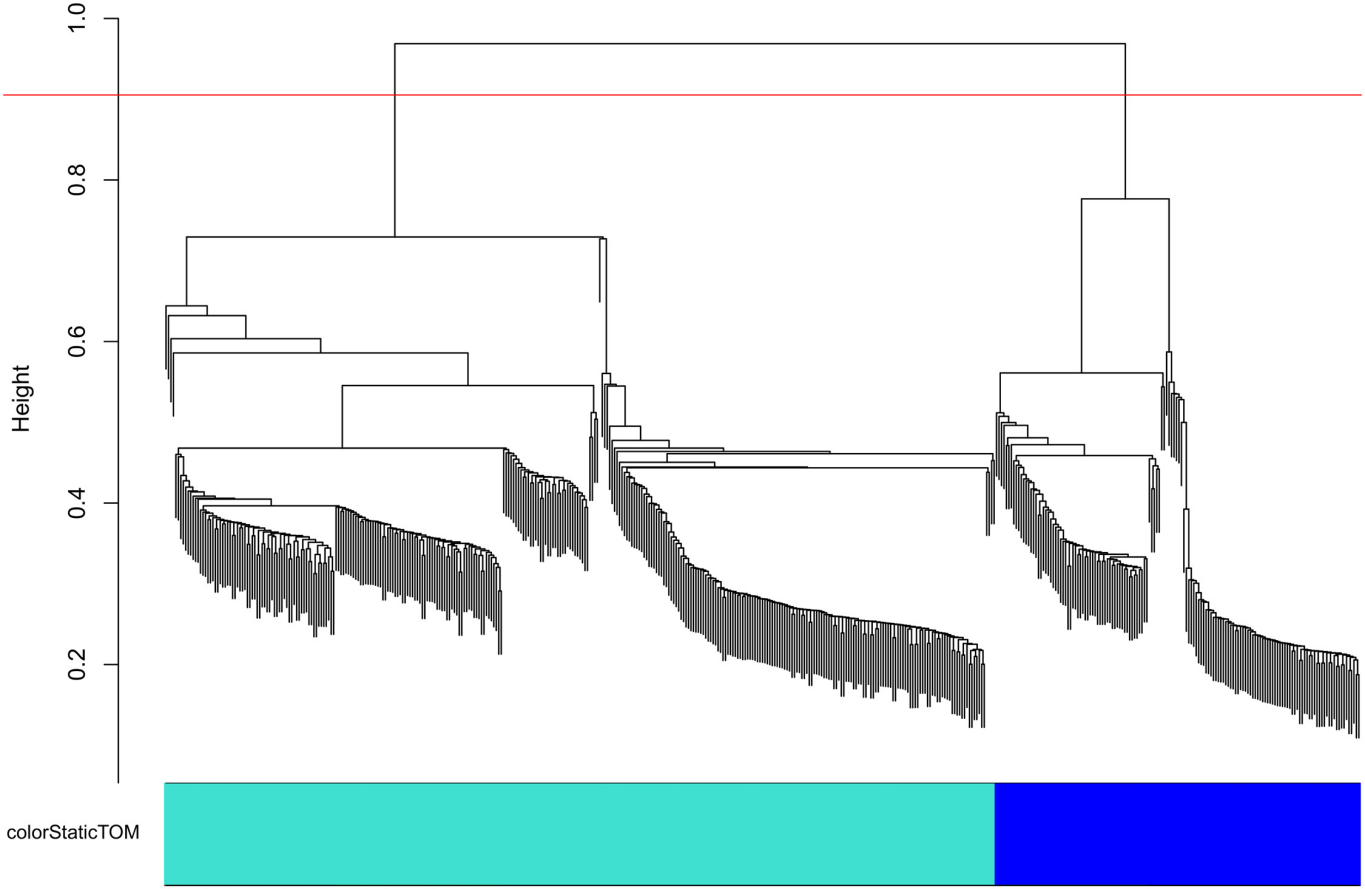
Separation of chromosomes into linkage groups (LGs) in the HetMappS synteny pipeline. This dendrogram was created from hierarchical clustering of a topological overlap matrix (as implemented by WGCNA) for markers on chromosome 2 in the F_1_ family ‘Horizon’ x Illinois 547–1. LGs result from cutting the dendrogram with height 0.9 and minimum cluster size 30, creating one LG for each parent.

Markers from each chromosome were further separated into linkage groups (LGs) corresponding to the minor allele contribution from each parent, through hierarchical clustering and dendrogram cutting (Fig 3). **A static cut of the dendrogram with height 0.9 for the VitisGen families and 0.825 for the *V*. *rupestris* B38 x ‘Chardonnay’ family, with a minimum cluster size of 30, resolved two or more LGs per chromosome.** For all VitisGen families, a static cut height of 0.9 resolved at least one LG per chromosome per parent. In some cases, chromosomal groups were split into more than one LG per parent, resulting in a larger number of LGs than the expected 38 per grapevine family (Table 3). These extra groups can be fused together at the ordering step if the true classification can be determined, either by looking for recombination fractions lower than 0.5 [51] among groups or by reference to the parental contribution of the minor allele (S3 File). Results shown here were based on the latter approach.

**Table 3:** Number of SNPs and linkage groups (LG) during LG and phase assignment for four F_1_ families analyzed with the synteny pipeline.

F_1_ family (# individuals)	# SNPs in chromosomal groups	# LGs	Split LGs (Parent)	# SNPs in phased LGs, female	# SNPs in phased LGs, male	# ordered SNPs after filtering
***V*. *rupestris* B38 x ‘Horizon’ (215)**	11,613 (78%)	41	1,4,7 (‘Horizon’)	3,449 (23%)	7,616 (51%)	5,897 (40%)
**‘Horizon’ x Illinois 547–1 (366)**	13,973 (82%)	40	1,7 (‘Horizon’)	5,666 (33%)	7,521 (44%)	9,519 (56%)
**‘Chardonnay’ x *V*. *cinerea* B9 (148)**	9,316 (77%)	38	none	4,765 (39%)	4,044 (33%)	4,482 (37%)
**‘Horizon’ x *V*. *cinerea* B9 (162)**	12,073 (79%)	40	1 (‘Horizon’)	7,503 (49%)	3,644 (24%)	5,185 (34%)

All percentages are relative to the number of Pt markers entering the synteny pipeline.

Markers from each LG were phased using hierarchical clustering and dendrogram cutting, with a static cut height of 0.9 and minimum cluster size of 30. For all LGs, markers resolved well into 2 phases (S4 Fig). No markers were removed at this stage. Following phasing, and prior to ordering, LGs with markers from same physical chromosome were identified and joined, using the parental and physical information (S3 File). In all cases it was possible to reconstruct 19 LGs per parental map. Each LG was assigned to the parent in which segregation of the minor allele was observed for the majority of the markers, as shown in the tables ‘GENOvsPARENT’ (S3 File), where the sum of heterozygous (het) alleles should be much greater than the sum of homozygous (hom) alleles.

Markers resulting from the synteny pipeline can simply retain their physical position on the reference genome [36] or can be ordered genetically. Here, we take both approaches and additionally convert tag alignment and SNP coordinates from the 12x.0 assembly [52] used for SNP call, to the more recent 12x.2 assembly [50].

Genetic ordering was performed with MSTmap [48]. After the first round of ordering, at each genetic position the marker with the most information was retained. The total number of markers per F1 family varied between 4,482 and 9,519 (Table 3), depending on the number of progeny. The number of progeny highly correlated with the total number of non-redundant markers (r = 0.99).

**Fig 4:**
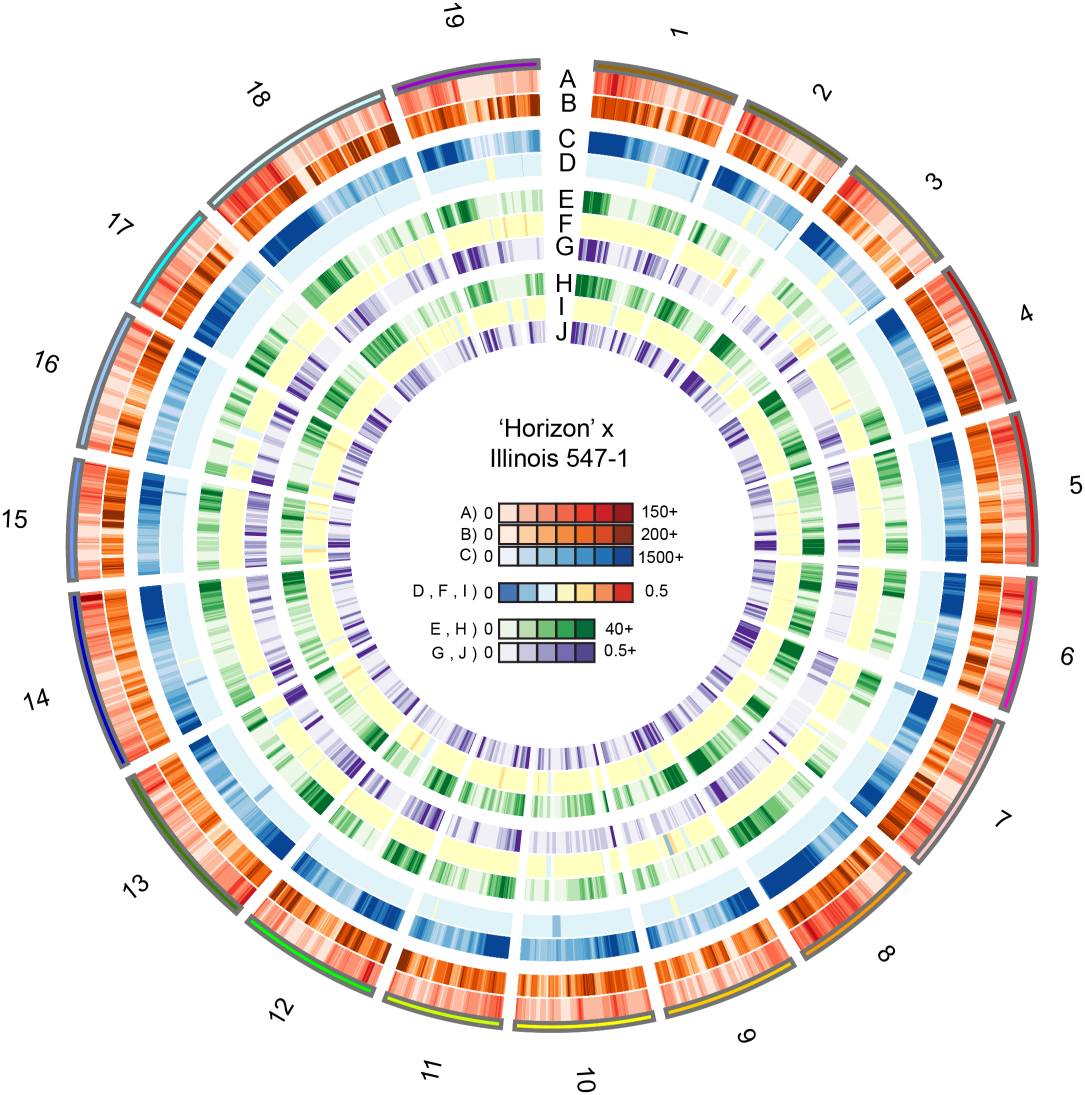
Visualization of 'Horizon' x Illinois 547–1 genomic data. Data are shown on 1 Mb windows with a 100 Kb slide. A) Number of unique tags aligned, B) Mean tag depth calculated as total tag depth over number of unique tags aligned, C) Density of SNPs entering the HetMappS pipeline, D) Minor allele frequency (MAF) of SNPs entering the pipeline, E-J) SNP output from the synteny pipeline: E) SNP density ‘Horizon’, F) MAF ‘Horizon’ SNPs, G) Recombination frequency ‘Horizon’, calculated as the number of obligate crossovers per progeny per Mb, H) SNP density Illinois 547–1, I) MAF Illinois 547–1 SNPs, J) recombination frequency Illinois 547–1, calculated as the number of obligate crossovers per progeny per Mb.

Unique tag alignment density, mean tag depth, SNP density entering the pipeline, and minor allele frequency (MAF) of SNPs entering the pipeline were visualized on 1 MB sliding windows with a 100 KB slide using Circos [53]. Additionally, for both parents, phased SNP density (output of HetMappS synteny pipeline), MAF, and recombination frequency (obligate crossovers per progeny per MB) were visualized across these windows (Fig 4, S5 Fig, S6 Fig and S7 Fig).

Minor allele frequencies for SNPs entering the pipeline were 18% for the ‘Horizon’ x Illinois 547–1 family, and between 20% and 23% for the remaining three families prior to filtering and Pt marker identification. Following Pt marker identification, MAF of Pt markers was 25±1% (Fig 4, S5 Fig, S6 Fig and S7 Fig). Minor allele frequency was also calculated across genomic windows of 1Mb size (with a 100Kb slide). The mean MAF within these windows ranged from 13% to 37%. Between 17 and 41 contiguous regions of segregation distortion (MAF < 20% or MAF > 30%) were discovered for each parental map, ranging from 0.1 to 3.1 Mb.

### Results of HetMappS *de novo* pipeline

**Fig 5:**
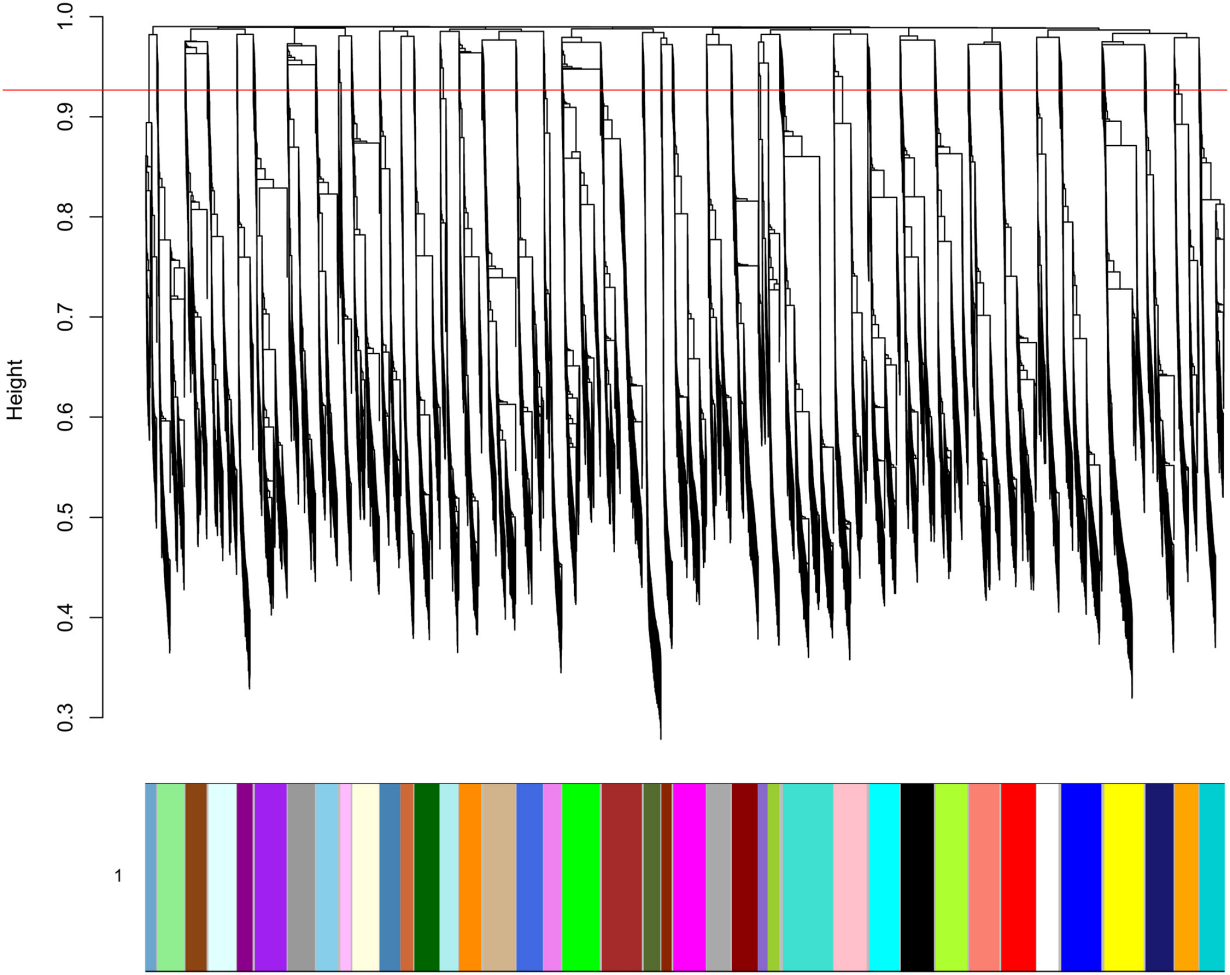
Linkage group (LG) formation in the HetMappS *de novo* pipeline. A representative dendrogram is shown for the F_1_ family ‘Horizon’ x Illinois 547–1, created from hierarchical clustering of a topological overlap matrix (as implemented in WGCNA), and subsequent cutting of the dendrogram. A cut height of 0.925 and minimum LG size of 50 resulted in 91% of the initial markers (15,464) separating into 40 LGs. Two pairs of LGs were joined in subsequent steps to create 2 parental maps with 19 LGs each.

The *de novo* pipeline was initiated using the HetMappS Pt output markers identified in Table 1. LGs were resolved by hierarchical clustering and dendrogram cutting of all Pt markers simultaneously, cutting the dendrogram (Fig 5) at a height that resulted in 38 or more LGs while maximizing the number of markers retained (S2 File) The optimal cut height varied slightly among the four VitisGen families, with values between 0.925 and 0.95, and the number of resulting LGs obtained also varied, between 39 and 43 (Table 4). These extra groups can be fused together at the ordering step if the true classification can be determined, either by looking for recombination fractions lower than 0.5 [51] among groups or by reference to the parental contribution of the minor allele as shown in the tables ‘GENOvsPARENT’ (S2 File), where the sum of heterozygous (het) alleles should be much greater than the sum of homozygous (hom) alleles, and physical location on a reference genome (S2 File, GENOvsPARENT and CHRvLGtop tabs, respectively)). Results shown here were based on the latter approach.

**Table 4:** Number of SNPs and linkage groups (LG) during LG and phase assignment for four F_1_ families analyzed with the *de novo* pipeline.

F_1_ family (# individuals)	Cut height	# LGs	Split LG (parent)	# SNPs in LGs	# SNPs phased, female	# SNPs phased, male	# ordered SNPs after filtering
***V*. *rupestris* B38 x ‘Horizon’ (215)**	0.95	43	8, 18 (*V*. *rupestris* B38) 1,4,7 (‘Horizon’)	13,829 (93%)	4,200 (28%)	8,827 (59%)	7,018 (47%)
**‘Horizon’ x Illinois 547–1 (366)**	0.925	40	1,7 (‘Horizon’)	16,115 (95%)	6,670 (39%)	8,794 (52%)	11,080 (65%)
**‘Chardonnay’ x *V*. *cinerea* B9 (148)**	0.9375	39	17 (‘Chardonnay’)	10,914 (90%)	5,513 (46%)	4,927 (41%)	5,343 (44%)
**‘Horizon’ x *V*. *cinerea* B9 (162)**	0.925	41	1,4,7 (‘Horizon’)	14,028 (92%)	8,806 (57%)	4,454 (29%)	6,221 (41%)

Percentages below SNP number are relative to the number of pseudo-testcross markers entering the *de novo* pipeline.

Markers from each LG were phased, as described for the synteny pipeline. For all F_1_ families, a static cut height of 0.9 and static min size of 10 resolved all LGs into 2 phases. Split LGs with markers from same physical chromosome were identified and joined prior to genetic ordering, using parental and physical information (S2 File). In all cases, it was possible to reconstruct 19 LGs per parental map.

Genetic ordering was performed with MSTmap [48]. After the first round of ordering, at each genetic position the marker with the most information was retained. The total number of markers per F_1_ family varied between 5,343 and 11,080 (Table 4), depending on the number of progeny. The number of progeny was highly correlated with the total number of non-redundant markers (r = 0.99).

### HetMappS analysis of a pre-VitisGen family

Prior to the implementation of standardized sampling and genotyping methods for the VitisGen project, the family *V*. *rupestris* B38 x ‘Chardonnay’ was genotyped using GBS with four key differences for the pre-VitisGen samples: 1) whole genome amplification, 2) 48-plex libraries, 3) purification and size selection with AMPure beads, and 4) SNP calling independent of the 8,353 sample VitisGen build. Given the half-sib family experimental design, we analyzed this closely-related family for comparison to the VitisGen approach.

For this pre-VitisGen family, fewer raw SNPs were identified (116,466) and more progeny (13 out of 88) were filtered out during family level quality control. While there were fewer raw SNPs for this family, 14.8% of them were retained during Pt marker identification, compared to 4.2% of the markers in the VitisGen families, resulting in 17,267 Pt markers for *V*. *rupestris* B38 x ‘Chardonnay’ (S7 Table), the highest of the five families tested. This was primarily due to fewer markers with low genotyping rate or <5% allele frequency, and fewer invariant sites after masking errors. However, this family had more markers with unexpected segregation ratios.

In the HetMappS Synteny Pipeline, only 49% of Pt markers for the *V*. *rupestris* B38 x ‘Chardonnay’ family were linked with reference-assigned chromosomes, due to frequent linkage with other chromosomes or with multiple chromosomes (S8 Table). For LG assignment, this family required a more relaxed cut height (0.825) than the VitisGen families (0.9), and nine LGs were split on the parental maps. Due to the small population size of 88 progeny, only 2,669 markers were ordered in the Synteny map (S9 Table).

In the HetMappS *de novo* Pipeline, the static cut height for the *V*. *rupestris* B38 x ‘Chardonnay’ family (0.875) was again outside the range of the VitisGen families (0.925 and 0.95), and the majority of LGs were split, resulting in 71 LGs (S9 Table). Furthermore, only the F_1_ family *V*. *rupestris* B38 x ‘Chardonnay’ lost markers (1,807) during phasing. Some clustered markers had an atypical linkage pattern not seen in the VitisGen families, resulting in five large LGs with poorly defined linkage (S8 Fig). These five LGs were removed due their atypical linkage pattern, resulting in 66 LGs in the *V*. *rupestris* B38 x ‘Chardonnay’ map (S9 Table).

### Results of genetic map curation

Genetic maps from both synteny and *de novo* pipelines were evaluated in terms of marker order and total size. Across the ten parental maps from the synteny or *de novo* pipeline, an average of 0.7 or 2.1 LGs per map (S10 Table) showed major order problems, respectively (S1 File). Further, all genetic maps derived from MSTMap showed larger genetic distances than the ~100 cM per LG that is typical for *Vitis* F_1_ families.

**Fig 6:**
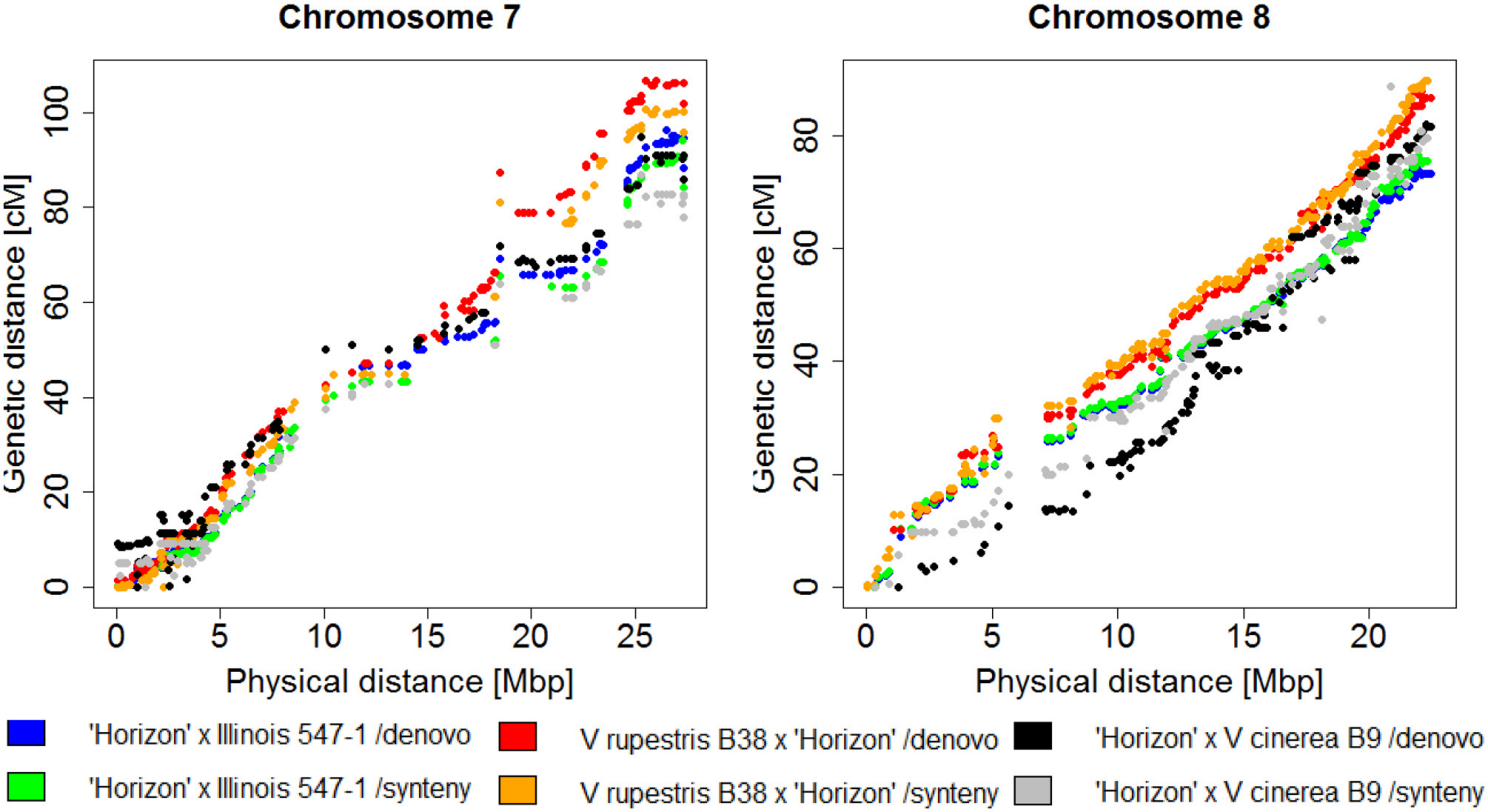
Physical position (PN40024 12X.2) vs genetic distances in six independent maps for chromosomes 7 (A) and 8 (B) of 'Horizon'. Genetic maps were generated with both synteny and de novo pipelines from three independent F_1_ families: *V*. *rupestris* B38 x ‘Horizon’, ‘Horizon’ x *V*. *cinerea* B9 and ‘Horizon’ x Illinois 547–1.

Initial genetic distances ranged from 1,891 cM (1,231 SNPs) for the female synteny map of the *V*. *rupestris* B38 x ‘Chardonnay’ family, up to 8,311 cM (4,450 SNPs) for the male *de novo* map of the ‘Horizon’ x Illinois 547–1 family (Tables 5 and 6). Map curation with R/qtl (S3 Table, S4 File and S4 Table) was effective in reducing map size in both synteny and *de novo* pipelines, from an average distance of 2,906 cM and 4,878 cM to average distances of 1,286 cM and 1,351 cM respectively (S7 File). The number of markers in the final map was highly correlated with the number of individuals in the family both synteny and de novo pipeline (r = 0.92 and 0.91, respectively). Final maps from both synteny and *de novo* pipelines resulted in good correlations between the genetic order and physical position of SNPs, and also had good coverage of the physical genome (S8 File, Fig 6).

**Table 5:** Synteny map distances and numbers of markers throughout the curation process for pre-VitisGen and VitisGen families.

F_1_ family (# individuals)		Initial # markers	Initial Genetic distance (cM)	# markers after ordering	Genetic distance (cM) after ordering	# Markers after autodrop	Genetic distance (cM)after autodrop	Final # markers	Final genetic distance (cM)
***V*. *rupestris* B38 x 'Chardonnay' (69)**	female	1,231	1,891	1,231	1,671	1,210	1,526	925	1,275
	male	1,438	2,379	1,373	2,021	1,333	1,748	1,026	1,468
***V*. *rupestris* B38 x 'Horizon' (211)**	female	2,092	2,600	2,092	2,589	2,046	2,023	1,924	1,313
	male	3,805	3,525	3,805	3,511	3,732	2,667	3,493	1,583
**'Horizon' x Illinois 547–1 (344)**	female	4,158	2,856	4,158	2,996	4,089	2,294	3,875	1,229
	male	5,361	4,462	5,361	4,695	5,264	2,604	4,992	1,211
***'*Chardonnay' x *V*. *cinerea* B9 (140)**	female	2,375	2,239	2,375	2,226	2,332	1,879	2,108	1,233
	male	2,107	2,436	2,077	2,262	2,032	1,869	1,869	1,180
**‘Horizon' x *V*. *cinerea* B9 (145)**	female	3,297	4,563	2,913	4,036	2,796	3,004	2,600	1,320
	male	1,888	2,110	1,653	1,873	1,610	1,435	1,520	1,047
**Average:**		2,775	2,906	2,704	2,788	2,644	2,105	2,433	1,286
**Percentage of Initial:**				97%	96%	95%	72%	88%	44%

**Table 6:** *De novo* map distances and numbers of markers throughout the curation process for pre-VitisGen and VitisGen families.

F_1_ family (# individuals)		Initial # markers	Initial genetic distance (cM)	# markers after ordering	Genetic distance (cM) after ordering	# markers after autodrop	Genetic distance (cM) after autodrop	Final # markers	Final genetic distance (cM)
***V*. *rupestris* B38 x 'Chardonnay' (69)**	female	1,594	3,280	1,558	2,469	1,471	1,782	1,067	1,322
	male	1,772	3,303	1,663	2,348	1,577	1,879	1,199	1,459
***V*. *rupestris* B38 x 'Horizon' (211)**	female	2,568	4,523	2,568	4,247	2,441	2,661	2,225	1,388
	male	4,450	8,312	4,450	7,880	4,268	5,681	3,889	1,696
**'Horizon' x Illinois 547–1 (344)**	female	4,857	6,547	4,784	5,873	4,635	4,194	4,316	1,286
	male	6,223	6,152	6,106	5,186	5,936	3,548	5,560	1,314
***'*Chardonnay' x *V*. *cinerea* B9 (140)**	female	2,772	3,474	2,772	3,240	2,676	2,446	2,394	1,275
	male	2,571	3,661	2,537	3,328	2,441	2,377	2,177	1,293
**‘Horizon' x *V*. *cinerea* B9 (145)**	female	3,886	5,971	3,560	3,913	3,396	2,297	3,118	1,347
	male	2,336	3,554	2,271	3,084	2,161	2,133	1,956	1,125
**Average:**		3,303	4,878	3,227	4,157	3,100	2,900	2,790	1,351
**Percentage of Initial:**				98%	85%	94%	59%	84%	28%

**Fig 7:**
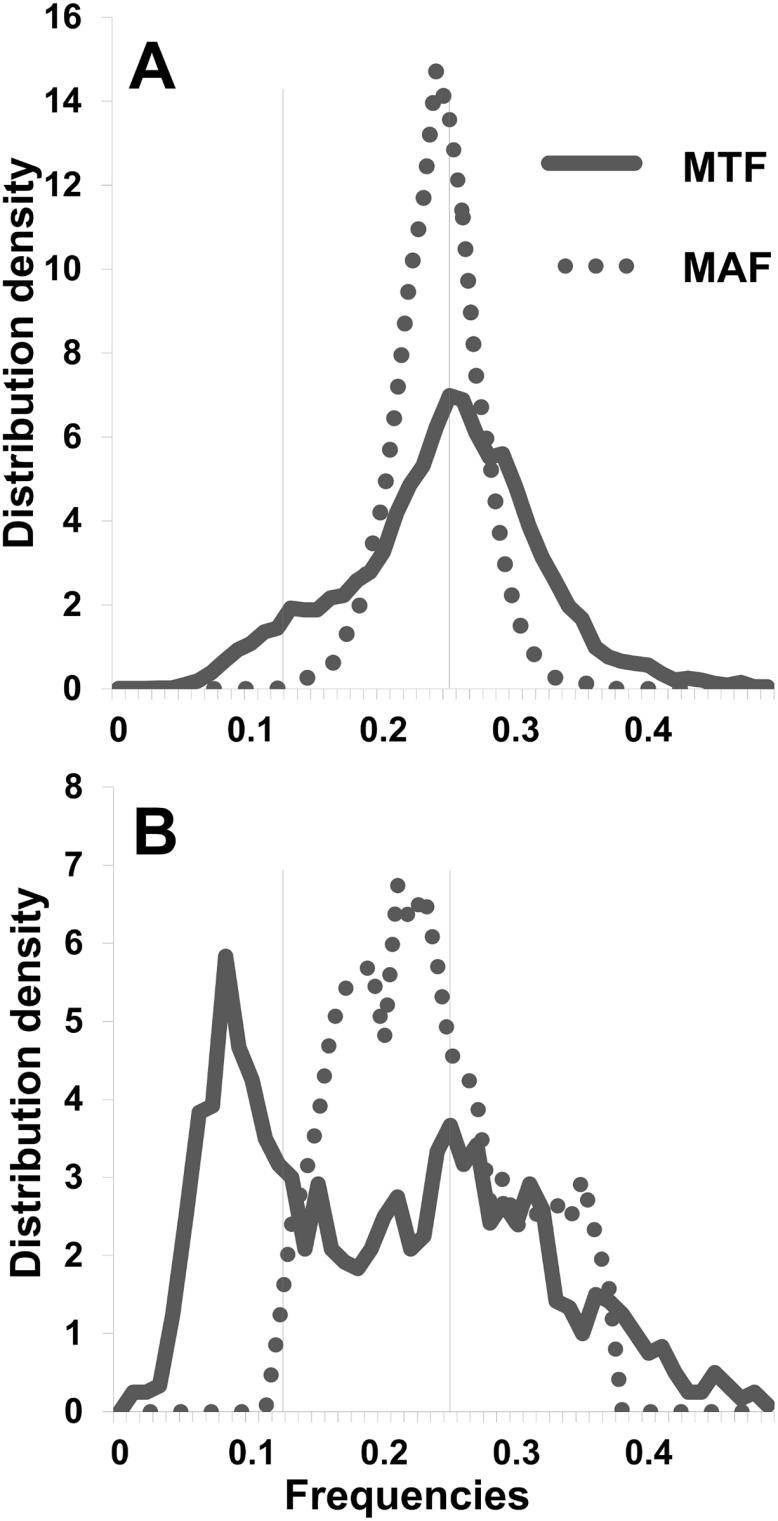
Comparison of the distribution of minor tag frequency (MTF) and minor allele frequencies (MAF) in final and spurious SNPs. MTF (continuous line) and MAF (dotted line) distributions are shown for A) the final map (9,876 SNPs) and B) markers removed during curation (1,204 SNPs) in ‘Horizon’ x Illinois 547–1 *de novo* map.

While the minor allele frequency (MAF) in the final maps showed a normal distribution centered near 0.25 (Fig 7), the minor tag frequency (MTF, measured at read depth level of alleles) suggested a bimodal distribution with peak modes of 0.25 and 0.125 (Fig 7). In contrast, markers removed during the map curation process were distributed across all MTFs with a peak lower than 0.125 and with no correlation to the MAF distribution (Fig 7).

### Localization of the flower sex locus

**Fig 8:**
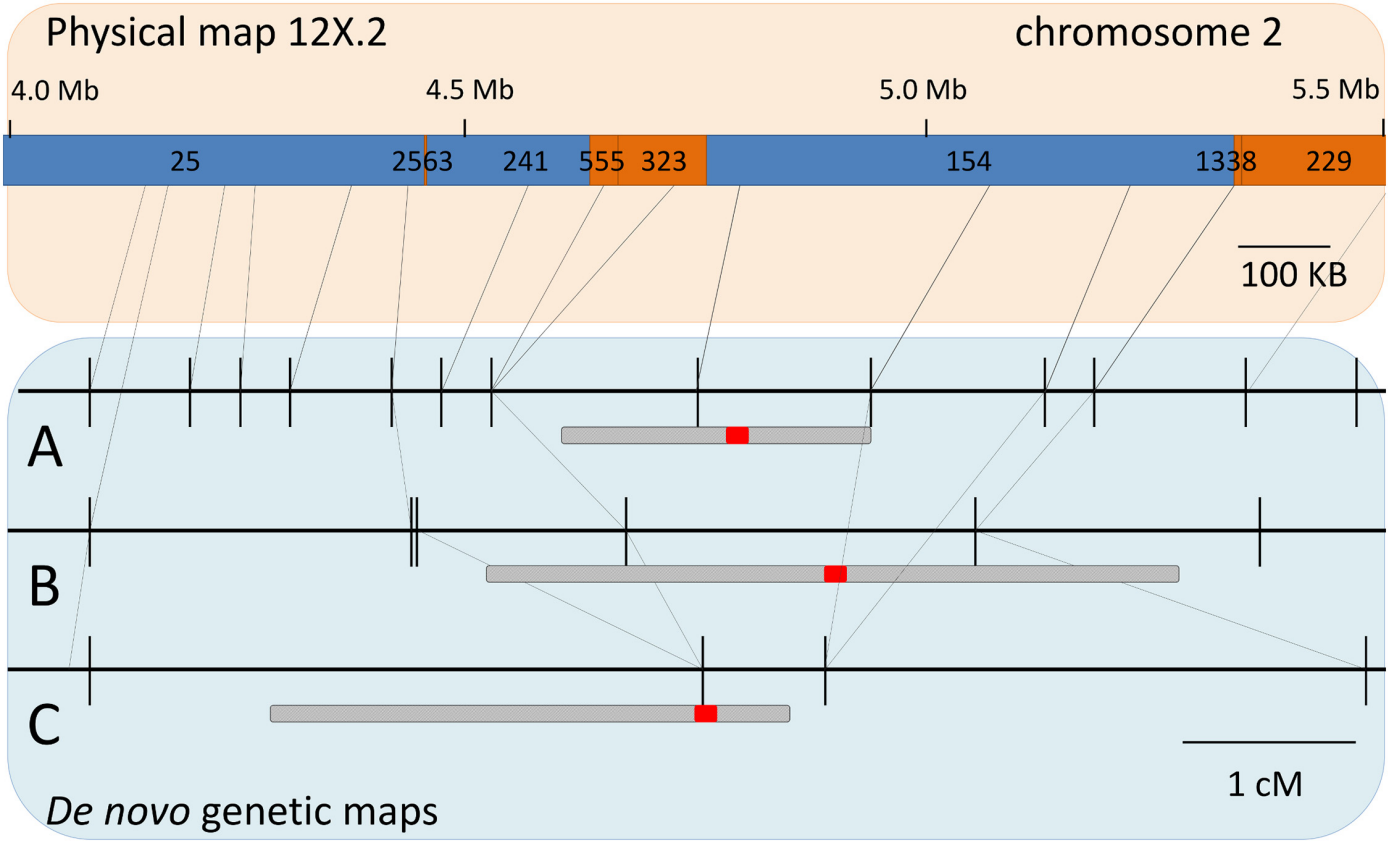
Localization of the flower sex locus using *de novo* maps from three families. The PN40024 version 12X.2 reference surrounding the sex locus is shown. Numbers above blue and orange sections indicate scaffold id. Blue scaffolds were located in chromosome 2 and orange scaffolds were located in “unknown” chromosomes in the previous version 12X.0 of PN40024. Connecting lines indicate physical position for SNPs in three *de novo* maps: A) ‘Horizon’ x Illinois 547–1, B) ‘Horizon’ x *V*. *cinerea* B9, and C) ‘Chardonnay’ x *V*. *cinerea* B9. For each map, localization of flower sex locus is shown. Shaded areas indicating 1.8 LOD confidence intervals and solid red areas indicate position of the maximum LOD.

Among the five interrelated families, flower sex segregated as a major locus following the current genetic model of dominance M > H > *f* for three families: ‘Horizon’ x Illinois 547–1, ‘Horizon’ x *V*. *cinerea* B9 and ‘Chardonnay’ x *V*. *cinerea* B9, with a 1:1 ratio of male:hermaphrodite (χ^2^(1) = 3.1935, 0.7042 and 2.3478, respectively). Crosses made with the female parent *V*. *rupestris* B38 (*f f*) showed no segregation of flower sex with all progeny being hermaphroditic, indicating that that ‘Chardonnay’ [54] and ‘Horizon’ are homozygous for the hermaphrodite allele (HH). In all three crosses and both pipelines, the flower sex locus consistently mapped to a physical position between 4.75 Mb to 5.39 Mb of the PN40024 version 12X.0 (Table 7), further supporting previous genetic analyses of flower sex [33–35]. Combining the genetic maps from these three families in alignment with the reference genome provided a higher resolution of the flower sex locus (Fig 8).

**Table 7:** Genetic mapping statistics for the flower sex locus using both synteny and *de novo* HetMappS pipelines for three F_1_ families.

F1 family (# individuals)	Map Type	LOD Peak	LOD threshold at α = 0.05	Locus Position (cM)	Nearest markers*	Confidence Interval ± 1.8 LOD (cM)	% of Variation Explained
**‘Horizon' x Illinois 547–1**	*de novo*	96.1	3.26	25.0	S2_4855222	24.0–25.8	72.8
	synteny	95.8	3.26	25.5	S2_4855222	24.0–27.0	72.7
**‘Horizon' x *V*. *cinerea* B9**	*de novo*	39.3	3.29	29.0	S2_5125806- S2_5390838	27.0–31.0	70.8
	synteny	38.6	3.23	27.0	S2_5125806- S2_5390838	25.1–31.0	70.2
**‘Chardonnay' x *V*. *cinerea* B9**	*de novo*	42.8	3.28	30.4	S2_4745220	28.0–31.0	74.8
	synteny	39.7	3.13	26.0	S2_4745220- S2_5121461	25.0–30.0	72.2

* Single or two flanking markers are provided. Marker name indicates the position on the physical PN40024 genome (version 12X.0) in format S(chromosome)_(position in bp).

## Discussion

The modular HetMappS pipeline handles large datasets for heterozygous F_1_ families and provides corrections for common GBS problems like large proportion of missing data and heterozygote under-calling. The method does not rely on parental genotypes, although they can be utilized for validation. The choice between synteny and *de novo* linkage group (LG) formation allows the leveraging of existing genome information where available, but also creates a viable option to develop genetic maps for families that include complex hybrid backgrounds and wild species. Unlike previous methods [10, 11], the *de novo* option can handle a large number of markers for LG creation without the aid of a reference genome. The pipeline is modular, and it can be combined in total or in portion with other filtering or ordering strategies.

Starting from 300,000 to 450,000 markers of variable quality, we were able to create saturated maps with genetic distances comparable to standard SSR-based maps [21], containing up to 20 times more markers (4,992 and 5,560 SNPs for the synteny and *de* novo pipelines respectively). The number of markers in the final map was highly correlated with the number of individuals in the family both for the synteny pipeline and *de novo* pipeline (r = 0.92 and 0.91, respectively). This suggests that recombination events (hence, the number of progeny genotyped) are currently the limiting factor for the creation of saturated genetic maps in crosses of heterozygous parents.

### GBS SNP calling

Here we used the *V*. *vinifera* reference genome for alignment of tags, representing a reduced section (2.8%) of the physical assembly. This approach of analyzing 64 bp tags generated by *Ape*KI (theoretically cutting every 1024 bp) could be expected to represent 6.2% of the physical assembly. The reduced number of tags could be due to PCR bias, imperfect restriction digestion, methylation, repetitive sequences resulting in multiple alignment of tags, and non-reference sequences. For the four VitisGen families, alignment rates ranged between 66 to 72% of the tags aligning to a unique position of the reference genome, 11 to 12% were multiply aligned, and between 16 to 22% of the tags were unaligned (S5 Table). Unaligned tags can contain poor quality reads and low complexity sequences, but can also contain divergent or species-specific sequences that are not represented in the reference genome. This unaligned pool of tags could be re-analyzed when additional reference genomes become available that are more relevant to the mapping families. Previously, a genotyping microarray revealed similar but more extreme challenges resulting from the large genetic diversity of Vitis [55]. The vast majority (over 85%) of SNP microarray markers designed based on *V*. *vinifera* sequences were not informative in each breeding population. Thus, GBS appears superior to SNP microarrays for high-diversity species and will increase in utility as additional genomes are sequenced.

The distribution of GBS tags across the genome showed a remarkable consistency for the four families (Fig 4, S5 Fig, S6 Fig and S7 Fig). Tags were concentrated in the arms of the chromosomes and sparse in centromeric regions, likely due to repetitive sequences in centromeric regions that were multiply aligned and enrichment of methylated sequences that were not digested by *Ape*KI. Alternative explanations for lower proportion of tags in some regions of the reference genome could be divergence between the wild and hybrid vines with the *V*. *vinifera* genome. There are also regions with higher proportion of tags (chromosomes 8, 15, 18 and 19) that are consistent among the families. Areas with high mean tag depth were observed (Fig 4, S5 Fig, S6 Fig and S7 Fig). One possibility is that these regions indicate repeated (but uniquely aligning) fragments in the wild and hybrid *Vitis* genomes that are not represented in the *V*. *vinifera* reference genome.

### Quality control and identification of Pt markers

Family-level quality control is an essential step, given that marker identification is based on progeny segregation patterns, and LG formation and phasing are heavily influenced by the amount of linkage present. Individuals who do not share the same parents will distort segregation ratios and dilute the linkage patterns, thereby reducing resolution.

Because of shallow sequence coverage, the error rate associated with heterozygote under-calling is high, which is problematic both for linkage analysis and for identifying, phasing, and ordering markers. Genotype filtering in HetMappS was tailored to retain the maximum amount of high-quality genotypes and improve the ability to order markers [56]. The vast majority of SNPs were filtered due to low genotyping rate (51%) and putative sequencing error (32%). Only 13% of the markers were filtered due to non-Pt segregation ratios and just 0.07% for high error rate (ie, homozygous for minor allele with GQ>98). These results are consistent with the expected output for the GBS technique. With only a 4.2% of the initial markers being retained for linkage map creation, our pipeline was effective at reducing the number of markers in a single step, with decisions based largely on SNP quality. On average, loci had 6.0% of genotypes corrected (sd 10%), and 21.4% of sites masked (sd 31.3%). This could be explained by sequencing error or heterozygote under sampling.

Pt marker coverage correlates well with SNP coverage (r = 0.86, Table 1). Some regions had a lower than expected number of Pt markers for one or both parents based on the mean Pt and SNP coverage across windows, although those regions with less than 10% of the mean Pt marker coverage were primarily low coverage regions. Of the 622 regions across 8 parental maps where Pt marker coverage was less than 10% of the expected number, SNP coverage was at most 17% of the mean number of SNPs per 1Mb window, and 98% were under 10% (S5 File).

### HetMappS pipelines

Genetic maps generated were consistent between pipelines within populations, as well as maps for the common parent in the half-sib families examined (Fig 6, S7 File, S8 File). After LG formation, phasing and ordering, the *de novo* pipeline retained on average an extra 7% of markers compared to the synteny pipeline (49.3% and 42.3%, respectively). This can be attributed to markers located on unassembled chromosomes (6.06%) or markers that were assigned to a different chromosome during the alignment step (6.31%), since both of these categories were filtered previous to LG formation in the synteny pipeline. However, the *de novo* pipeline had more apparent problems with initial ordering (Tables 5 and 6, S10 Table). The *de novo* approach should be useful when there are major structural variations relative to the reference genome, as it makes no assumptions about synteny with the reference genome. However, depending on the amount of linkage between markers, the *de novo* pipeline may be more error-prone for LG creation and may require more downstream filtering. There is greater control associated with the choice of dendrogram cutting options using the *de novo* pipeline, and while more stringent cut parameters ensure fewer problematic markers, it can also result in complete or partial loss of LGs. Thus, the specific filtering required will depend on the family and on user preferences related to tradeoffs between the number of retained markers and LGs versus the extent of curation required.

Dendrogram cut height in the synteny and *de novo* pipelines may explain subtle differences in the maps obtained for each pipeline. In both pipelines, dendrograms were cut with a static height across each set of Pt markers. In synteny LG formation, markers belonging only to 19 chromosomal groups are identified and separated previous to dendrogram formation, hence 19 dendrograms were cut at the same height to determine parental segregation within each chromosome (Fig 3). This two-step process contrasts with the *de novo* pipeline, where all 38 chromosomes need to be separated by a static cut of the same dendrogram (Fig 5). In most cases, the expected 38 LGs were not resolved perfectly by the *de novo* pipeline (Table 4); some LGs were fused while others split in two, reflecting technical and biological realities. For example, while some chromosomes were only split in one pipeline, chromosome 1 and 7 in the parental maps of ‘Horizon’ tended to split into two LGs in both the *de novo* pipeline (Table 4) and the synteny pipeline (Table 3). The gaps observed in some LGs may be related with the evolutionary history of the *Vitis* genome; for example, the bottom part of chromosome 7 in *V*. *vinifera* corresponds to the extra chromosome found in the *V*. *rotundifolia* [57, 58], although none of the families involved in this study include *V*. *rotundifolia*.

Correlations between results obtained independently from the synteny and *de novo* pipelines suggests that both methods are robust for map creation, and that the maps generated from the two pipelines correspond well in most cases.

For each family, the total number of SNPs retained could be explained by factors like number of progeny or dendrogram cut height. However, for parental maps within each family, hybrid parents retained a higher proportion of markers (51.3, 48.9, 44.2, and 33.3%), compared with wild species (23.2, 23.8, and 33.5%), and *V*. *vinifera* ‘Chardonnay’ was intermediate (39.4%, Table 3). This likely reflects the better suitability of the PN40024 reference genome for *V*. *vinifera* alleles versus wild *Vitis* as well as greater genetic diversity within hybrids. Lack of diversity within *V*. *vinifera* cultivars has been shown previously [24].

### Curation of genetic maps

Genetic maps often require manual curation as spurious markers can distort genetic distances and marker order. This process cannot be fully automated, as LOD scores for each marker may vary along the LGs, especially at the extremes of chromosome arms, where markers are in weaker linkage than SNPs located at the center of the chromosome [51]. With hundreds of markers per LG it is not feasible to examine site by site. In order to standardize the process we followed a five-step procedure for map curation and developed scripts to ease this step.

With average distances of 2,906 and 4,878 cM, non-curated genetic maps generated from synteny or *de novo* pipelines were larger than expected. As reference, parental grapevine SSR maps span between 1,172 and 1,406 cM [21], while an integrated SSR map of 257 SSR markers in five populations had a total length of 1,485 cM [57]. A dense map of 994 SNP loci in a *V*. *vinifera* cross showed a total length of 1,245 cM [59]. Other maps created with RAD or GBS were between 1,381 and 1,967 cM [10, 28].

Presence of spurious markers was the main cause of map inflation. In the auto drop step, removing an average of 5% and 6% of markers (synteny and *de novo* maps, respectively) led to an average map reduction of 24% and 26% of the initial genetic length. SNPs located at the extremes of the LG were not automatically removed to avoid unnecessary trimming of the maps. Manually removing an extra 7% and 10% of markers (synteny and *de novo* maps, respectively) led to further average reduction of genetic length of 28% and 31%. Prior to these, the ordering step had minimal impact, both in terms of markers removed (3% and 2% in synteny and *de novo* maps, respectively) and genetic distance reduction (4% and 15%). This suggests that the minimization of an associated spanning tree implemented by MSTMap provided a good marker order, even in data sets with high percentage of missing data (up to 50% for these datasets) [48].

As expected, final maps distances were not correlated with the number of progeny or with the number of markers in the final synteny or *de novo* maps (r = -0.13 and r = 0.00, respectively). In contrast, the number of progeny was highly correlated with the number of markers retained for each map (r = 0.92 and 0.91, respectively). Total genetic distances in final maps averaged 1,286 cM for 2,433 loci in the synteny pipeline and 1,351 cM for 2,790 loci in the *de novo* pipeline.

### Repeated genome regions

Tags located in repeated regions can produce marker artifacts. Identical PCR products originating from repeated regions will collapse into one single tag, representing only one allele in the genotype output. SNPs associated with these may not show distortion at the allele frequency level. In order to identify these markers, it is necessary to consider the frequency of the read depth for the minor allele, as a proxy for the frequency of the minor tag within a locus, contained in the VCF file.

While the minor allele frequency (MAF) in the final maps showed a normal distribution centered near 0.25 (Fig 7), the minor tag frequency (MTF) suggested a bimodal distribution with larger variance (Fig 7). The predominant MTF mode peak was centered in 0.25, which is consistent with the observed MAF. The peak for a smaller second mode was located around 0.125, which is consistent with 1:7 segregation expected for a minor tag located in one of two duplicated regions. This second peak was not observed in the MAF distribution (Fig 7), suggesting that most of the repeats underlying these 1:7 MTFs on the final map were tightly linked and inherited as normal Pt markers.

Markers removed during the map curation process were distributed across all MTFs, with a peak at frequencies lower than 1:7. The MAF distribution (Fig 7) of these markers had a broad distribution from 0.1 to 0.4 and did not correlate with the MTF distribution. This result suggests that a proportion of spurious markers may be located in repeated regions that are not represented in the PN40024 genome. Hence, the associated tags were not removed after the alignment step. Markers located in repeated segments or markers in tandem regions of more than two repeats could inflate genetic distance calculations due to occasional recombination within tandem repeats or independent assortment.

### The grapevine flower sex locus

We used the flower sex locus to demonstrate the utility of both synteny and *de novo* maps in three populations segregating for the trait. Overall, the localization of markers nearest to the QTL peak marker was between 4.75 and 5.39 Mbp (Table 7). In the largest population a SNP located at 4.85 Mbp had the highest LOD score, very close to locations reported by fine mapping and BAC sequencing: between 4.91 and 5.05 Mbp [34] and between 4.89 and 5.04 Mbp [35], respectively. Correspondence between the genetic order and physical order of the markers in the sex locus was high (Fig 8). In maps generated by the *de novo* pipelines, SNPs located in un-assembled scaffolds from 12x.0 version of the genome were incorporated solely by linkage. A comparison with the newest 12x.2 assembly corroborated the inclusion of scaffolds 2563, 555, 323, 1338 and 229 in chromosome 2. Furthermore, our results suggest that two small additional scaffolds, 1,344 and 1,682, belong to the sex locus (S9 File). According to gene annotations in version 12X.2 of the PN40024 reference genome [60], these scaffolds contain three genes: VIT_200s1344g00010 (unknown protein), VIT_200s1682g00020 (cytochrome p450) and VIT_200s1682g00010 (primary amine oxidase). GBS markers associated with scaffold 233 did not map to chromosome 2, even though this scaffold was previously localized in the sex locus region [34, 35].

Analysis of the sex locus demonstrates the power of our approach to leverage linkage, sequencing, and alignment to the reference genome to quickly characterize a locus. SNPs anchored to the reference genome allowed us to correlate the physical position with the LOD peak without the need of BAC clones, while the *de novo* pipeline allowed incorporation of scaffolds that were not assembled in the reference genome. Further, divergent sequences discarded during read alignment and SNP calling could be analyzed to leverage more information once the region has been narrowed down.

Many traits of interest in breeding applications are introgressed from wild species. In grapevine this is exemplified by adaptive traits like disease resistance or abiotic stress tolerance. Despite occasional physical gaps in our maps, our results show that it is possible to obtain SNPs across the whole genome in crosses involving wild species. Divergent regions will still be in linkage with SNPs from common loci in a F_1_ cross, as linkage usually extends through a long portion of the chromosomes. After curated framework maps are created, additional markers may be added.

### Technical considerations

The optimal parameters for marker identification, LG formation and phasing, and filtering steps may vary depending not only on the underlying biology of the data set, but also on technical aspects of the dataset, such as genotyping method, depth of coverage, method of DNA preparation and enzyme choice. For example, the pre-VitisGen population *V*. *rupestris* B38 x ‘Chardonnay’ differed in several key aspects from the other four families. The sequence coverage for this family was higher, and DNA was prepared with a whole genome amplification (WGA) step. The WGA protocol results in a loss of methylation. Because the enzyme used here for genome complexity reduction, *ApeKI*, is a methylation sensitive enzyme, some tags may have targeted inactive transposons, repetitive regions, centromeres, or other regions that are usually methylated. The pre-VitisGen approach resulted in more Pt markers, but also more markers with unexpected segregation ratios, an atypical linkage pattern, and split LGs. Because both parents were used for VitisGen maps, we conclude these complications were more likely due to technique than to biology.

In summary, HetMappS is a publicly-available, modular approach that overcomes the limitations of applying GBS for genetic mapping in highly heterozygous species. The synteny and *de novo* pipelines generate similar maps that match the physical genome with a 5- to 20-fold increase in marker resolution relative to existing grapevine genetic maps. HetMappS can accelerate the discovery of candidate genes underlying traits and enhance the accuracy of genome-wide marker assisted selection in breeding programs, and can be adapted for other applications.

## Supporting Information

S1 Fig Crosses resulting in five biparental F_1_ families. Crosses are indicated with solid lines and number of progeny (N). Pedigree relationships are indicated with dashed lines.
(TIF)

S2 Fig Pipeline schematic for identification of pseudo-testcross markers. Gray boxes are optional steps if progenitors are genotyped. Red text shows user definable parameters.
(TIF)

S3 Fig Relatedness to parents and Mendelian errors in three F_1_ families. Results are shown for three VitisGen families: I) ‘Chardonnay’ x *V*. *cinerea* B9, II) ‘Horizon’ x *V*. *cinerea* B9, and III) *V*. *rupestris* B38 x ‘Horizon’. A) Analysis of progeny relatedness to parents demonstrated that most progeny had expected relatedness values near (0,0), but with some being more related to the female parent than the male parent. B) Mendelian error analysis indicates that the same individuals were enriched for male incompatible genotypes. Thus, these individuals were likely derived from pollen contamination or self-hybridization and were removed for downstream analysis.
(TIF)

S4 Fig Clustering analysis to assign SNPs to phase for each parental chromosome. Dendrograms and heatmaps for linkage groups corresponding to chromosome 2 of ‘Horizon’ and Illinois 547–1 determined by two independent strategies: synteny and de novo pipelines, showing clear resolution of two phases.
(TIFF)

S5 Fig Visualization of *V*. *rupestris* B38 x ‘Horizon’ genomic data. Data are shown on 1 Mb windows with a 100 Kb slide. A) Number of unique tags aligned, B) Mean tag depth calculated as total tag depth over number of unique tags aligned, C) Density of SNPs entering the HetMappS pipeline, D) Minor allele frequency (MAF) of SNPs entering the pipeline, E-J) SNP output from the synteny pipeline: E) SNP density ‘Horizon’, F) MAF ‘Horizon’ SNPs, G) Recombination frequency ‘Horizon’, calculated as the number of obligate crossovers per progeny per Mb, H) SNP density Illinois 547–1, I) MAF Illinois 547–1 SNPs, J) recombination frequency Illinois 547–1, calculated as the number of obligate crossovers per progeny per Mb.
(TIF)

S6 Fig Visualization of ‘Chardonnay’ x *V*. *cinerea* B9 genomic data. Data are shown on 1 Mb windows with a 100 Kb slide. A) Number of unique tags aligned, B) Mean tag depth calculated as total tag depth over number of unique tags aligned, C) Density of SNPs entering the HetMappS pipeline, D) Minor allele frequency (MAF) of SNPs entering the pipeline, E-J) SNP output from the synteny pipeline: E) SNP density ‘Horizon’, F) MAF ‘Horizon’ SNPs, G) Recombination frequency ‘Horizon’, calculated as the number of obligate crossovers per progeny per Mb, H) SNP density Illinois 547–1, I) MAF Illinois 547–1 SNPs, J) recombination frequency Illinois 547–1, calculated as the number of obligate crossovers per progeny per Mb.
(TIF)

S7 Fig Visualization of ‘Horizon’ x *V*. *cinerea* B9 genomic data. Data are shown on 1 Mb windows with a 100 Kb slide. A) Number of unique tags aligned, B) Mean tag depth calculated as total tag depth over number of unique tags aligned, C) Density of SNPs entering the HetMappS pipeline, D) Minor allele frequency (MAF) of SNPs entering the pipeline, E-J) SNP output from the synteny pipeline: E) SNP density ‘Horizon’, F) MAF ‘Horizon’ SNPs, G) Recombination frequency ‘Horizon’, calculated as the number of obligate crossovers per progeny per Mb, H) SNP density Illinois 547–1, I) MAF Illinois 547–1 SNPs, J) recombination frequency Illinois 547–1, calculated as the number of obligate crossovers per progeny per Mb.
(TIF)

S8 Fig Atypical linkage pattern in pre-VitisGen family *V*. *rupestris* B38 x 'Chardonnay'. Dendrogram created from hierarchical clustering of topological overlap matrix for SNPs derived from whole genome amplified DNA of the *V*. *rupestris* B38 x ‘Chardonnay’ F_1_ family, displaying an atypical linkage pattern. This dendrogram was cut at 0.875 height and 5 linkage groups were discarded before proceeding to the phasing step.
(TIFF)

S1 File HetMappS Pipeline Documentation. (DOCX)

S2 File *De novo* pipeline tables. (XLSX)

S3 File Synteny pipeline tables. (XLSX)

S4 File Droponemarker LOD difference distributions used for choosing LOD thresholds for marker filtering in the 'autodrop' curation step. (PDF)

S5 File Regions of low SNP and marker coverage in parental maps. SNP coverage and Pt marker coverage on 1Mb windows (calculated with 100KB slide) against the PN40024 reference genome version 12x.2 for each parental map.
(XLSX)

S6 File Regions of segregation distortion in parental maps. Regions of segregation distortion (MAF < 20% or MAF > 30%) on 1Mb windows (calculated with 100KB slide) against the PN40024 reference genome version 12x.2 for each parental map.
(XLSX)

S7 File Final genetic maps for five half sib families. Genetic maps were independently generated using both synteny and *de novo* HetMappS pipelines.
(PDF)

S8 File Comparison of physical position and genetic distances for independent maps of five progenitors. Genetic map were independently generated using synteny and *de novo* pipelines over different sets of progeny.
(PDF)

S9 File LOD profiles for flower sex phenotype mapped into chromosome 2 of the male parent. Six LOD profiles for de novo (dn) and synteny (syn) maps in three segregating families: ‘Horizon’ x Illinois 547–1 (HI), ‘Horizon’ x V. cinerea B9 (HV) and ‘Chardonnay’ x V. cinerea B9 (CV). Each column represents either a SNP or a pseudo marker generated in R/qtl. SNPs names follows the format S(chromosome number)_(physical position in bp) in version 12X.0 of the PN40024 reference genome. Pseudo markers follow the format c(linkage group number).loc(genetic distance in cM).
(XLSX)

S10 File Static version of the scripts described here, as of manuscript submission. Readers are encouraged to instead use the HetMappS repository for the latest releases and bug fixes at https://bitbucket.org/khyma/hetmapps.
(ZIP)

S1 Table Sequences and barcodes comprising four 96-plex adapter sets used to generate 384-plex GBS libraries (DOCX)

S2 Table TASSEL-GBS plugins (Glaubitz et al., 2014) used in the current study. (DOCX)

S3 Table rfSummary example for manual curation, indicating dropped markers. (XLSX)

S4 Table Selected LOD difference thresholds from 'Droponemarker' function, and markers removed during the auto-filter stage of map curation. (DOCX)

S5 Table Summary of GBS sequence tags and alignment results for four F_1_ families. (DOCX)

S6 Table Summary of GBS datasets before and after family level quality control. (DOCX)

S7 Table Pseudotestcross marker identification in a pre-VitisGen sample. (DOCX)

S8 Table Correlation based chromosome assignment in the synteny pipeline, for the pre-VitisGen *V*. *rupestris* B38 x 'Chardonnay' F_1_ family. (DOCX)

S9 Table Linkage group (LG) and phase assignment for the pre-VitisGen *V*. *rupestris* B38 x 'Chardonnay' F_1_ family, analyzed with the synteny and the de novo pipelines. (DOCX)

S10 Table Linkage groups with suspicious orders from the HetMappS pipelines, detected at genetic map curation. (DOCX)
