# Supplementary figures and images for: Heterozygous Mapping Strategy (HetMappS) for High Resolution Genotyping-By-Sequencing Markers: A Case Study in Grapevine
Source: PLoS One. 2015 Aug 5;10(8):e0134880. doi: 10.1371/journal.pone.0134880 (PMC4526651; doi:10.1371/journal.pone.0134880)

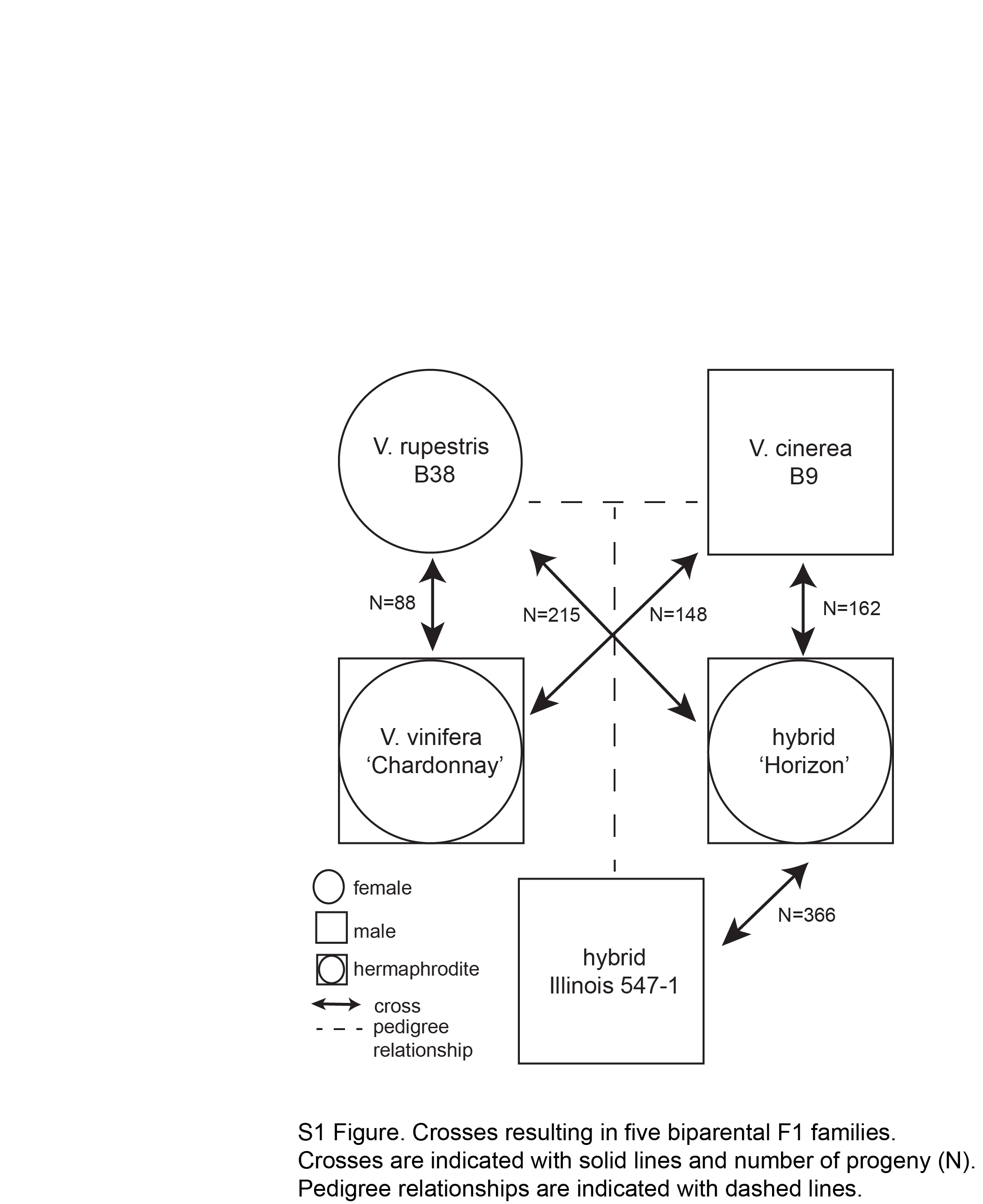

Supplement: S1 Fig — Crosses are indicated with solid lines and number of progeny (N). Pedigree relationships are indicated with dashed lines. (TIF) [file pone.0134880.s001.tif]

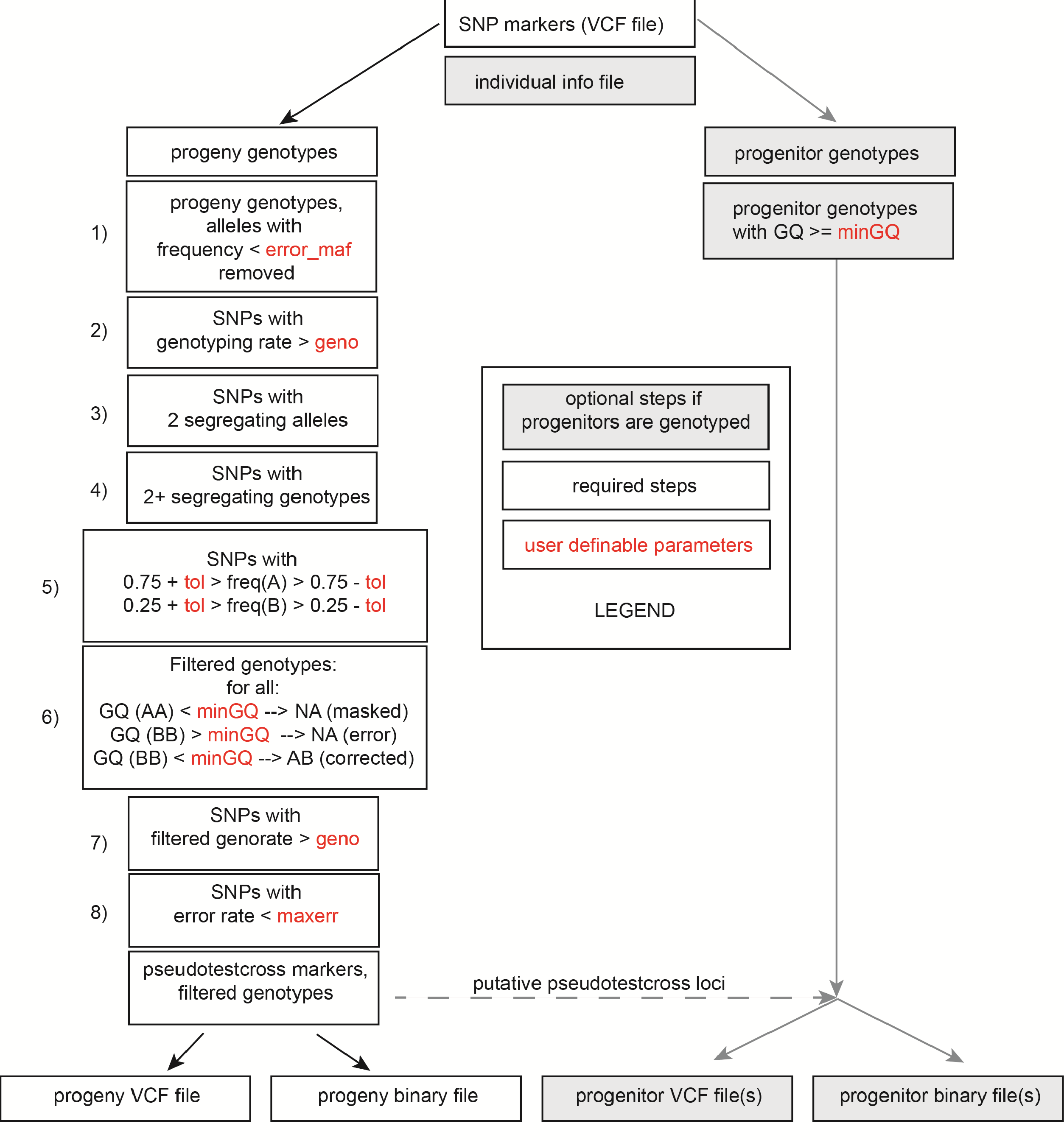

Supplement: S2 Fig — Gray boxes are optional steps if progenitors are genotyped. Red text shows user definable parameters. (TIF) [file pone.0134880.s002.tif]

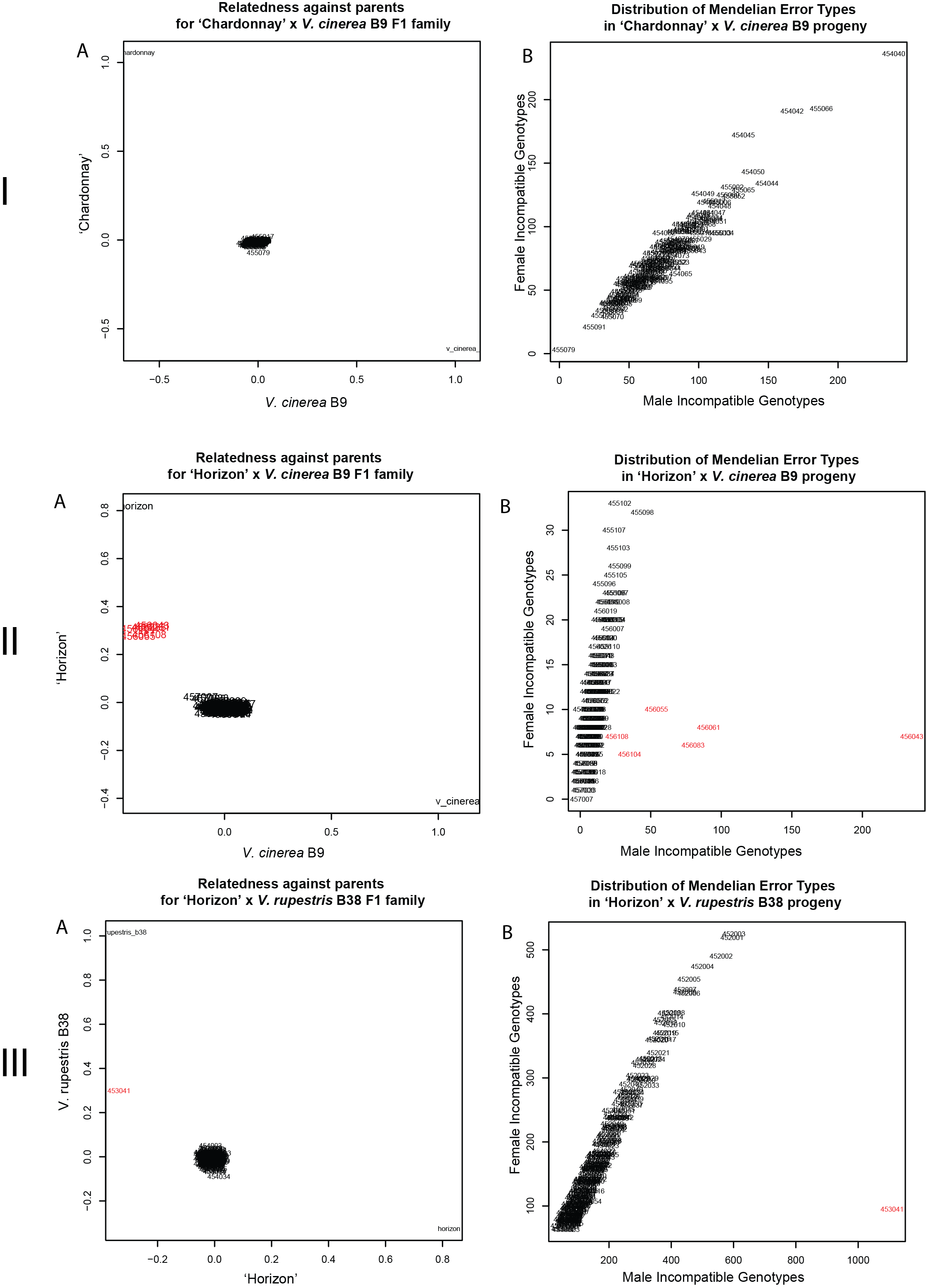

Supplement: S3 Fig — Results are shown for three VitisGen families: I) ‘Chardonnay’ x V. cinerea B9, II) ‘Horizon’ x V. cinerea B9, and III) V. rupestris B38 x ‘Horizon’. A) Analysis of progeny relatedness to parents demonstrated that most progeny had expected relatedness values near (0,0), but with some being more related to the female parent than the male parent. B) Mendelian error analysis indicates that the same individuals were enriched for male incompatible genotypes. Thus, these individuals were likely derived from pollen contamination or self-hybridization and were removed for downstream analysis. (TIF) [file pone.0134880.s003.tif]

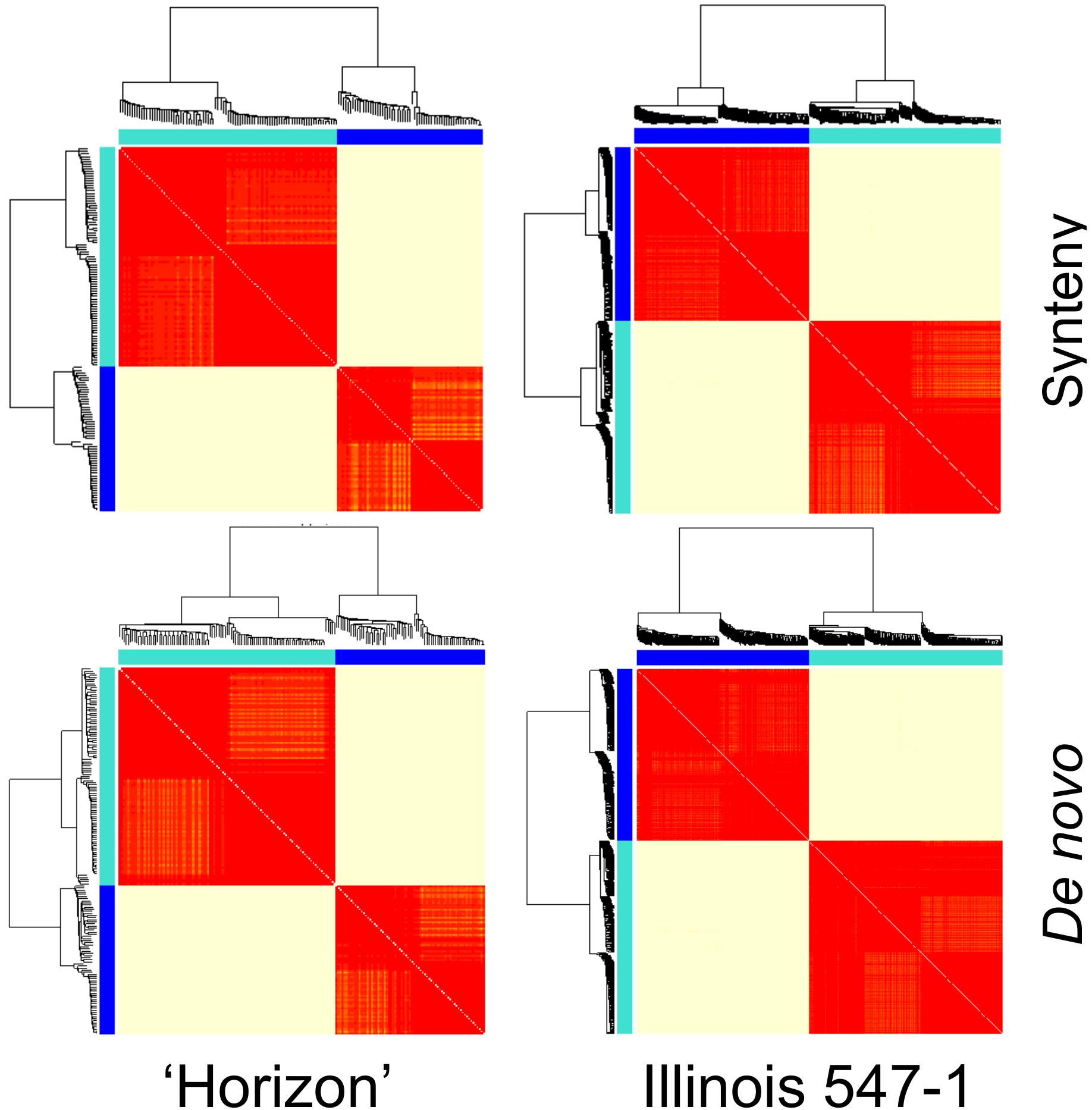

Supplement: S4 Fig — Dendrograms and heatmaps for linkage groups corresponding to chromosome 2 of ‘Horizon’ and Illinois 547–1 determined by two independent strategies: synteny and de novo pipelines, showing clear resolution of two phases. (TIFF) [file pone.0134880.s004.tiff]

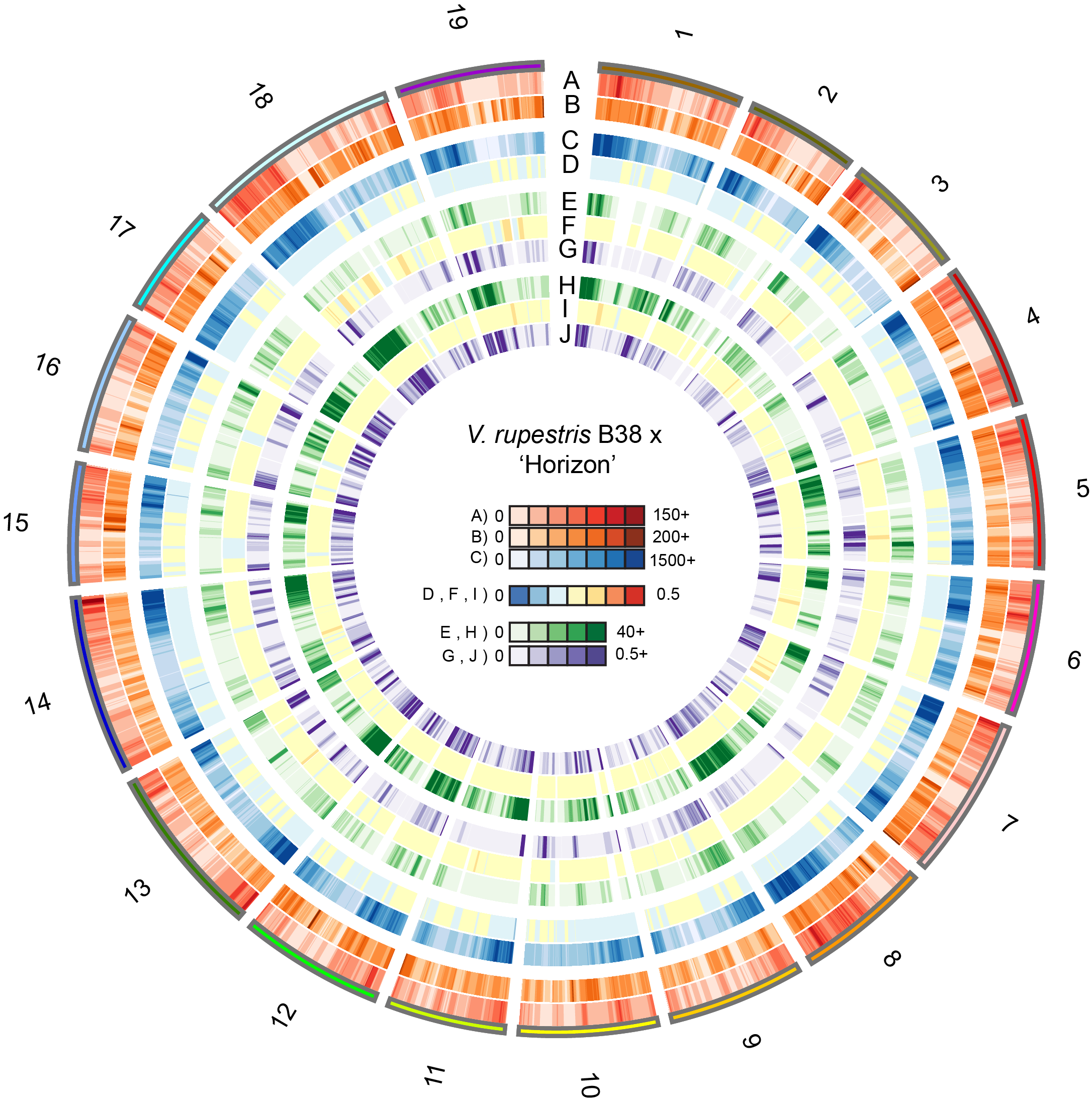

Supplement: S5 Fig — Data are shown on 1 Mb windows with a 100 Kb slide. A) Number of unique tags aligned, B) Mean tag depth calculated as total tag depth over number of unique tags aligned, C) Density of SNPs entering the HetMappS pipeline, D) Minor allele frequency (MAF) of SNPs entering the pipeline, E-J) SNP output from the synteny pipeline: E) SNP density ‘Horizon’, F) MAF ‘Horizon’ SNPs, G) Recombination frequency ‘Horizon’, calculated as the number of obligate crossovers per progeny per Mb, H) SNP density Illinois 547–1, I) MAF Illinois 547–1 SNPs, J) recombination frequency Illinois 547–1, calculated as the number of obligate crossovers per progeny per Mb. (TIF) [file pone.0134880.s005.tif]

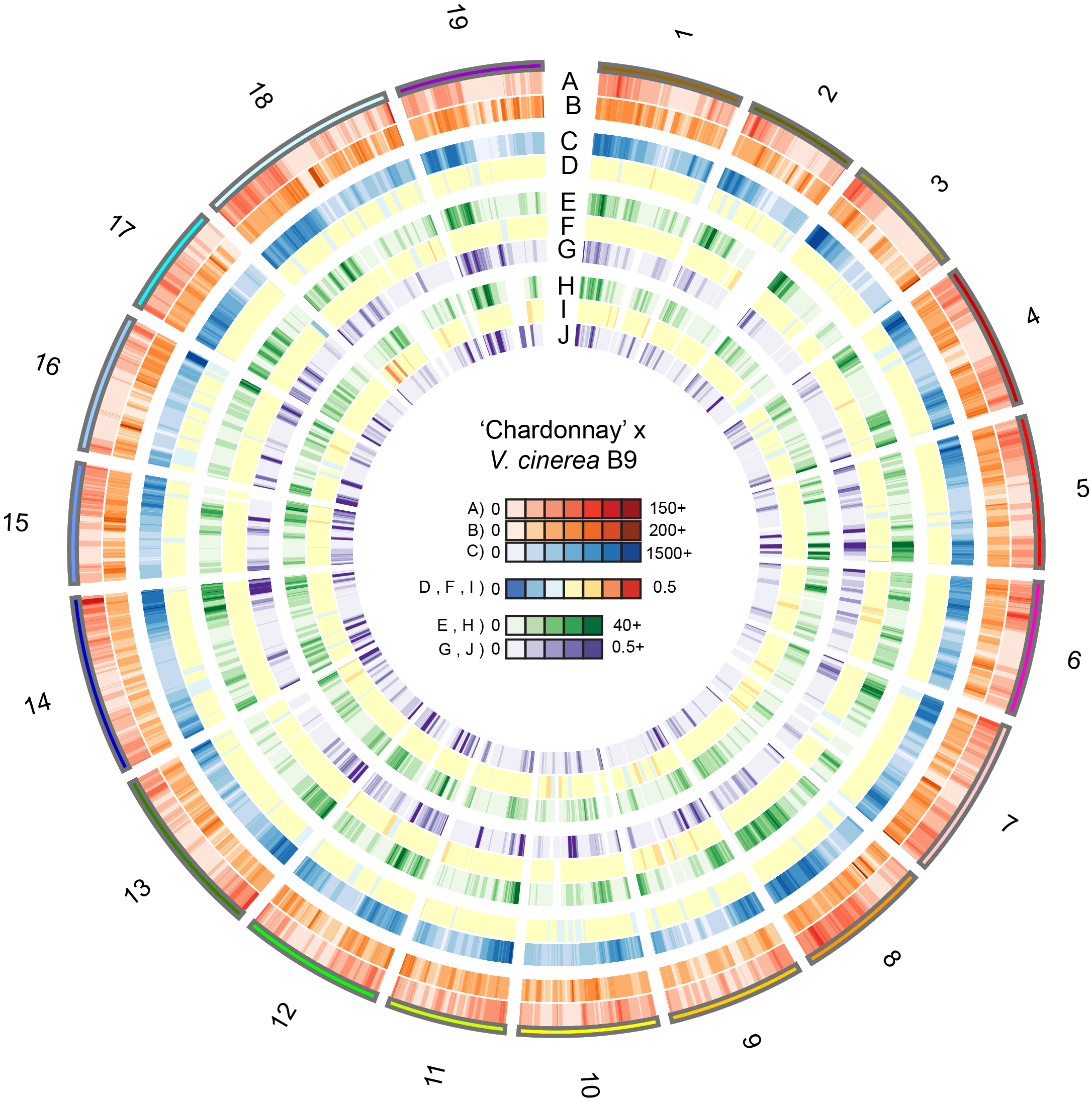

Supplement: S6 Fig — Data are shown on 1 Mb windows with a 100 Kb slide. A) Number of unique tags aligned, B) Mean tag depth calculated as total tag depth over number of unique tags aligned, C) Density of SNPs entering the HetMappS pipeline, D) Minor allele frequency (MAF) of SNPs entering the pipeline, E-J) SNP output from the synteny pipeline: E) SNP density ‘Horizon’, F) MAF ‘Horizon’ SNPs, G) Recombination frequency ‘Horizon’, calculated as the number of obligate crossovers per progeny per Mb, H) SNP density Illinois 547–1, I) MAF Illinois 547–1 SNPs, J) recombination frequency Illinois 547–1, calculated as the number of obligate crossovers per progeny per Mb. (TIF) [file pone.0134880.s006.tif]

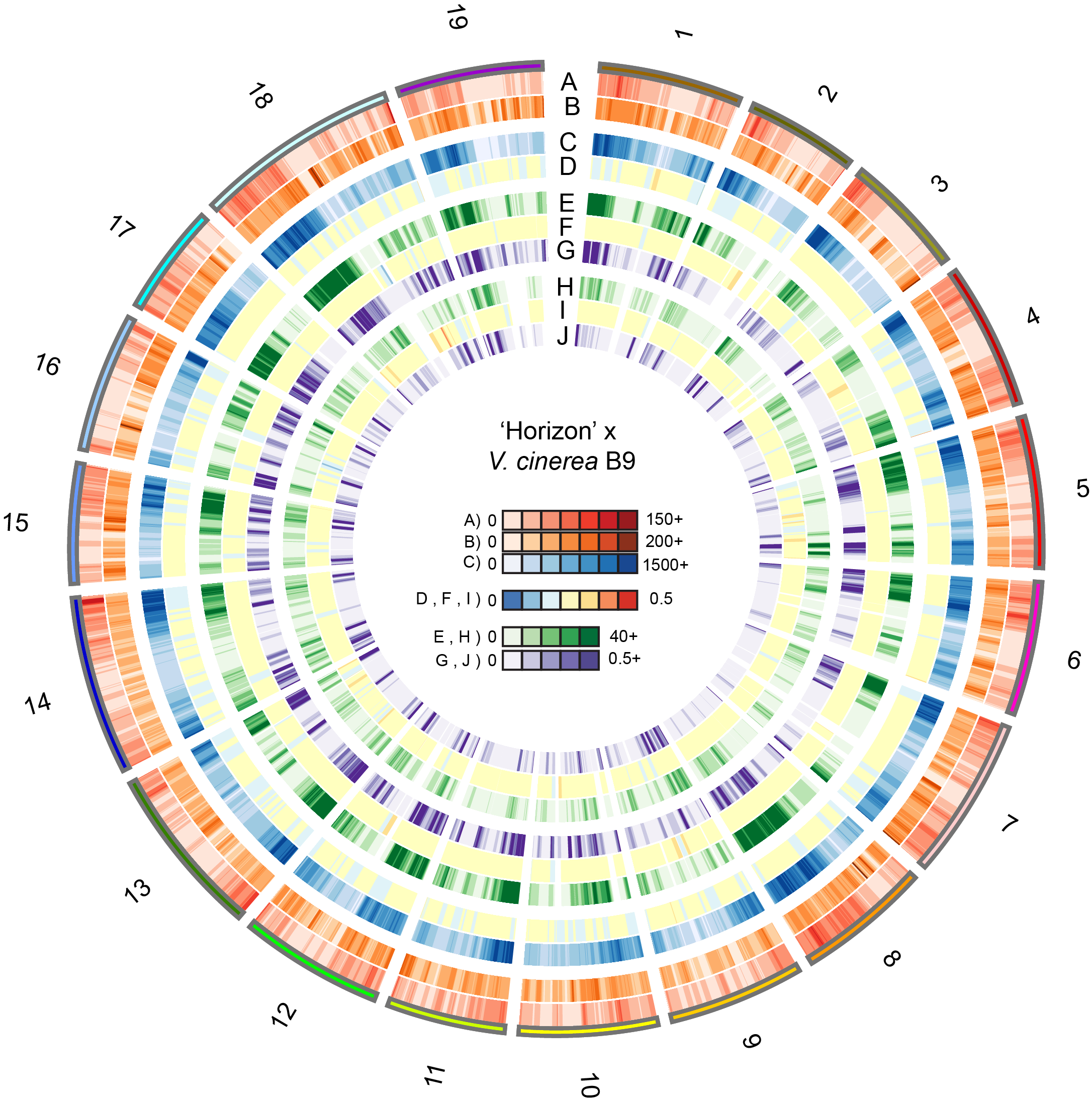

Supplement: S7 Fig — Data are shown on 1 Mb windows with a 100 Kb slide. A) Number of unique tags aligned, B) Mean tag depth calculated as total tag depth over number of unique tags aligned, C) Density of SNPs entering the HetMappS pipeline, D) Minor allele frequency (MAF) of SNPs entering the pipeline, E-J) SNP output from the synteny pipeline: E) SNP density ‘Horizon’, F) MAF ‘Horizon’ SNPs, G) Recombination frequency ‘Horizon’, calculated as the number of obligate crossovers per progeny per Mb, H) SNP density Illinois 547–1, I) MAF Illinois 547–1 SNPs, J) recombination frequency Illinois 547–1, calculated as the number of obligate crossovers per progeny per Mb. (TIF) [file pone.0134880.s007.tif]

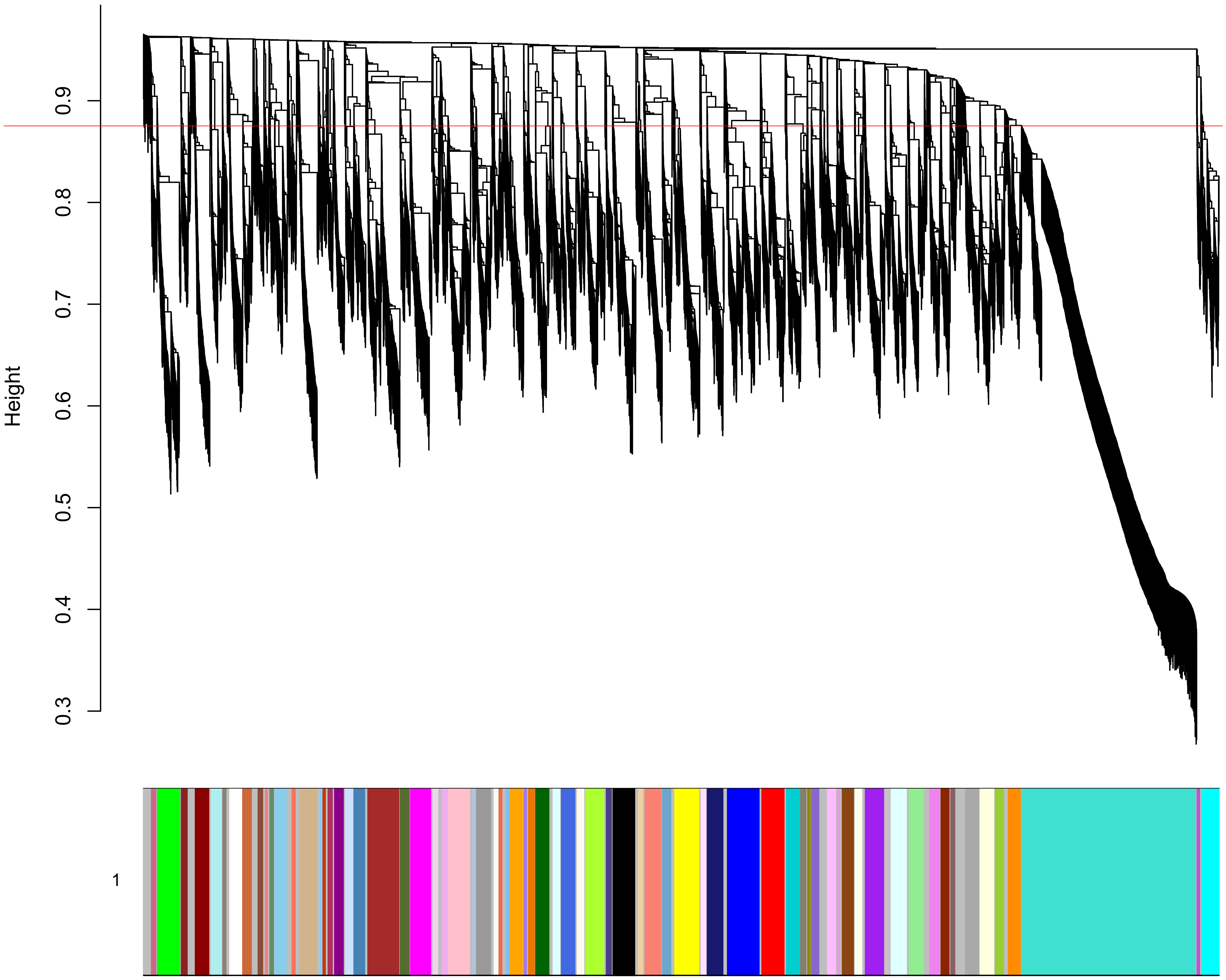

Supplement: S8 Fig — Dendrogram created from hierarchical clustering of topological overlap matrix for SNPs derived from whole genome amplified DNA of the V. rupestris B38 x ‘Chardonnay’ F1 family, displaying an atypical linkage pattern. This dendrogram was cut at 0.875 height and 5 linkage groups were discarded before proceeding to the phasing step. (TIFF) [file pone.0134880.s008.tiff]
