# Supplementary material for: Heterozygous Mapping Strategy (HetMappS) for High Resolution Genotyping-By-Sequencing Markers: A Case Study in Grapevine
Source: PLoS One. 2015 Aug 5;10(8):e0134880. doi: 10.1371/journal.pone.0134880 (PMC4526651; doi:10.1371/journal.pone.0134880)
Supplement: S1 File — (DOCX) [file pone.0134880.s009.docx]

HetMappS: Heterozygous Mapping Strategy pipeline documentation

The Heterozygous Mapping Strategy pipeline (HetMappS) consists of two separate phases: 1) genetic map creation, and 2) genetic map curation. The first part takes SNP data (from a VCF file), and creates linkage groups and then phases markers within each linkage group based on progeny segregation patterns and linkage. This pipeline is created specifically for use with large SNP datasets, as many markers are required for proper phasing. It has been optimized for and tested with genotyping-by-sequencing (GBS) data in *Vitis* spp. As the linkage group formation and phasing is dependent on genetic patterns, it is critical that the genotype data are high quality. Quality control and curation of the data prior to running the pipeline are required to obtain meaningful results. In particular, duplicated individuals should be checked for genetic identity prior to merging, and family outliers should be removed. Individuals with high proportions of missing data are removed in the pipeline, but these individuals can be discarded prior to running the pipeline.

Contents

[Installation / Dependencies / Running 2](#_Toc413231797)

[Genetic Map Creation Scripts 4](#_Toc413231798)

[Shared initial steps 6](#_Toc413231799)

[Synteny pipeline phasing 9](#_Toc413231800)

[Denovo pipeline phasing 12](#_Toc413231801)

[Ordering 14](#_Toc413231802)

[Genetic Map Curation Scripts 16](#_Toc413231803)

[Pipeline Overview 16](#_Toc413231804)

[Custom functions 18](#_Toc413231805)

## Installation / Dependencies / Running

This pipeline consists of a series of Perl and R scripts with several dependencies. All software is available for the Linux/Unix platform, and is also installed on BioHPC Computing Lab machines at Cornell University’s Bioinformatics Facility (<http://www.biotech.cornell.edu/brc/bioinformatics/biohpc-computing-laboratory>). For windows users, the installation of Cygwin, a collection of tools that provides functionality similar to Linux on windows, is recommended. The amount of memory required will depend on the size of your datasets, but we recommend at least 16 GB of RAM.

To run the pipeline, you will need to install:

- Perl (<http://www.perl.org/get.html>),
- R (<http://www.r-project.org/>)
- MSTMap (<http://alumni.cs.ucr.edu/~yonghui/mstmap.html>)
- The R following R packages:
  - WGCNA
  - R/qtl
  - snow
- The following Perl modules:
  - Statistics::R
  - Getopt::Long
  - Scalar::Util
  - Data::Dumper
  - Statistics::Descriptive
  - POSIX

Perl modules are easily installed from CPAN. For example, from the command line run the following to install Statistics::R. Repeat for each module that is required.

- perl –MCPAN –e ‘install Statistics::R’

R packages are easily installed from CRAN. To install WGCNA and its dependencies use the following command into the R console:

- source(“http://bioconductor.org/biocLite.R”)
- biocLite(“impute”)
- install.packages(“WGCNA”)

To install R/qtl, use the following command in the R console:

- install.packages(“qtl”)

To install snow package, use the following command in the R console:

- install.packages(“snow”)

See <http://labs.genetics.ucla.edu/horvath/CoexpressionNetwork/Rpackages/WGCNA/> and <http://www.rqtl.org/> for more information on WGCNA and R/qtl

To download and install MSTMap, download the source file source.tar.gz from the website, extract it, descend into the directory and compile with the following commands:

- tar –xvf source.tar.gz
- cd MSTMap
- make

Output is MSTMap.exe

Following installation, to run each portion of the pipeline, the proper script is invoked in the following manner. The directory containing the scripts must either be in the path, or the full path to the script must be specified.

Example command input 1 (script in current directory or path):

perl major_minor_VCF.pl -vcf infile.vcf -o outfile.vcf

System command

Pipeline script and options

Input (point to your file)

Output (creates a file or set of files with this name or base name)

Example command input (full path to script specified):

perl /home/scripts/HetMappS/major_minor_VCF.pl -vcf infile.vcf -o outfile.vcf

To generate a summary of available options for each script, simply run the script without any options:

perl major_minor_VCF.pl

## Genetic Map Creation Scripts

#### Pipeline Overview

This first portion of the pipeline takes SNP data as input, and by leveraging genetic linkage, completely phases markers based on progeny genotypes through first assigning markers to linkage groups, and then phasing within each linkage group. There are three separate pipelines that can be used: synteny, synteny with genetic ordering, and de-novo linkage group creation and genetic ordering. The synteny pipelines require that SNP positions in the input VCF file correspond to the reference genome being used for marker anchoring, and uses that positional information for linkage group creation. That is, markers within each chromosome are separated by parental contribution, creating two (or more) linkage groups for each chromosome. The denovo pipeline does not require a reference genome, but rather uses two rounds of clustering of progeny genotypes to first create linkage groups, and then phase them. If progenitor genotype data are available, they can be used for validation purposes and for assigning linkage groups to chromosomes and progenitors.

Both pipelines begin by identifying pseudotestcross markers (major_minor_VCF.pl and get_pseudotestcross_markers.pl), and then diverge. The synteny and synteny with genetic ordering pipelines follow the same procedure, with the optional addition of an ordering step after phasing (phased2mstmap.pl).


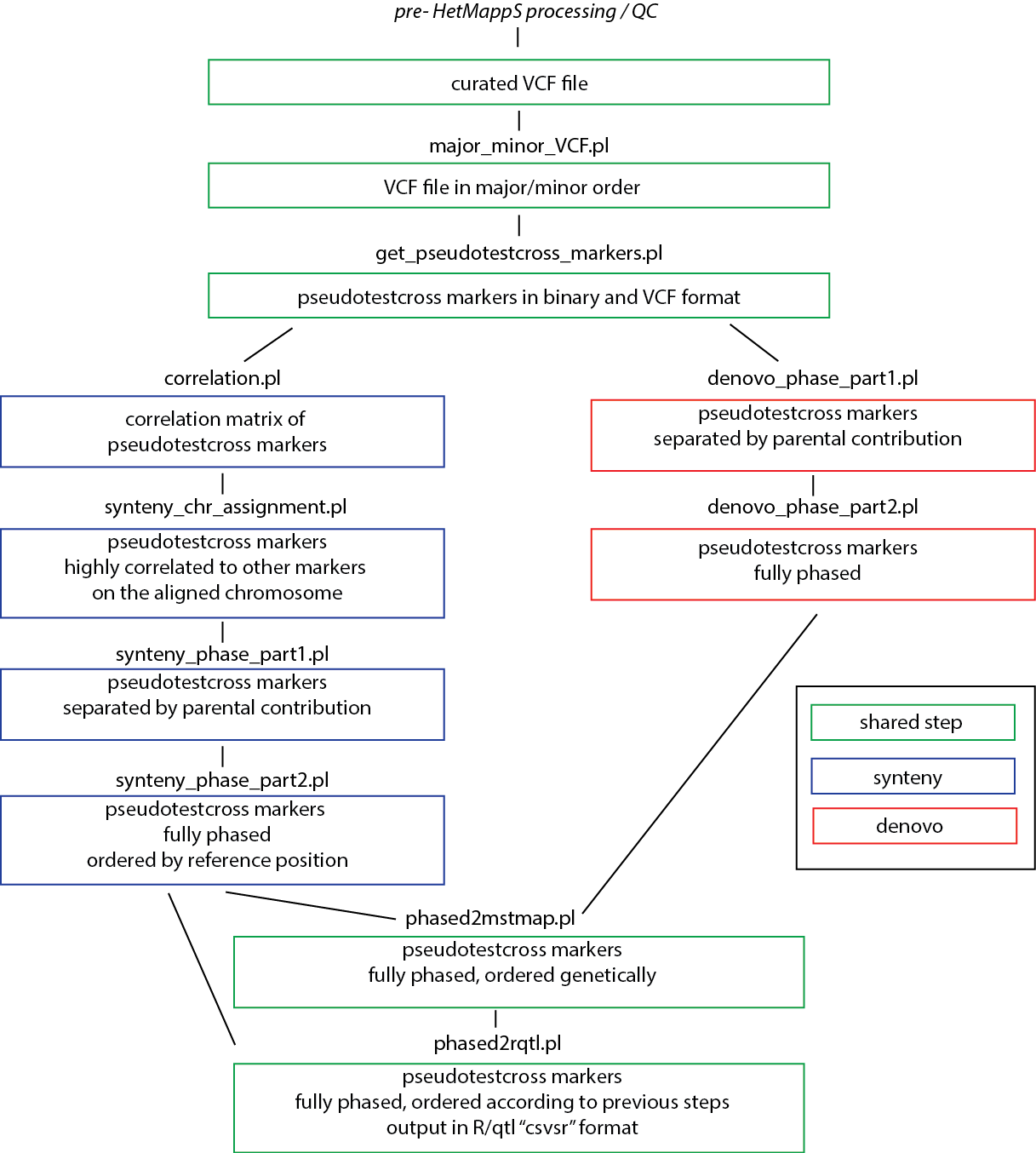


Figure 1. HetMapps Overview

### Shared initial steps

1. **major_minor_VCF.pl**

This script converts a VCF file from reference / alternative format to major / minor allele format, and also removes any sites that are monomorphic or have all missing data. Monomorphic (invariant) sites can result after slicing data from a larger dataset, and after removing or merging individuals.

- - Input: VCF file
  - Output: VCF file and log file
  - Options:

-vcf the input vcf file (required), with only true F1 progeny and progenitors

-o The base name of the output file. Defaults to inputfileprefix.majorminor.vcf

1. **get_pseudotestcross_markers.pl**

This script examines marker segregation in the progeny to identify pseudotestcross (Pt) markers, and outputs both a VCF file and a binary (coded 0=no minor allele, 1=minor allele present) file with the progeny genotypes. If progenitors are indicated in the option “indfile,” it also outputs separate VCF and binary files for the progenitors. The progenitor files can be used for downstream validation. Putative genotyping errors in the pseudotestcross markers are also identified and corrected, based on parameter choices (see diagram and description below).

- Input: VCF file and individual info file (optional)
- Output: VCF file and binary file for progeny, VCF file(s) and binary file(s) for progenitors (parents and /or grandparents, if specified in the individual info file), log file
- Options:

-vcf vcf (required) the vcf file, with only true progeny and progenitors, and in major/minor allele format

-i indfile (optional) the file indicating parents and grandparents. two tab delimited columns, first column has the id (must match vcf file exactly) second column has the designation (either parent or grandparent) all others are assumed to be progeny.

Note: if you create this file in windows and transfer it to a linux platform be sure to save in tab delimited format and use the command dos2unix <filename> after transferring.

-o outfilebase. The base name of output files

-error_maf. sequencing error threshold (optional, default = 0.05).

The maximum allele frequency an allele can be at to be considered a sequencing error

-tol tolerance (optional, default = 0.125). The tolerance away from the expected allele or genotype frequencies (one-sided) i.e. tolerance of .1 for AF of 0.5 would give a possible range of 0.4 to 0.6. Can't be set higher than .125

-geno (optional, default = 0.5) minimum genotyping rate (after error correction) to retain SNP

-minGQ (optional, default = 98) minimum genotype quality score to retain a homozygote,

also used for filtering progenitor genotypes

-maxerr (optional, default = 0.05) maximum proportion of errors to total genotypes, where errors are genotypes that are not AA, AB, or BB, or BB genotypes with GQ > minGQ

-v (optional, default is non-verbose) print out the result for each marker to the log file in addition to the summary

First, progeny and progenitor genotypes are separated.

Pseudotestcross markers are identified, filtered and corrected based on patterns in the progeny, using the following criteria (sequentially:

1. Alleles with minor allele frequencies less than the specified error_maf are converted to missing values.
2. SNPs with genotyping rate below the specified rate geno are removed.
3. SNPs that have more or less than 2 segregating alleles are removed.
4. SNPs with less than 2 segregating genotypes are removed.
5. SNPs not satisfying
   1. 0.75 + tol > freq(A) > 0.75 – tol and
   2. 0.25 + tol > freq(B) > 0.25 tol are removed,
6. Genotypes are filtered in the following manner:
   1. GQ(AA) < minGQ -> NA (masked)
   2. GQ(BB) > minGQ -> NA (error)
   3. GQ(BB) < minGQ -> AB (corrected)
7. Genotyping rate is re-calculated, and SNPs with genotyping rate below the specified rate geno are removed
8. Error rate (proportion of BB markers with GQ > minGQ) is calculated, and SNPs with error rate above the maxerr threshold are removed

Genotypes for progenitors for the putative pseudo-testcross markers that were identified based on progeny genotypes are extracted from the original VCF file. All progenitor genotypes with GQ < minGQ are converted to NA, and output into a separate file, in the same formats as progeny genotypes.


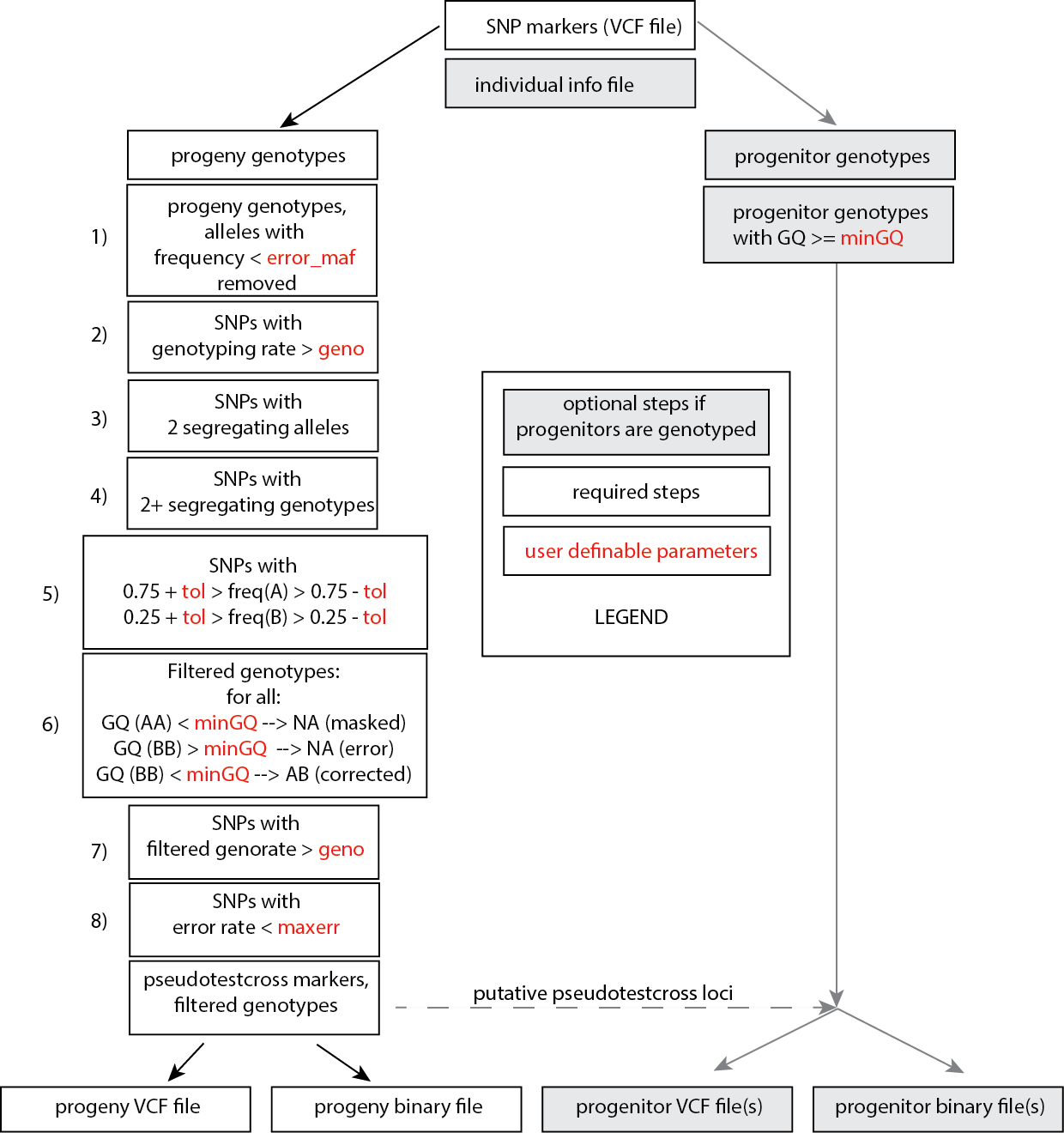


### Synteny pipeline phasing

1. **correlation.pl**

This script uses the binary encoded genotypes of the pseudotestcross markers to create a correlation (r^2^) matrix, which is used in the following step to test SNP chromosome assignments. For large files multiple pieces of the matrix can be calculated simultaneously, and stitched back together. Reduce the chunk size to reduce memory requirements if necessary.

- Input: progeny binary file (from get_pseudotestcross_markers.pl)
- Output: r^2^ matrix file, marker list for marker identification, log file
- Options:

-b binaryfile (required) the progeny binary file, the result of get_pseudotestcross_markers.pl

-t number of threads to use (defaults to 1)

-o output file base name (defaults to binaryfile name)

-d temporary directory to hold output (defaults to “temp”)

-s chunk size for processing markers (defaults to 5,000). Change depending on amount of RAM and threads available.

1. **synteny_chr_assignment.pl**

This script uses the correlation matrix to test chromosome assignment (SNP chromosome and position are read from the binary file, which is carried on from the original VCF files used to produce the binary file). Each SNP is correlated with all other SNPs according to presence/absence of the minor allele. The mean correlation between the SNP in question and SNPs on each of the chromosomes designated in the chromosome info file is calculated. The chromosome with the highest mean correlation is tested against the second highest mean correlation. If the highest mean correlation is at least diff times higher than the second mean correlation, and the highest mean correlation is to the chromosome to which the SNP is assigned in the binary file, the SNP is designated as ‘agree’ and is retained for output. If the highest mean correlation is at least diff times higher than the second mean correlation, and the highest mean correlation is not to the chromosome to which the SNP is assigned in the binary file, the SNP is designated as ‘disagree’ and is not retained. If the highest mean correlation is not at least diff times higher than the second mean correlation, the SNP is designated as ‘unresolved’ and is not retained.

- Input: correlation file and marker list from correlation.pl, binary file (from get_pseudotestcross_markers.pl), chromosome info file
- Output: binary file with assigned SNPs and a log file
- Options:

-c correlation file (required), output from correlation.pl

-m marker list (required), output from correlation.pl

-b binary file (required), input to correlation.pl

-chr chromosome info file, a file with the columns chromosome# (integer values only)

and length (only chromosomes you want to create linkage groups for) matching the reference genome used for SNP calling prior to the HetMappS pipeline. Make sure there are no blank lines at the bottom of the file.

-diff ratio cutoff for correlation (default=2) the number of times greater the mean correlation to the top ranking chromosome must be than the second ranking chromosome.

-o outfilebase (default = correlationfilename.diff[diff]).

-v verbose (print info about each marker to the logfile in addition to the summary

1. **synteny_phase_part1.pl**

This script uses the R package “WGCNA,” ^[[1]](#footnote-1)^ originally created for analysis of microarray data. First, an adjacency matrix is calculated from the binary encoded genotype data, from which a topological overlap matrix is calculated and used for hierarchical clustering. Linkage groups (corresponding to the parent contribution for each chromosome) are created by cutting of the resulting dendrogram into distinct groups of SNPs. Three different cutting methods are used. Typically the static cut should result in two distinct groups with high SNP retention. Changing the cutting parameters can resolve different groups. Each chromosome should be split into at least 2 groups (one for each parent), and is occasionally split into multiple groups, depending on linkage patterns. Dendrogram figures and heatmaps of the toplogical overlap matrix, with the resulting marker groups from the three different cutting methods, can be output and examined with the verbose option and used to choose appropriate cutting parameters. Clustering of individuals is also performed for each linkage group, and dendrograms are output to detect individual outliers. If progenitor genotypes are available (parents), the genotypes are used for validation and can also be used to create an LGmap for ordering (see phased2mstmap.pl and phased2rqtl.pl).

See HetMappS tutorials and WGCNA tutorials, especially <http://labs.genetics.ucla.edu/horvath/htdocs/CoexpressionNetwork/Rpackages/WGCNA/Tutorials/FemaleLiver-02-networkConstr-man.pdf> for more information.

- Input: correlation file and marker list from correlation.pl, binary file (from get_pseudotestcross_markers.pl), chromosome info file
- Output: a list of markers, separated into linkage groups (markers_by_parent.txt), a log file, optionally GENOvPARENT comparing known parental genotypes to inferred parental genotypes, and optionally figures of individual outliers, the topological overlap matrix, and the cut dendrogram.
- Options:

-b2 binary file (required), output from reference_based_chr_assignment.pl

-p parent binary file (optional); if available is used for validation; generated from get_pseudotestcross_markers.pl

-chr chromosome info file (required), a two column tab delimited file with the columns chromosome_number (integer values and only) and length. Only include chromosomes that you want to create linkage groups for.

-od output directory (required)

-t number of threads to use (defaults to 1)

-v verbose (print figures for hierarchical clustering / cutting of dendrograms, the topological overlap matrix for each chromosome, individual outlier detection plots for each chromosome. All can be used for manual curation)

-static_height (optional, defaults to 0.9)

-static_minsize (optional, defaults to 30)

-hybrid_height (optional, defaults to 0.998)

-hybrid_deepsplit (optional, defaults to 2)

-hybrid_pamrespectsdendro (optional, defaults to FALSE)

1. **synteny_phase_part2.pl**

This script uses the R package “WGCNA” ([Langfelder & Horvath, 2008](#_ENREF_1)) as above, to phase markers within each linkage group created with synteny_phase_part1.pl. It also implements a filter as described in synteny_chr_assignment.pl, using the “diff” parameter. See the “WGCNA” documentation and tutorials for more information on clustering and cutting parameters.

- Input: marker file from synteny_phase_part1.pl, binary file of HetMappS for progeny (output from synteny_chr_assignment.pl), chromosome info file, parent binary file (optional), grandparent binary file (optional).
- Output: a list of phased markers (phased.txt), a logfile, optionally GENOvPARENT.txt and/or GENOvGRANDPARENT.txt comparing known to inferred progenitor genotypes, and optionally figures of individual outliers, the toplogical overlap matrix, and the cut dendrogram.
- Options:

-l marker file (required, either the output from reference_based_phase_part1.pl or a custom file with linkage group information. If output from synteny_phase_parent2.pl one of –static, -dynamic, or –hybrid must be specified (defaults to –static).

If custom, option –custom must be in effect and the file must be tab delimited and contain the columns “ID” “CHR” “POS” and “PARENT”

-b2 binary file (required), output from reference_based_chr_assignment.pl

-p parent binary file (optional); if available is used for validation; generated from get_pseudotestcross_markers.pl

-g grandparent binary file (optional); if available is used for validation; generated from get_pseudotestcross_markers.pl

-chr chromosome info file (required), a two column tab delimited file with the columns chromosome_number (integer values and only) and length. Only include chromosomes for which you want to create linkage groups.

-od output directory (required)

-t number of threads to use (defaults to 1)

-v verbose (print figures for hierarchical clustering / cutting of dendrograms, the topological overlap matrix for each chromosome, individual outlier detection plots for each chromosome. All can be used for manual curation)

-static (must specify either –static, -dynamic, or –hybrid with option –m, indicating which cut method to use for parental assignment. Defaults to –static).

-dynamic (must specify either –static, -dynamic, or –hybrid with option –m, indicating which cut method to use for parental assignment. Defaults to –static).

-hybrid (must specify either –static, -dynamic, or –hybrid with option –m, indicating which cut method to use for parental assignment. Defaults to –static).

-custom (must specify either –static, -dynamic, or –hybrid with option –m, indicating which cut method to use for parental assignment. Defaults to –static).

-diff (optional, defaults to 2) the minimum value of the ratio of mean correlation of markers on the assigned linkage group to the mean correlation to markers on an alternate linkage group

-static_height (optional, defaults to 0.9)

-static_minsize (optional, defaults to 30)

-hybrid_height (optional, defaults to 0.998)

-hybrid_deepsplit (optional, defaults to 2)

-hybrid_pamrespectsdendro (optional, defaults to FALSE)

### Denovo pipeline phasing

1. **denovo_phase_part1.pl**

This script uses the R package “WGCNA,” ^[[2]](#footnote-2)^ originally created for analysis of microarray data. First, an adjacency matrix is calculated from the binary encoded genotype data, from which a topological overlap matrix is calculated and used for hierarchical clustering. Linkage groups are created by cutting of the resulting dendrogram into distinct groups of SNPs, using all pairwise combinations of the cut height and minimum size parameters specified. Different cutting parameters can resolve different groups. A filter is also applied using the diff parameter, to remove markers with low correlation to the assigned linkage group compared to any other linkage group. Each chromosome should be resolved into two or more linkage groups. If chromosome number is known, the cutting parameters that resolve twice the number of chromosomes or more should be tested. If linkage groups are not properly resolved, this can be detected in the map curation portion of the pipeline and different cutting parameters can be chosen for this step. Dendrogram figures, with the resulting marker groups from each different set of cutting parameters, can be output and examined with the verbose option, and can be used to choose appropriate cutting parameters. If progenitor genotypes are available (parents and/or grandparents), the genotypes are used for validation (GENOvPARENT.txt, CHRvLG.txt and CHRvLGtop.txt) and can also be used to create an LGmap for ordering (see phased2mstmap.pl and phased2rqtl.pl). denovo_phase_part2.pl

- Input: binary from get_pseudotestcross_markers.pl, parent binary file (optional)
- Outputs:
  - clustering of individuals to detect outliers (indOutlier.pdf); a summary of the number of linkage groups and markers for all cut height and minimum size combinations (lg_summary.txt);
  - Log file (log.txt); for each cut height and minimum linkage group size combination: a list of markers separated into linkage groups (hheight.ssize.LG.txt),
  - Dendrogram and cut results (hheight.ssize.hierTOM.pdf),
  - Table summarizing the markers from each chromosome (as designed by the binary file) against each linkage group (hheight.ssize.CHRvLG.txt),
  - Summary of the chromosome with the first and second highest number of markers contributing to each linkage group (hheight.ssize.CHRvLGtop.txt)
  - if progenitor genotypes are available, a file comparing known progenitor genotypes to inferred progenitor genotypes (hheight.ssize.GENOvPARENT.txt).
- Options:

-b binary file (required), output from get_pseudotestcross_markers.pl

-p parent binary file (optional); if available is used for validation; generated from get_pseudotestcross_markers.pl

-od output directory (required)

-t number of threads to use (defaults to 1)

-v verbose (print figures for hierarchical clustering / cutting of dendrograms and individual outlier detection plot for each chromosome. Each can be used for manual curation)

-diff (optional, defaults to 2) the minimum value of the ratio of mean correlation^2 of a marker to other markers on the same assigned linkage group to the mean correlation^2 of markers on an alternate linkage group

-height_min dendrogram cut height minimum to try (optional, defaults to 0.8)

-height_max dendrogram cut height maximum to try (optional, defaults to 0.9)

-height_step dendrogram cut height step size between min and max (optional, defaults to 0.0125)

-minsize_min dendrogram cutoff LG size minimum to try (optional, defaults to 50)

-minsize_max dendrogram cutoff LG size maximum to try (optional, defaults to 300)

-minsize_step dendrogram cutoff LG step size between min and max (optional, defaults to 50)

1. **denovo_phase_part2.pl**

This script uses the R package “WGCNA,” ^[[3]](#footnote-3)^ as above, but within each linkage group with three separate cutting algorithms to phase markers within each linkage group, for the cut height and minimum linkage group size combination selected from denovo_phase_part1.pl. Typically the static cut will properly separate into two phases. If progenitor genotypes are available (parents and/or grandparents), the genotypes are used for validation (GENOvPARENT.txt and GENOvGRANDPARENT.txt) and can also be used to create an LGmap for ordering (see phased2mstmap.pl and phased2rqtl.pl).

- Input: binary from get_pseudotestcross_markers.pl, LG marker file (output from denovo_phase_part1.pl), parent binary file (optional), grandparent binary file (optional)
- Output: a list of phased markers (phased.txt), a logfile, optionally GENOvPARENT.txt and / or GENOvGRANDPARENT.txt comparing known progenitor genotypes to inferred progenitor genotypes, and optionally figures of individual outliers, the toplogical overlap matrix, and phased markers against physical positions from the binary file.
- Options:

-b binary file (required), output from get_pseudotestcross_markers.pl

-l LG marker file (required, output from denovo_phase_part1.pl)

-p parent binary file (optional); if available is used for validation; generated from get_pseudotestcross_markers.pl

-g grandparent binary file (optional); if available is used for validation; generated from get_pseudotestcross_markers.pl

-od output directory (required)

-t number of threads to use (defaults to 1)

-v verbose (print figures for hierarchical clustering / cutting of dendrograms and individual outlier detection plot for each chromosome. Each can be used for manual curation)

-static_height (optional, defaults to 0.9)

-static_minsize (optional, defaults to 10)

-hybrid_height (optional, defaults to 0.998)

-hybrid_deepsplit (optional, defaults to 2)

-hybrid_pamrespectsdendro (optional, defaults to FALSE)

### Ordering

1. **phased2mstmap.pl**

This script uses output from either the synteny or denovo pipeline and executes ordering with MSTmap ^[[4]](#footnote-4)^, with optional iterative filtering on the maximum proportion of individuals with double crossovers per marker, dropping of suspicious genotypes, and dropping duplicate (redundant) markers. The MSTMap filters on nomapsize and too many missing genotypes are applied in iteration N (including N=0). Double crossover, duplicate marker and genotype filters identified in iteration N are applied in iteration N+1 (when r > 0). Options –cp, -md, -ms, -mt, and –of care MSTMap options. Please refere to MSTMap documentation for more information.

- Input: binary file (required, output from get_pseudotestcross_markers.pl in *de novo* pipeline or synteny_chr_assigment.pl in synteny pipeline), linkage group file (phased.txt, required, output from either synteny or denovo phase2), LGmap (optional)
- Output: MSTMap output files (LG.iteration.unordered, LG.iteration.ordered, LG.iteration.ordered.mstmap and LG.iteration.log), lists of markers and genotypes to drop (LG.iteration.ordered.markers2drop and LG.iteration.ordered.genotypes2drop), list of filter results (LG.ordered.filter), the combined map results from each iteration (all.iteration.mstmap, all.iteration.markers2drop and all.iteration.genotypes2drop). Markers and genotypes to drop from iteration N are implemented in iteration N+1 for ordering.
- Options:

-x mstmap executable location (required if it is not in path)

-b binary file (required, output from synteny_chr_assignment.pl for synteny pipeline or output from get_pseudotestcross_markers.pl for *de novo* pipeline)

-od output directory

-l linkage group / marker file (phased.txt)

-m LGmap (mapping of LG / phase onto some meaningful names, optional)

LGmap has 5 tab delimited columns: LG, PHASE, LABEL, PARENT_NUM, GRANDPARENT_NUM. LG and PHASE must match the linkage group file, LABEL is the label that you would like to propogate down the pipeline. If you want to join linkage groups this is the place to do it, prior to ordering you can use the information from the GENOvPARENT and GENOvGRANDPARENT files from the phase2 folder to create the LGmap. PARENT_NUM must be 1 or 2 (up to 2 parents per LG - expect 1 per LG). GRANDPARENT_NUM must be 1 or 2 (up to 2 grandparents per LG, expect one per LG/PHASE)

-r default=1 number of times to do iterative dropping and re-ordering,

suspicious genotypes reported by MSTMap and duplicate markers are dropped, and -xo filter is applied

-df distance_function (kosambi or haldane) default = kosambi

-cp cut_off_p_value default=2

-md no_map_dist default=15

-ms no_map_size default=5

-mt missing_threshold default = 0.5

-of objective_function default = ML

-xo markers creating double crossovers for greater than this proportion of individuals will be filtered (ie 0 allows no double crossovers, 1 allows all)

(implemented when the r parameter is > 0, default = 0.2)

1. **phased2rqtl.pl**

This script takes output from either the synteny pipeline or the denovo pipeline and converts to R/qtl’s “csvsr” format, for use with the manual curation portion of the pipeline and all available R/qtl functions.

- Input: binary file (required, output from get_pseudotestcross_markers.pl), linkage group / marker file (phased.txt, required, output from either synteny or denovo phase2), LGmap (optional), order file (optional, could be output from phased2mstmap.pl), markers to drop (optional, could be output from phased2mstmap.pl), genotypes to drop (optional, could be output from phased2mstmap.pl)
- Output: geno and pheno files for R/qtl, in “csvsr” format (outfilebase.geno and outfilebase.pheno), and a log file (outfilebase.log)
- Options:

-b binaryfile, required

-l linkage group / marker file (phased.txt), required

-c cross type (“BC” or “4way”), required

-m LGmap (mapping of LG / phase onto some meaningful names, optional)

LGmap has 5 tab delimited columns: LG, PHASE, LABEL, PARENT_NUM, GRANDPARENT_NUM.

LG and PHASE must match the linkage group file.

LABEL is the label that you would like to propagate down the pipeline.

If you want to join linkage groups this is the place to do it, prior to ordering.

You can use the information from the GENOvPARENT and GENOvGRANDPARENT files to create the LGmap.

PARENT_NUM must be 1 or 2 (up to 2 parents per LG - expect 1 per LG).

GRANDPARENT_NUM must be 1 or 2 (up to 2 grandparents per LG, expect one per LG PHASE).

-order new order, tab delimited file with ID, LG, POS (optional, can use all.n.mstmap from phased2mstmap.pl)

-drop_markers markers to drop, file with one marker name per line (optional). If the iterative ordering option in phased2mstmap was used, if iteration N is chosen, use the output all.[N-1].markers2drop to drop the same markers that were dropped for ordering in iteration N.

-drop_genotypes genotypes to drop, file with one marker name per line (optional). If the iterative ordering option in phased2mstmap was used, if iteration N is chosen, use the output all. [N-1].genotypes2drop to drop the same genotypes that were dropped for ordering in iteration N.

-o outfilebase (defaults to binaryfiel.rqtl) output files are in the format “csvsr” ‘for R/qtl

-v verbose (print detailed output)

## Genetic Map Curation Scripts

This portion of the pipeline allows curation of genetic maps created in the previous steps, taking advantage of the functions and interactive interface of R/qtl^[[5]](#footnote-5)^. A detailed explanation of the principles and theory behind creation and curation of genetic maps can be found in R/qtl book and webpage^6^. In HetMappS genetic maps, we identified two main causes of problems that artificially inflated map size: Incorrect marker order and inclusion of spurious markers. We developed commands and functions to address these issues, and organize them in five scripts that require user’s input. Since each project may present different challenges, this portion of the pipeline was designed to be flexible. Here we provide tools: a) to evaluate whether the initial ordering is correct by observing recombination fraction (rf) / LOD plots, and b) to automatically remove spurious markers that inflate the genetic distances within each linkage group. In order to simplify this analysis we have automated filtering and calculation steps, with manual curation being necessary at the end of the pipeline. In order to make full use of R/qtl functionalities, genetic map curation is performed using the backcross format (BC).

### Pipeline Overview

This pipeline for map curation addresses two main issues. First, problems in the initial marker order – when markers are more correlated with non-adjacent markers – and second, when spurious markers make LGs size larger than expected (100 cM assuming 0 to 2 crossovers per chromosome, per meiosis in a F1 from heterozygous parents). In this portion of the pipeline we use several functions from R/qtl, or HetMappS functions derived from R/qtl functions, to aid the decision making process described in the following figure (3).


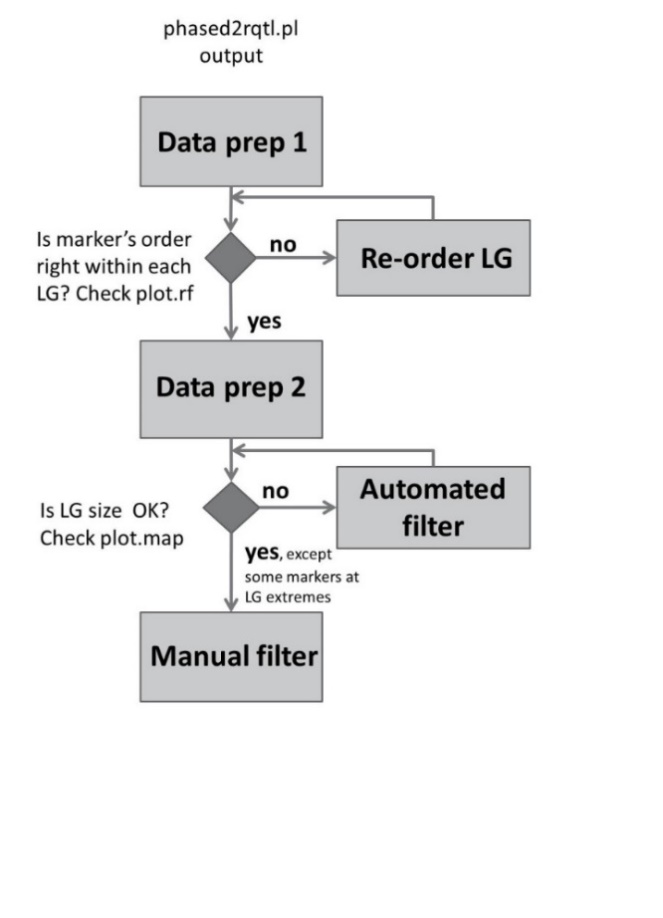


**1**

**2**

**3**

**4**

**5**

**Figure 3. Genetic map curation is a decision process**. Our pipeline provide scripts (boxes 1 to 5) to process genotype data and aid in answering the questions (diamonds) of the genetic map curation process.

Box 1: Data prep 1 script prepares the data for the first decision step. Here, spurious markers and individuals are removed according to missing data percentage or proportion of double crossovers (HetMappS R /qtl function ‘PrepCross’) and rf/LOD plots are generated (HetMappS R/qtl function ‘rfSummary’).

Question 1: Is marker’s order right within each LG? In this stage, rf/LOD plots are visually inspected by the user. Two outputs are possible; either all linkage groups (LG) show the expected correlation among markers (B) or some LG have markers that are more correlated with non-adjacent markers (A). An example of rf/LOD plots for an LG in the wrong and right order is showed below (Figure 4).


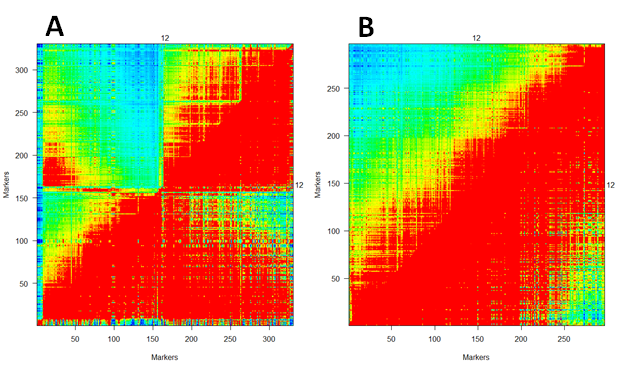


**Markers**

**Markers**

**Markers**

**Markers**

**Figure 4. Is marker’s order right within each LG?** Pairwise recombination fraction (rf, upper left triangle) and LOD score (LOD, lower right triangle) of (A) raw MSTmap output and (B) curated output for linkage group 12 of ‘Horizon’ x Illinois 547-1 *de novo* map. Maps obtained from MSTmap were inspected for non-optimal order, observed as groups of markers with lower recombination fractions (red) and higher LOD scores (red) with non-neighboring markers. The curated output seen in (B) was obtained after dropping 34 markers (10.3% of original markers) and re-ordering with orderMarkers function in R/qtl. Plots were generated using the est.rf and plot.rf functions from R/qtl .

**Markers**

**Markers**

Box 2: Re-order LG script provides commands and functions to re-order LG identified by the user. First, suspicious markers can be dropped manually using the r/QTL function ‘drop.markers’ and then markers within specific LGs can be re-ordered using R/qtl functions ‘switch.order’ or ‘orderMarkers’. rf/LOD plots are generated for visual inspection of the resulting maps.

Box 3: Data prep 2 script recalculates genetic distances and performs calculations for the automated filter. Steps involved are: 1) Determine the specific error rate for this cross by using the HetMappS R/qtl function ‘ErrorRate’, 2) Re-estimate genetic distances using the HetMappS R/qtl function ‘FlipLG’, which also allows to invert the order of LGs defined by the user, and 3) calculate the changes in LOD and map distance due to dropping one marker at the time, with either the R/qtl function ‘droponemarker’ or the HetMappS R/qtl function ‘SlideDroponemarker’. This last step can be slow, as the genetic distance of the LG needs to be calculated after dropping each marker. Check the R/qtl manual for a description of ‘droponemarker’ function, or check below for a description of the HetMappS R/qtl function ‘SlideDroponemarker’.

Question 2: Is LG size OK? In a F_1_ from heterozygous parents, the average expected size for LG is around 100 cM assuming 0 to 2 crossovers per chromosome, per meiosis. The user needs to determine the next step based on the LG size. If LGs lengths are unusually large, the automated filtering script (box 4) can help to select and drop suspicious markers faster, before proceeding with the manual filtering step (box 5). If LGs are of the expected size with the exception some outlier markers (usually located at the extreme of the LG), the user can proceed directly to the manual filtering step.

Box 4: Auto-filter script drops markers that likely do not belong to the assigned LG, by selecting markers that will have a higher impact in the log likelihood or map distance when removed. This script uses the output from either ‘droponemarker’ or ‘SlideDroponemarker’ from the previous step (Data prep 2). Here, the user needs to determine a threshold for the LOD change and Ldiff for removing markers with the HetMappS R/qtl function ‘FilterByLODSlidingDroponemarker’.

Box 5: Manual filter script creates for each marker a summary of the mean recombination fraction and the mean LOD score (HetMappS R/qtl function ‘rfSummary’), to aid the identification of spurious markers. Linkage groups are manually inspected by the user for suspicious markers, including those creating large gaps or with high mean recombination fraction or low mean LOD compared to their neighboring. Suspicious markers should be removed manually. After filtering markers, genotyping error rate and genetic distances are re-calculated.

### Custom functions

New functions used in the process described above were created based on existing R/qtl functionality and are centralized in script HetMappS_functions.R. In order to use these functions, they need to be loaded using the R command source( ).

**1) PrepCross** **(used in HetMappS_Data_Prep_1.R)**

This function performs standard preparation of cross data for linkage map analysis, according to http://www.rqtl.org/tutorials/geneticmaps.pdf

- Input: cross object.
- Output: cross object with markers and individuals removed, according to options below.
- Options:

k.prop.markers.typed: The desired minimum proportion of information for a marker to be retained, with values between 0 (all markers are retained) and 1 (only markers with complete information for all individuals are retained). Default = 0.6

k.prop.individuals.typed: The desired minimum proportion of information on each individual to be retained, with values between 0 (all individuals are retained) and 1 (only individuals with complete information for all markers are retained). Default = 0.6

k.prop.mean.cross.over: For each individual, the maximum proportion of crossovers with respect to sample mean to be retained. Default = 2 (individuals with twice or more crossovers than the mean crossover value are discarded)

- Usage example:

> data <- read.cross(format = "csvsr", phefile = pheno.file, genfile = geno.file ,genotypes = c("A", "B"))

--Read the following data:

214 individuals

7018 markers

1 phenotypes

> data1 <- PrepCross(data,prop.markers.typed = 0.6, prop.individuals.typed = 0.6, prop.mean.cross.over = 2)

> summary(data1)No. individuals: 210

No. phenotypes: 1

Total markers: 6186

**2) FlipLG (used in HetMappS_Data_Prep_1.R)**

Invert the order of designated LGs and re-estimate the genetic distances for the whole cross object. If no LG are specified it will just calculate genetic distances according to input parameters.

- Input: cross object
- Output: cross object with desired LGs inverted and genetic distances re calculated
- Options:

lg2flip: vector containing the name of LG to invert

error.rate: Genotyping error rate of cross object. Default = 0.01

cluster: number of clusters to run parallel calculations. Default = 1

m.function: Mapping function to calculate cM from recombination fraction. Options are "haldane","kosambi","c-f","morgan". Default is Haldane.

- Usage example:

data2 <- FlipLG(data1,lg.flip = c(1,4,19), error.rate = 0.01)

**3) ErrorRate (used in HetMappS_Data_Prep_2.R, HetMappS_auto_filter.R, HetMappS_manual_filter.R)**

Estimate the LOD of a cross object at a range of error rates. The error rate for a given cross object is the value that maximizes the LOD estimate. Method according to http://www.rqtl.org/tutorials/geneticmaps.pdf, page 35, accessed April 2014

- Input: cross object
- Output: Table with error rates and their corresponding lod values, and a pdf file with these values plotted.
- Options:

cluster: number of clusters to run parallel calculations. Default = 1

err.end: Maximum error rate value used for this estimation. Default = 0.02

err.increment: Size of the increment on the error rate. Default = 0.0025

- Usage example:

error.table <- ErrorRate(cross.file,err.end = 0.03)

geno.error <- error.table[which.max(error.table [,'lod']),'Error_rate']

**4) SlideDroponemarker (used in HetMappS_Data_prep_2.R)**

Droponemarker is a useful R/qtl function to identify problematic markers, but it can be slow for large LGs. SlideDroponemarker runs the Droponemarker function in a sliding window within each LG, reducing the time to complete the calculations for larger maps.

- Input: cross object
- Output: A table similar to Dropeonemarker table output, but with two markers at each LG extreme removed and without “scanone” class. According to qtl package description, Dropeonemarker “gives a data frame…with each row being a marker. The ﬁrst two columns are the chromosome ID and position. The third column is a LOD score comparing the hypothesis that the marker is not linked to the hypothesis that it belongs at that position”. The fourth column is the difference in length when the marker is removed from the map.
- Options:

error.rate: Genotyping error rate of cross object, default = 0.01

window.size: Effective size of the sliding window. For each LG, the cross object is subset to window.size + 4 markers, Droponemarker function is applied, and two markers at each extreme of the window are trimmed from the report. The window moves along the LG until all markers are analyzed. Default = 5.

- Usage example:

>drop1marker.table <- SlideDroponemarker(data2, error.rate = 0.01, window.size = 5)

**5) FilterByLODSlidingDroponemarker (used in HetMappS_Auto_filter.R)**

Uses output from Droponemarker or SlideDroponemarker functions to filter out markers from a given cross object. Markers with both LOD and Ldiff values above the thresholds defined by user are dropped. LOD threshold can be either i) uniform: indicate one value for limLOD that will apply to all markers (no chr.list needs to be defined) or ii) specific for each chr/LG: a vector with chromosome names (chr.list) and a vector with LOD threshold (limLOD) should be given. This function needs to be used carefully, as markers located near the end of the chromosomes usually have higher LOD scores. Check Droponemarker description in R/qtl user manual.

- Input: A cross object to be filtered and a droponemarker or SlideDroponemarker object, where each row is a marker and the first two columns are the chromosome ID and position, the third column the LOD score and fourth column the difference in length when the marker is removed from the map.
- Output: The cross object with markers filtered according to given options
- Options:

lim.LOD: LOD threshold, SNPS with values over this threshold and limLdiff over the defined threshold (see next option) are droped. lim.LOD can be either a single value for all LG, or a vector with different values for each LG.

limLdiff: Ldiff threshold, used to drop SNPs with values over this threshold.

chr.list: a vector with chromosome/LG names, matching the lim.LOD values. If a uniform threshold is applied to all LG, there is no need to use this option.

- Usage example:

> data2.filter <- FilterByLODSlidingDroponemarker(data2, drop1marker.table, limLOD = -20, limLdiff = 2)

**6) rfSummary (Used in HetMappS_re-order.R, HetMappS_auto_filter.R, HetMappS_manual_filter.R)**

Creates a summary from cross objects with recombination fraction information (run est.rf first). Each row is a marker, first and second columns are chr/LG and position, third and fourth columns are distance to previous and distance to next marker, and fifth and sixth columns are average recombination fraction and average LOD respectively.

- Input: cross object
- Output: a table where each row is a marker, first and second columns are chr/LG and position, third and fourth columns are distance to previous and distance to next marker, and fifth and sixth columns are average recombination fraction and average LOD respectively.
- Usage example:

> data.manual.summary <- rfSummary(data.man)

#summary for chromosome 5

> data.manual.summary[data.manual.summary$chr == 5 , ]

1. Peter Langfelder and Steve Horvath, “WGCNA: An R Package for Weighted Correlation Network Analysis,” *BMC Bioinformatics* 9, no. 1 (December 29, 2008): 559, doi:10.1186/1471-2105-9-559. [↑](#footnote-ref-1)
2. Ibid. [↑](#footnote-ref-2)
3. Yonghui Wu et al., “Efficient and Accurate Construction of Genetic Linkage Maps from the Minimum Spanning Tree of a Graph,” *PLoS Genetics* 4, no. 10 (October 2008), doi:10.1371/journal.pgen.1000212. [↑](#footnote-ref-3)
4. Yonghui Wu et al., “Efficient and Accurate Construction of Genetic Linkage Maps from the Minimum Spanning Tree of a Graph,” *PLoS Genetics* 4, no. 10 (October 2008), doi:10.1371/journal.pgen.1000212. [↑](#footnote-ref-4)
5. Karl W Broman et al., “R/qtl: QTL Mapping in Experimental Crosses,” *Bioinformatics (Oxford, England)* 19, no. 7 (May 1, 2003): 889–90.

   ^6^ Karl W. Broman and Saunak Sen, *A Guide to QTL Mapping with R/qtl* (Springer Science & Business Media, 2009); Karl W. Broman, “Genetic map construction with R/qtl”, http://www.rqtl.org/tutorials/geneticmaps.pdf [↑](#footnote-ref-5)
