# Supplementary material for: Heterozygous Mapping Strategy (HetMappS) for High Resolution Genotyping-By-Sequencing Markers: A Case Study in Grapevine
Source: PLoS One. 2015 Aug 5;10(8):e0134880. doi: 10.1371/journal.pone.0134880 (PMC4526651; doi:10.1371/journal.pone.0134880)
Supplement: S4 File — (PDF) [file pone.0134880.s012.pdf]

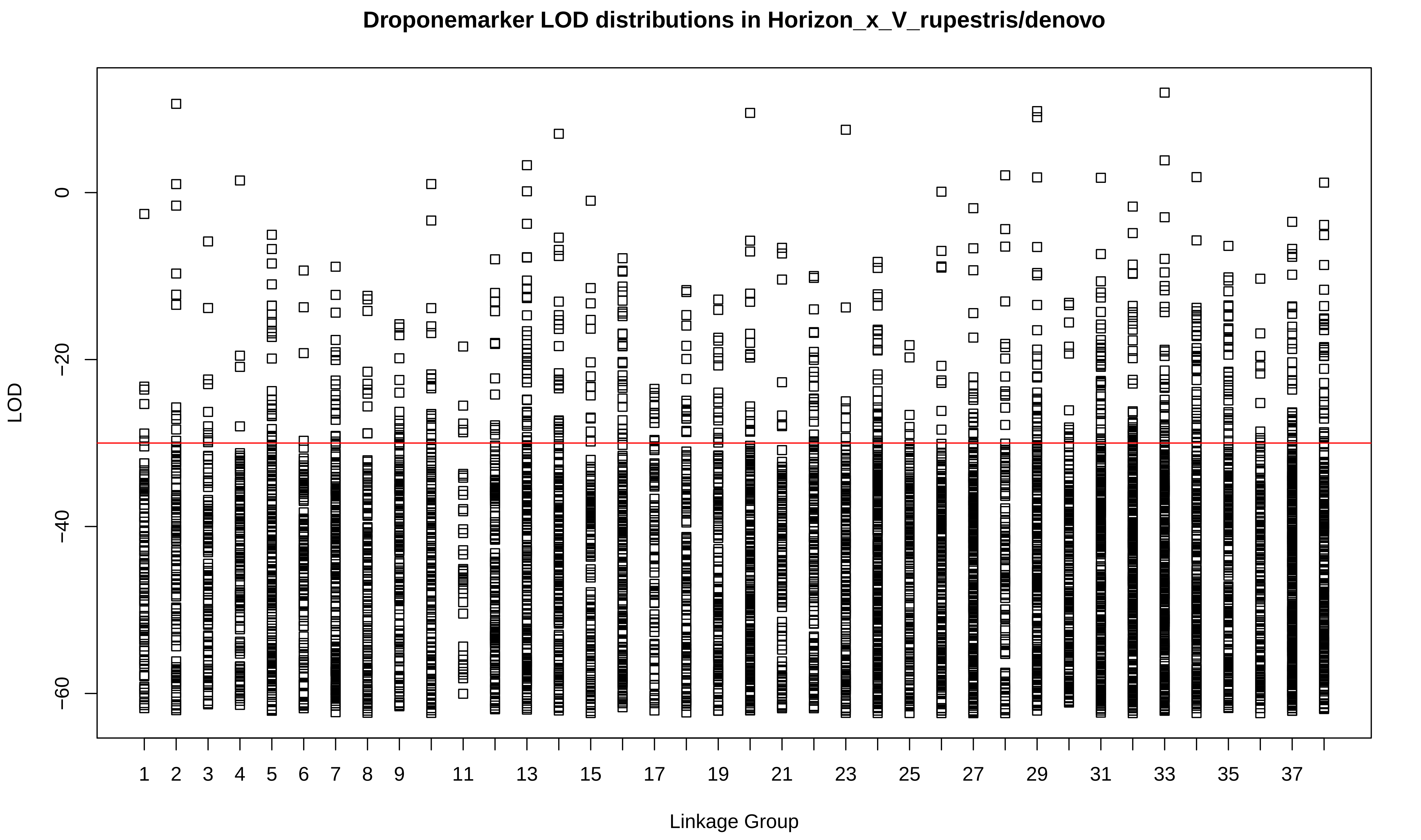

Droponemarker LOD distributions in Horizon_x_V_rupestris/denovo
LOD
0
−20
−40
−60
1
2
3
4
5
6
7
8
9
11
13
15
17
19
21
23
25
27
29
31
33
35
37
Linkage Group

Droponemarker LOD distributions in Horizon_x_V_rupestris/synteny

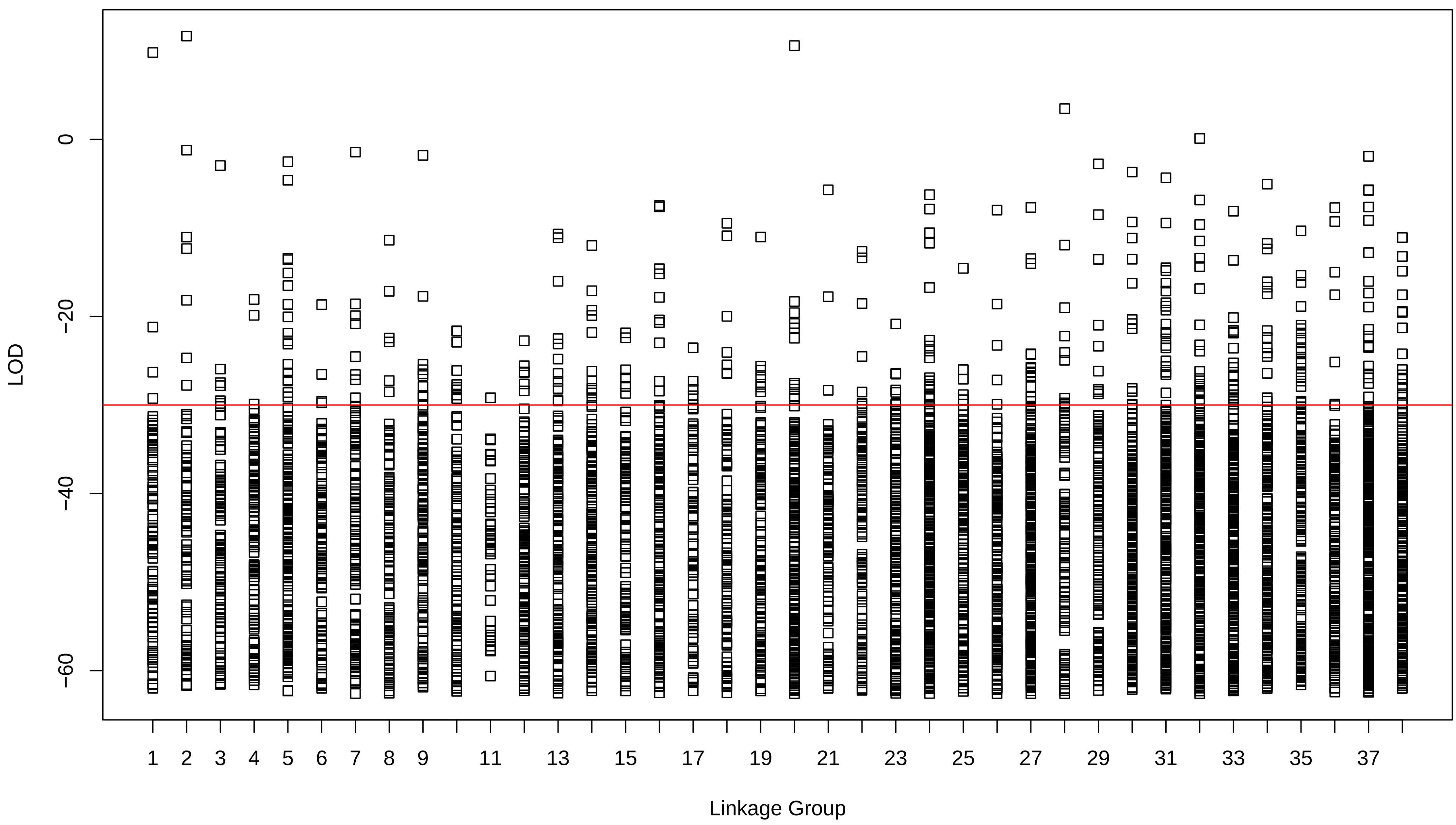

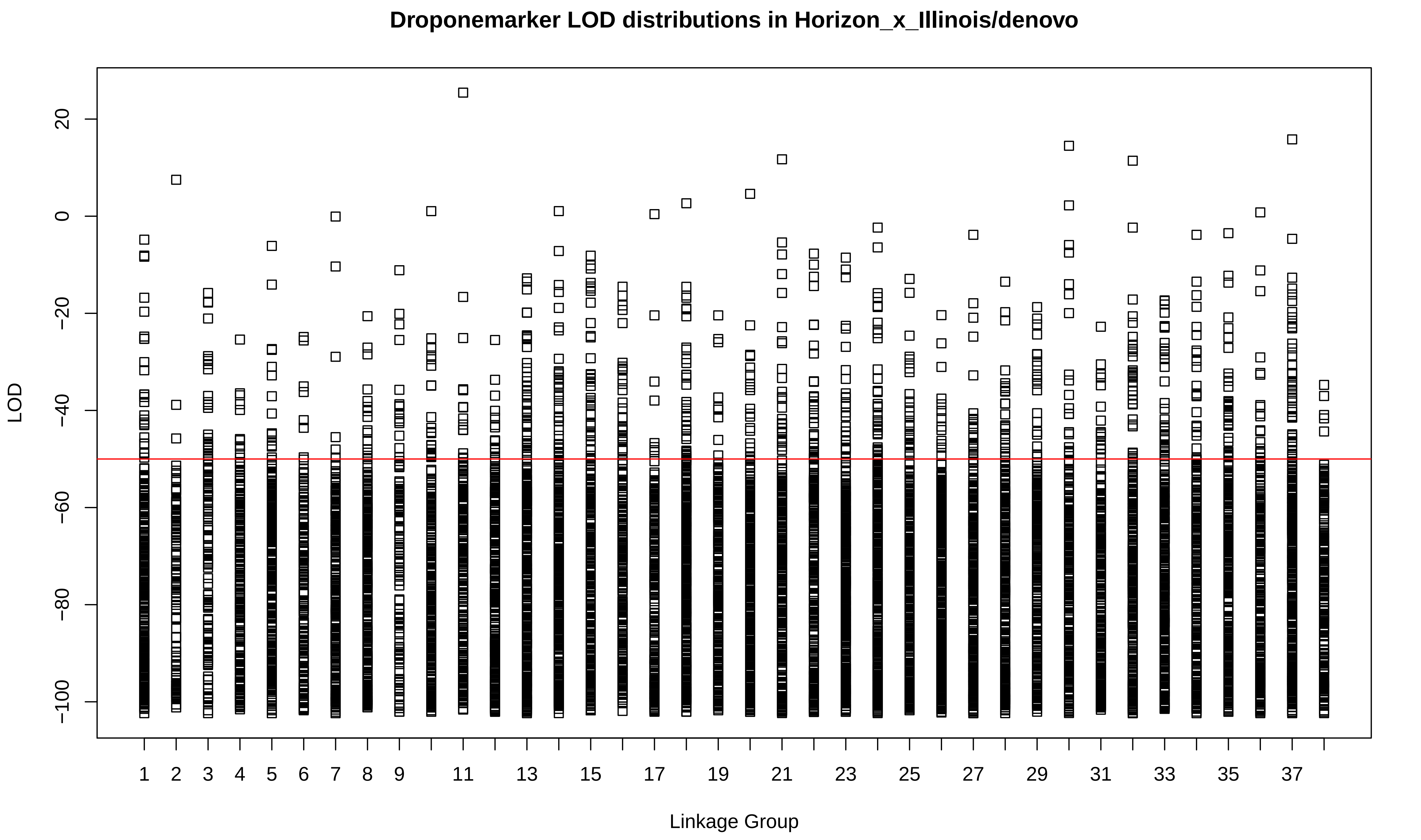

Droponemarker LOD distributions in Horizon_x_Illinois/denovo
LOD
20
0
−20
−40
−60
−80
−100
1 2 3 4 5 6 7 8 9 11 13 15 17 19 21 23 25 27 29 31 33 35 37
Linkage Group

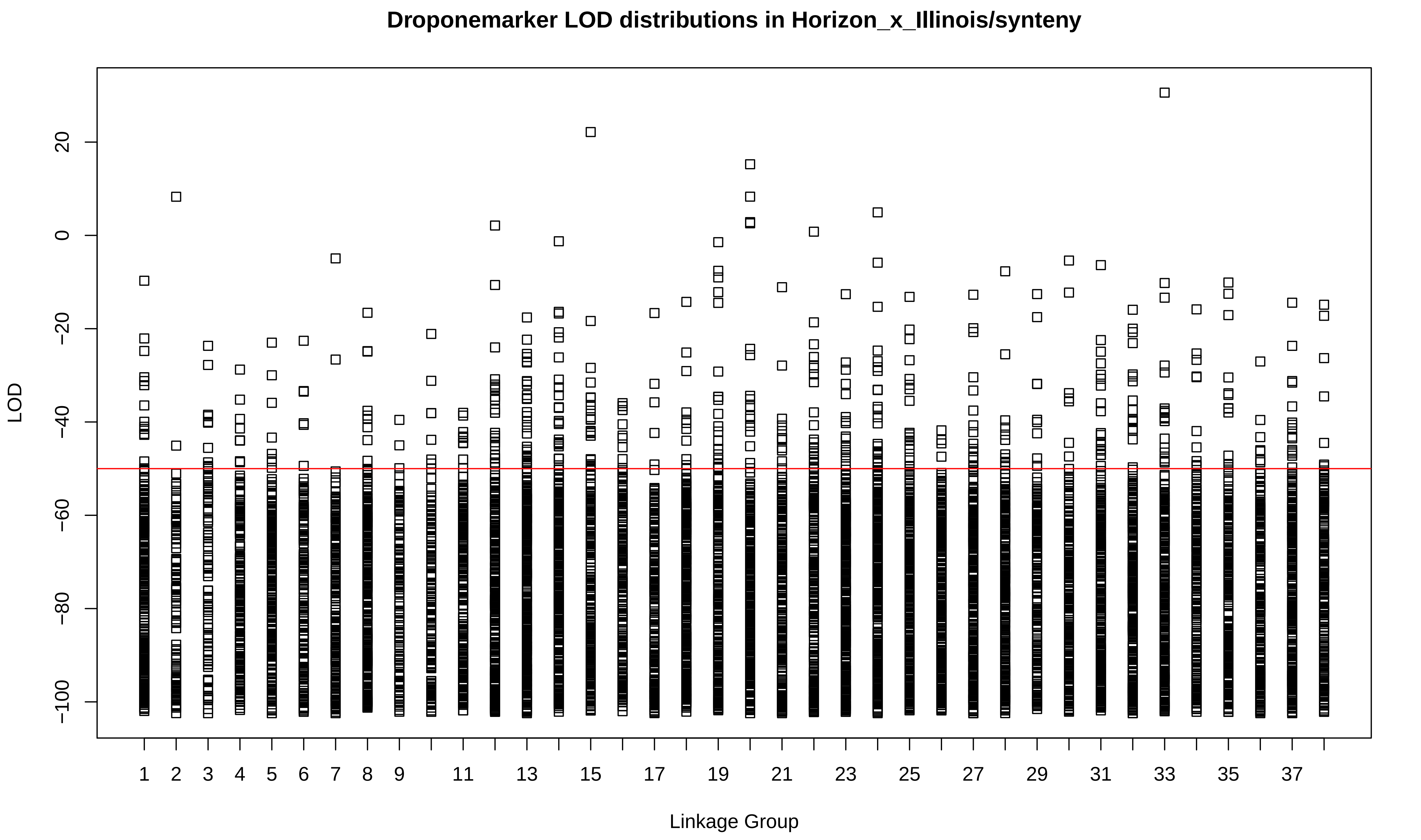

Droponemarker LOD distributions in Horizon_x_Illinois/synteny
LOD
20
0
−20
−40
−60
−80
−100
1
2
3
4
5
6
7
8
9
11
13
15
17
19
21
23
25
27
29
31
33
35
37
Linkage Group

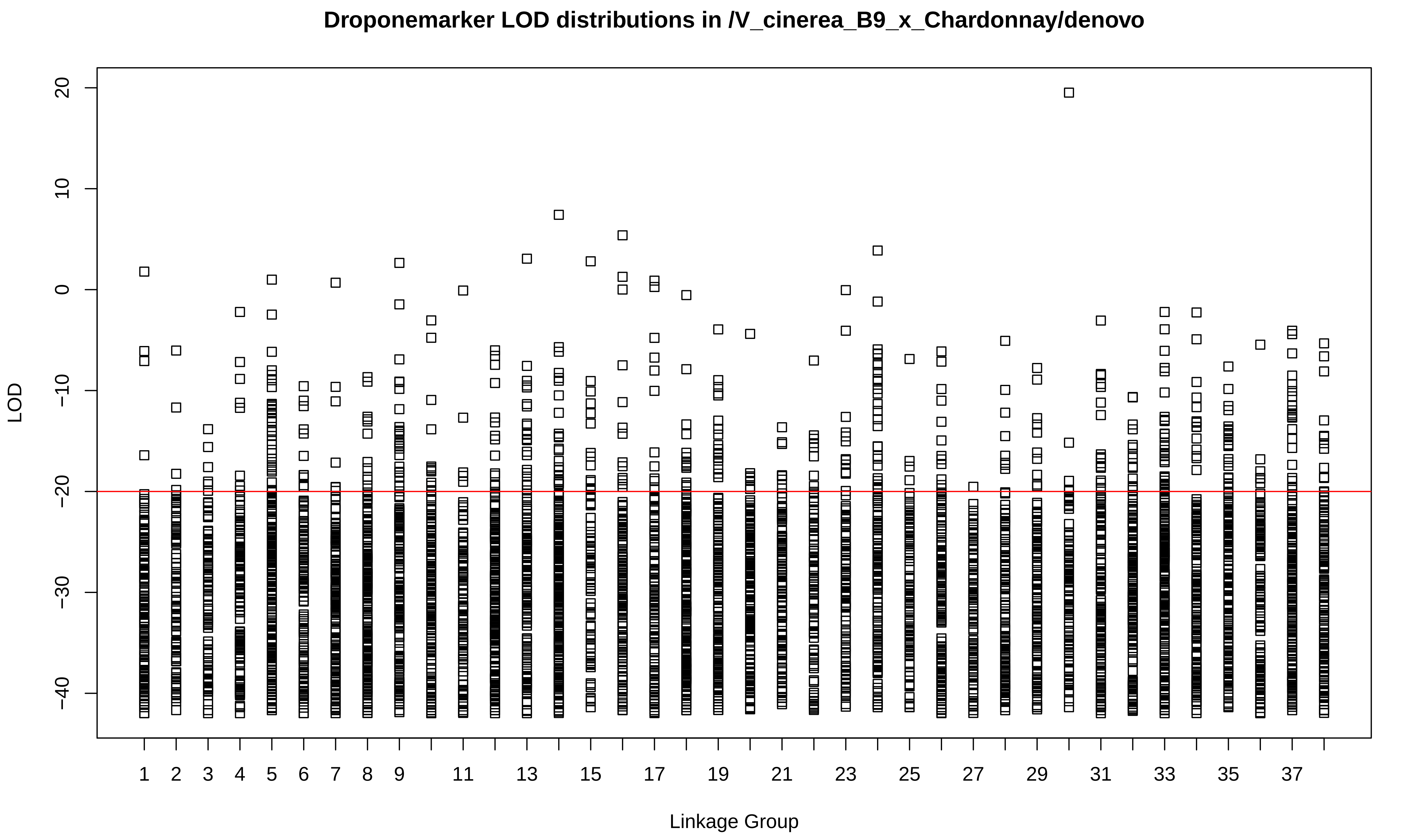
Droponemarker LOD distributions in /V_cinerea_B9_x_Chardonnay/denovo
LOD
20
10
0
−10
−20
−30
−40
Linkage Group
1
2
3
4
5
6
7
8
9
11
13
15
17
19
21
23
25
27
29
31
33
35
37

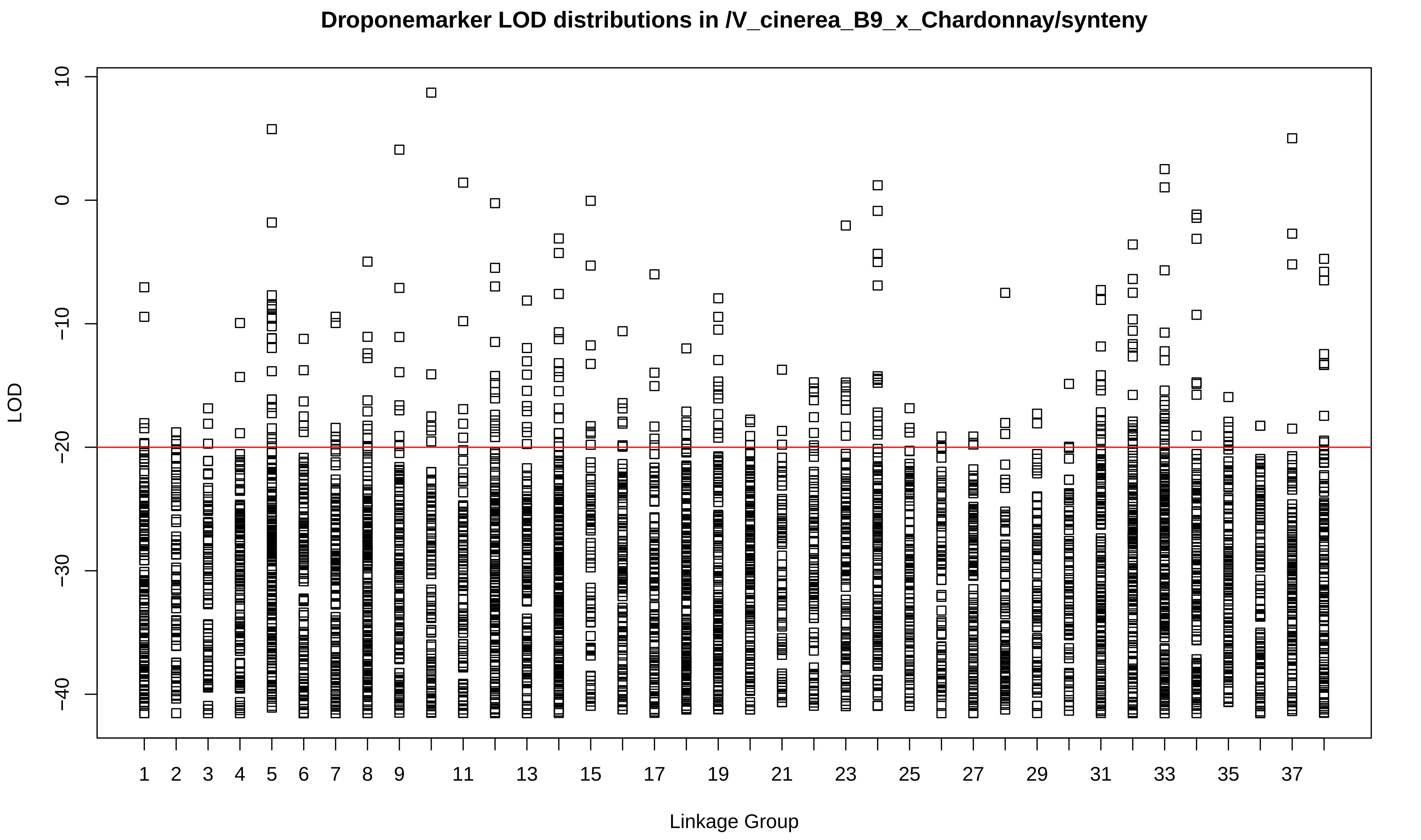
Droponemarker LOD distributions in /V_cinerea_B9_x_Chardonnay/synteny
LOD
10
0
−10
−20
−30
−40
1
2
3
4
5
6
7
8
9
11
13
15
17
19
21
23
25
27
29
31
33
35
37
Linkage Group

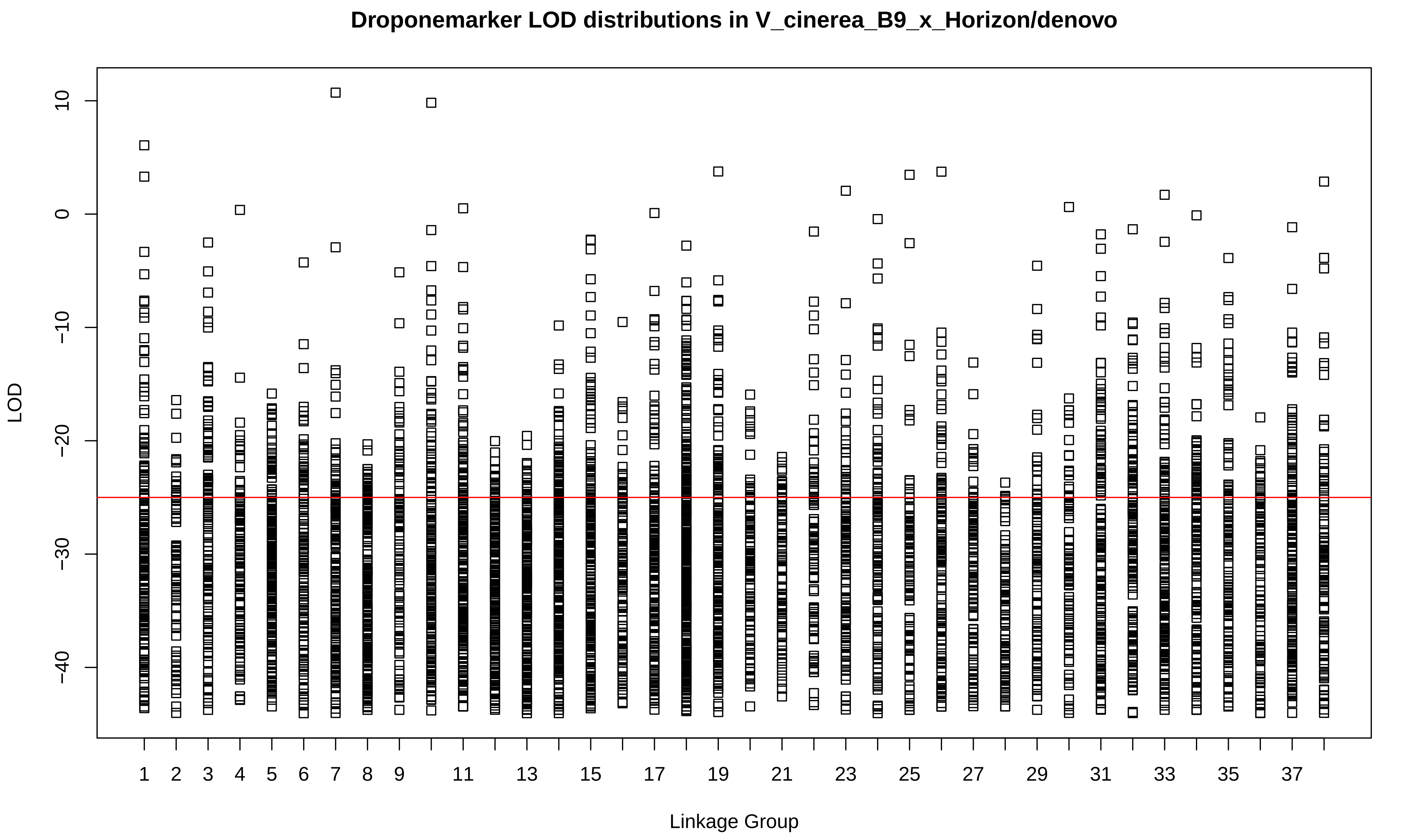

Droponemarker LOD distributions in V_cinerea_B9_x_Horizon/denovo
LOD
10
0
−10
−20
−30
−40
1
2
3
4
5
6
7
8
9
11
13
15
17
19
21
23
25
27
29
31
33
35
37
Linkage Group

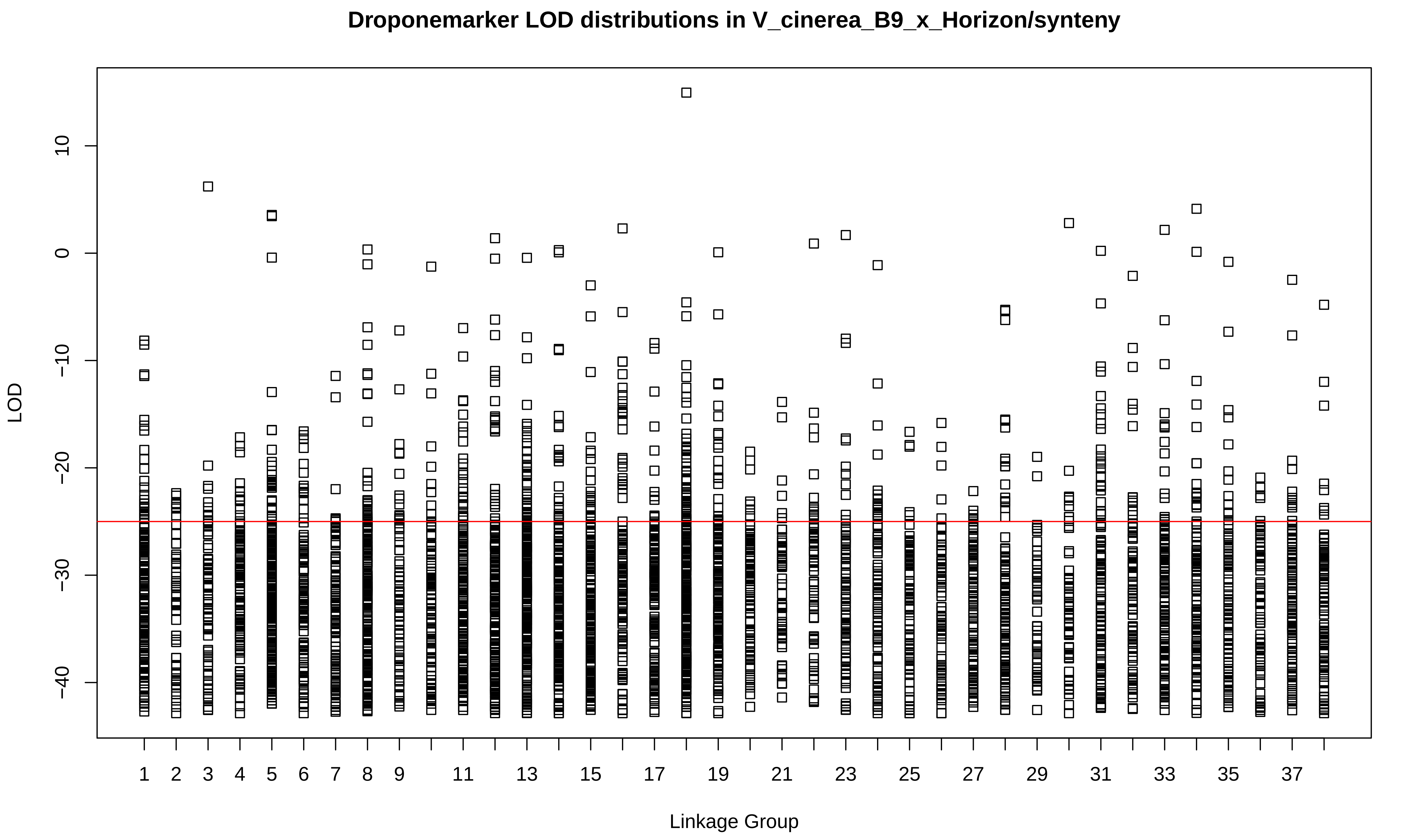
Droponemarker LOD distributions in V_cinerea_B9_x_Horizon/synteny
LOD
10
0
−10
−20
−30
−40
Linkage Group
1 2 3 4 5 6 7 8 9 11 13 15 17 19 21 23 25 27 29 31 33 35 37

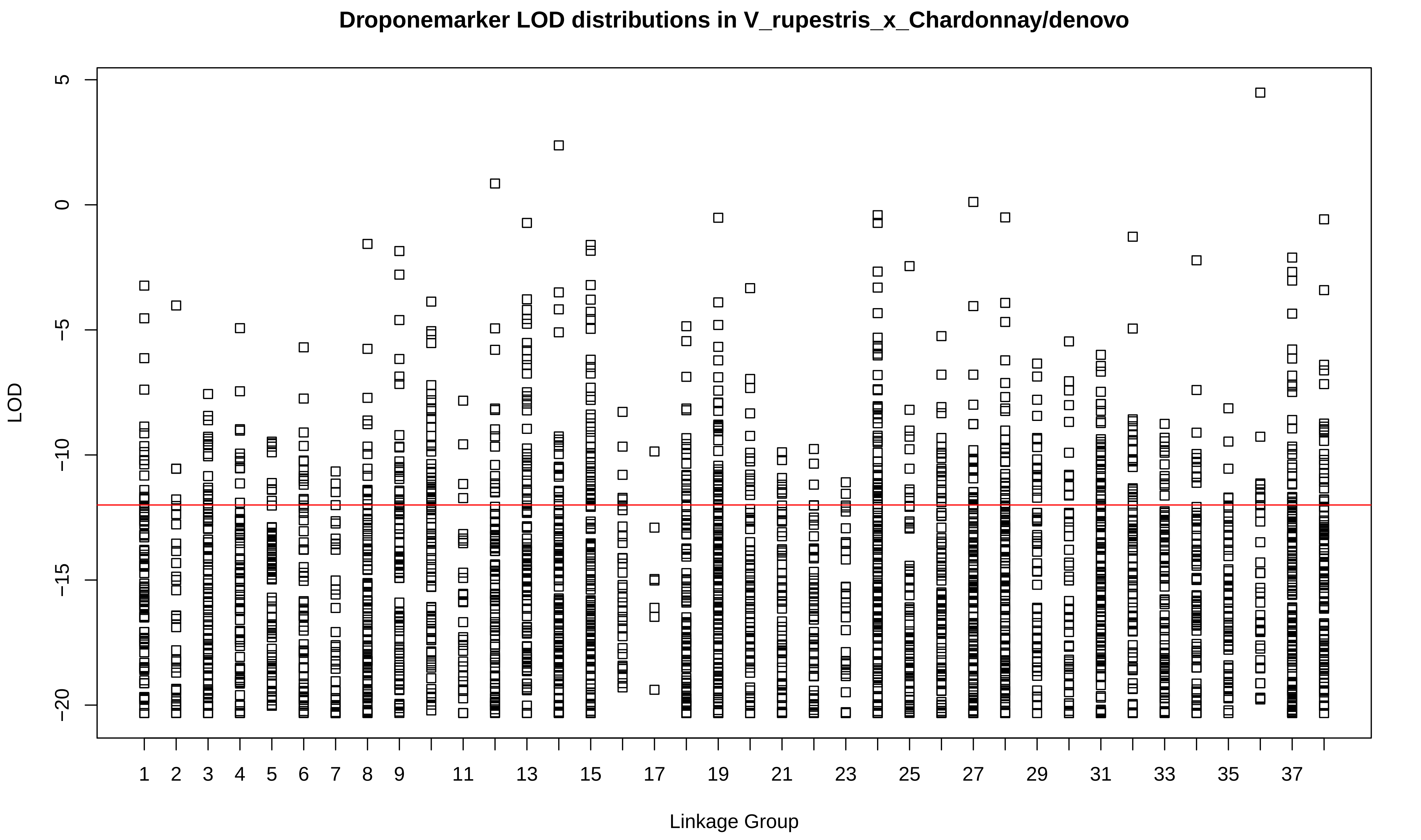
Droponemarker LOD distributions in V_rupestris_x_Chardonnay/denovo
LOD
5
0
−5
−10
−15
−20
1
2
3
4
5
6
7
8
9
11
13
15
17
19
21
23
25
27
29
31
33
35
37
Linkage Group

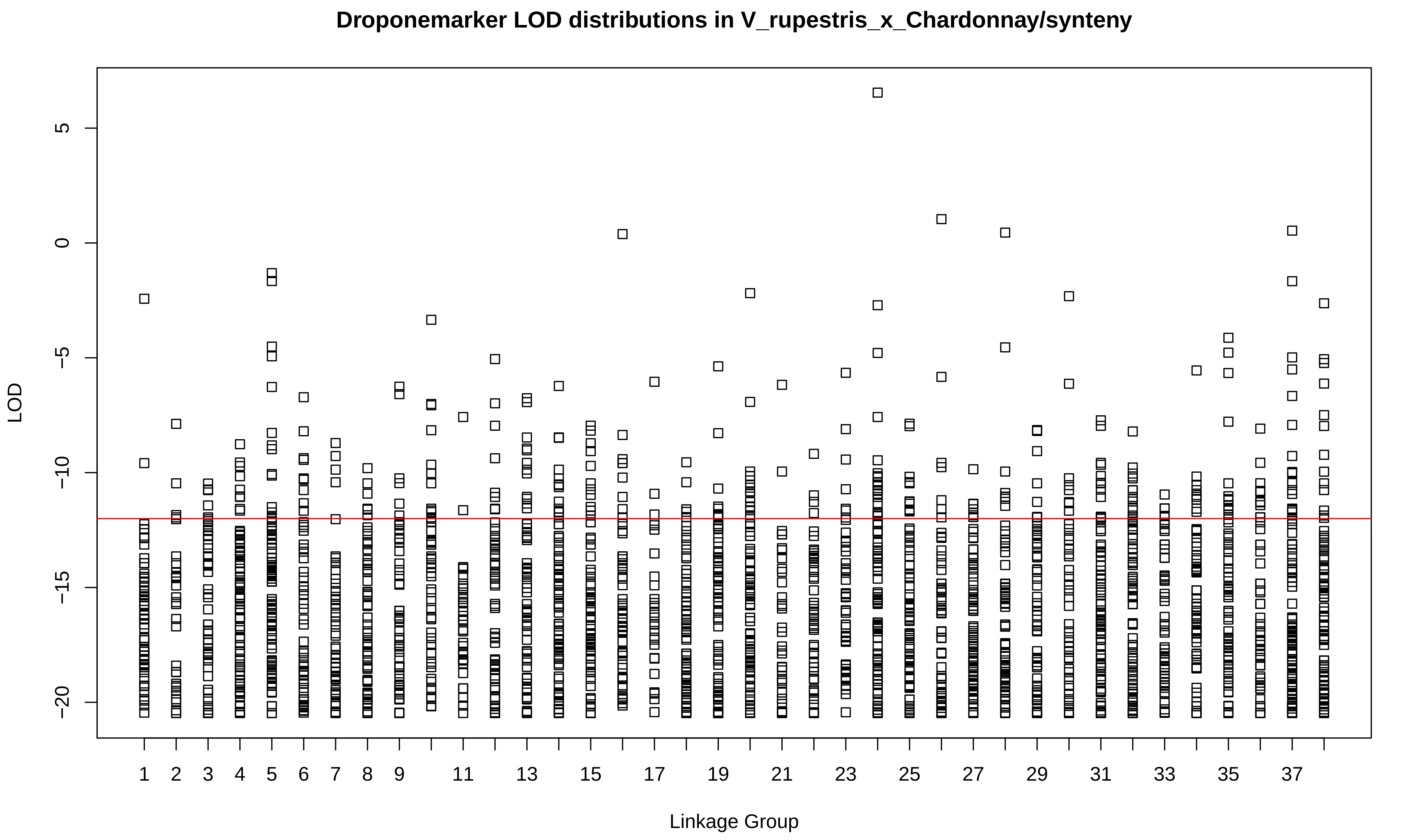

Droponemarker LOD distributions in V_rupestris_x_Chardonnay/synteny
LOD
5
0
−5
−10
−15
−20
1
2
3
4
5
6
7
8
9
11
13
15
17
19
21
23
25
27
29
31
33
35
37
Linkage Group
