# Supplementary material for: Heterozygous Mapping Strategy (HetMappS) for High Resolution Genotyping-By-Sequencing Markers: A Case Study in Grapevine
Source: PLoS One. 2015 Aug 5;10(8):e0134880. doi: 10.1371/journal.pone.0134880 (PMC4526651; doi:10.1371/journal.pone.0134880)
Supplement: S7 File — Genetic maps were independently generated using both synteny and de novo HetMappS pipelines. (PDF) [file pone.0134880.s015.pdf]

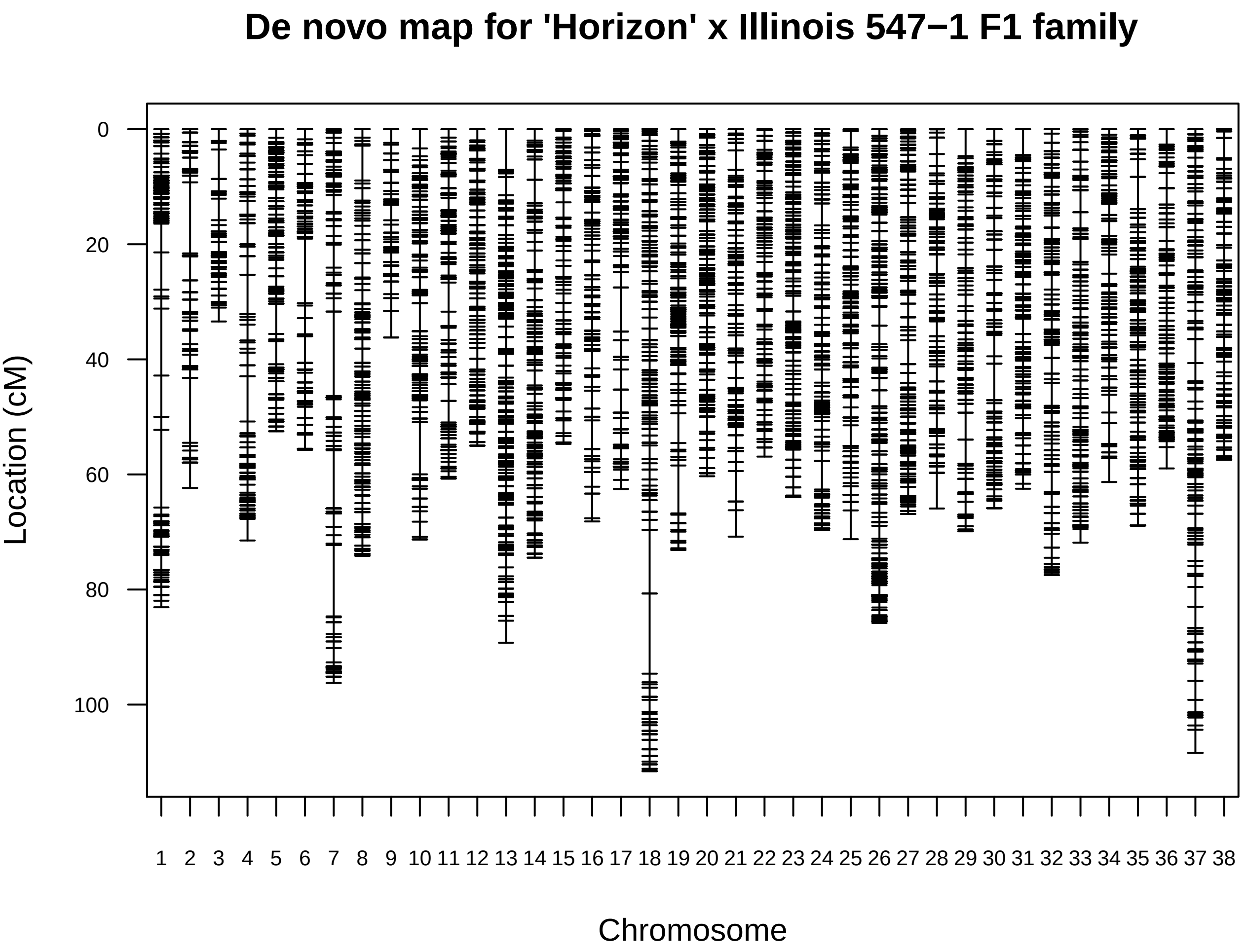
De novo map for 'Horizon' x Illinois 547−1 F1 family
Location (cM)
0
20
40
60
80
100
1 2 3 4 5 6 7 8 9 10 11 12 13 14 15 16 17 18 19 20 21 22 23 24 25 26 27 28 29 30 31 32 33 34 35 36 37 38
Chromosome

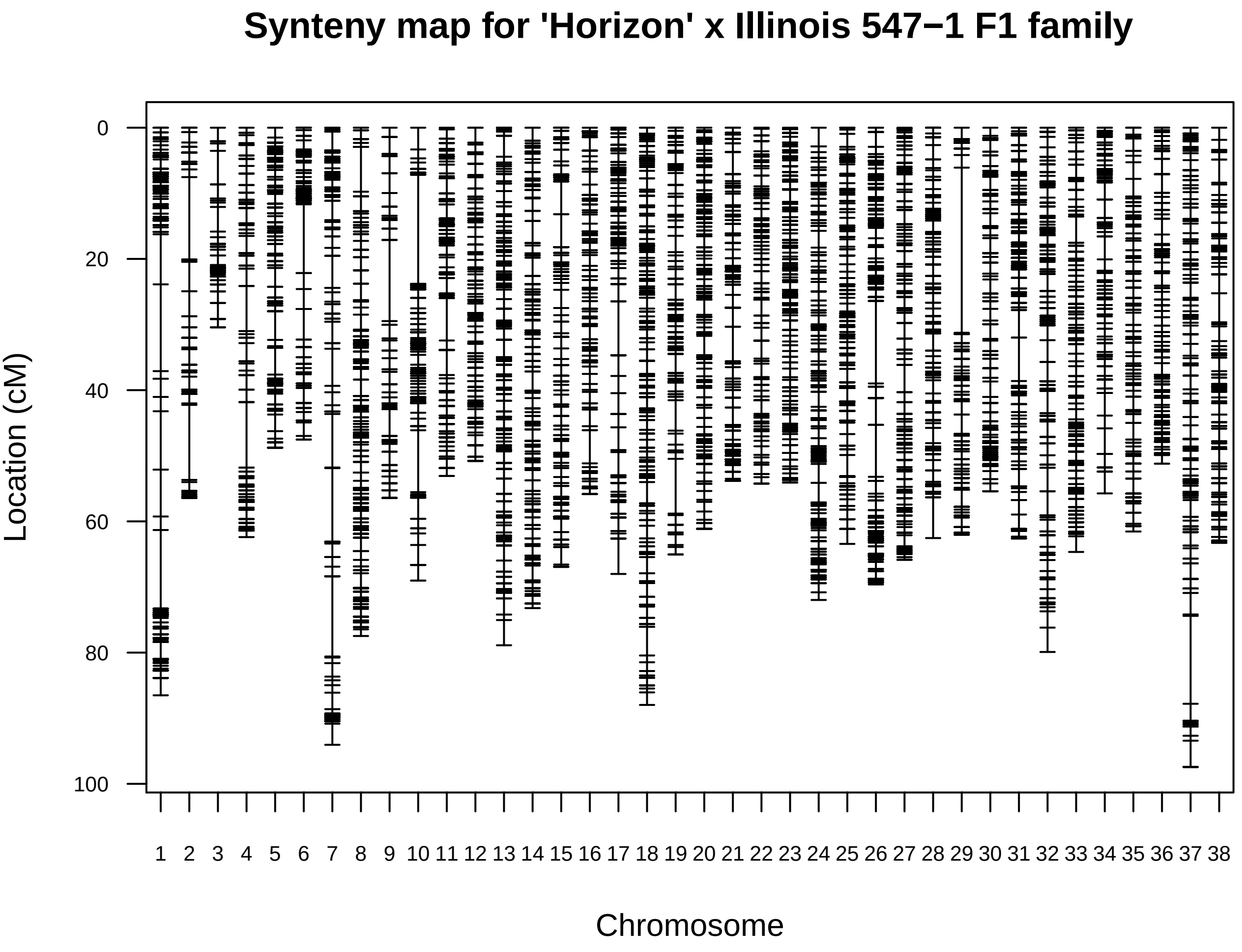
Synteny map for 'Horizon' x Illinois 547−1 F1 family
Location (cM)
0
20
40
60
80
100
1 2 3 4 5 6 7 8 9 10 11 12 13 14 15 16 17 18 19 20 21 22 23 24 25 26 27 28 29 30 31 32 33 34 35 36 37 38
Chromosome

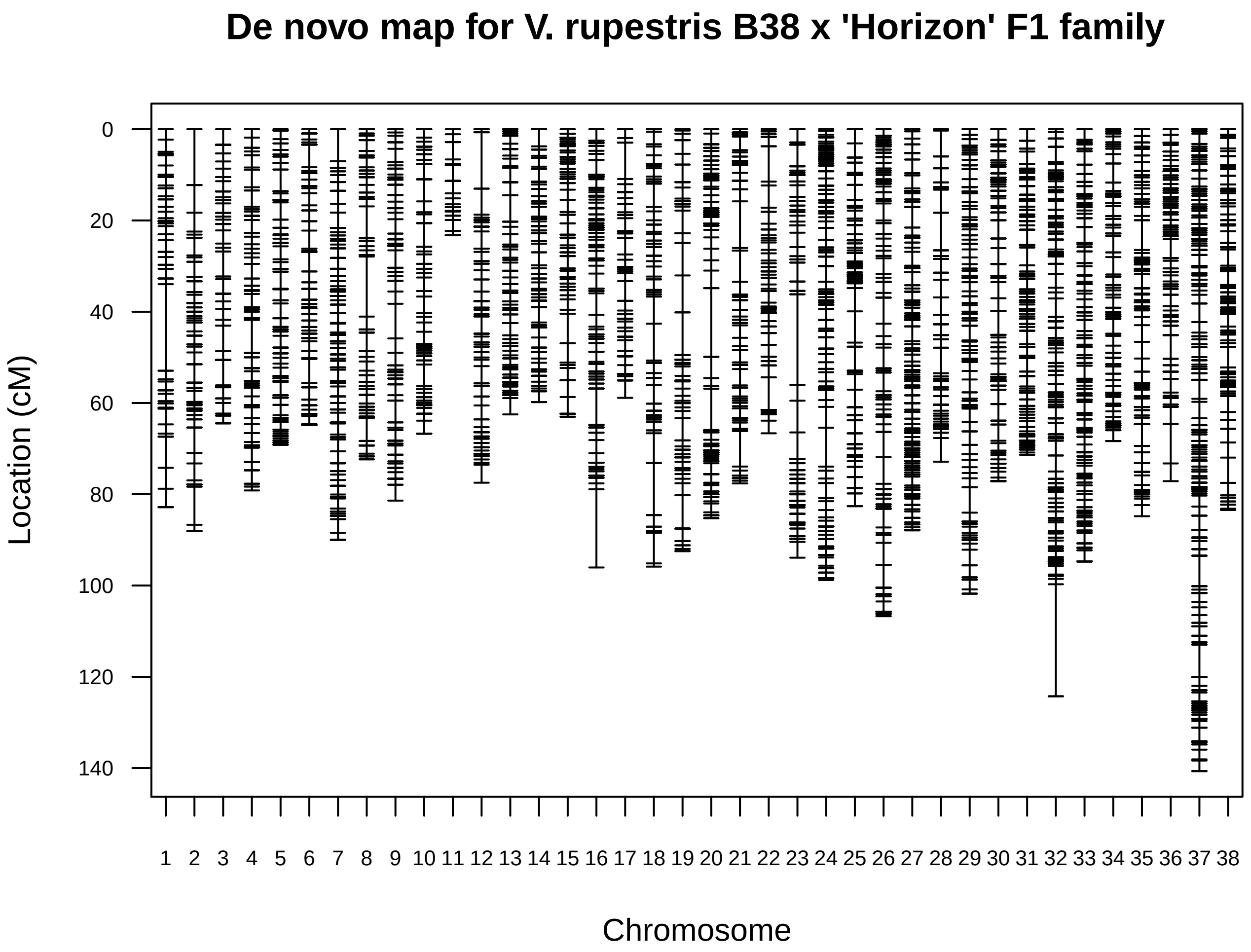
De novo map for V. rupestris B38 x 'Horizon' F1 family
Location (cM)
Chromosome

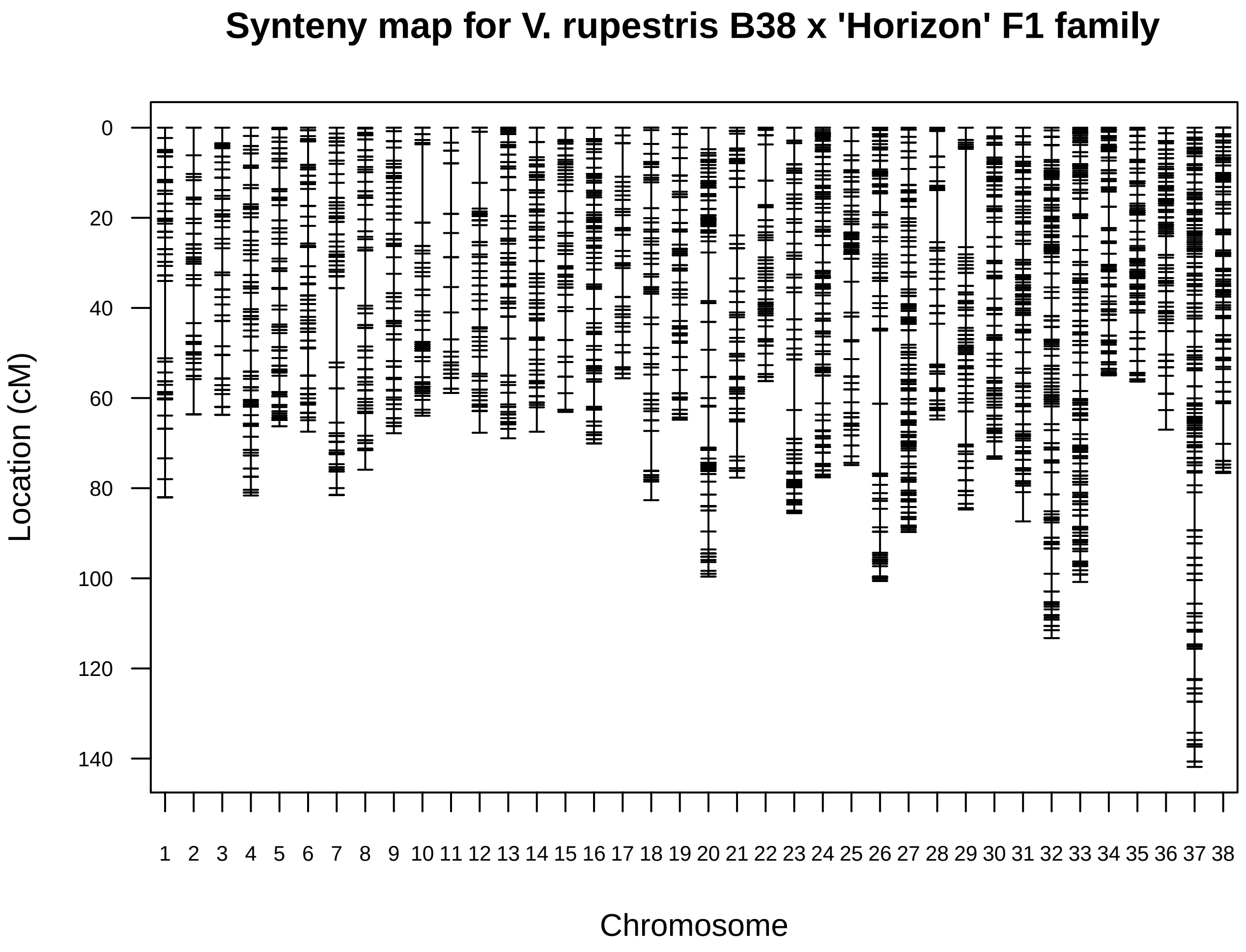
Synteny map for V. rupestris B38 x 'Horizon' F1 family
Location (cM)
Chromosome

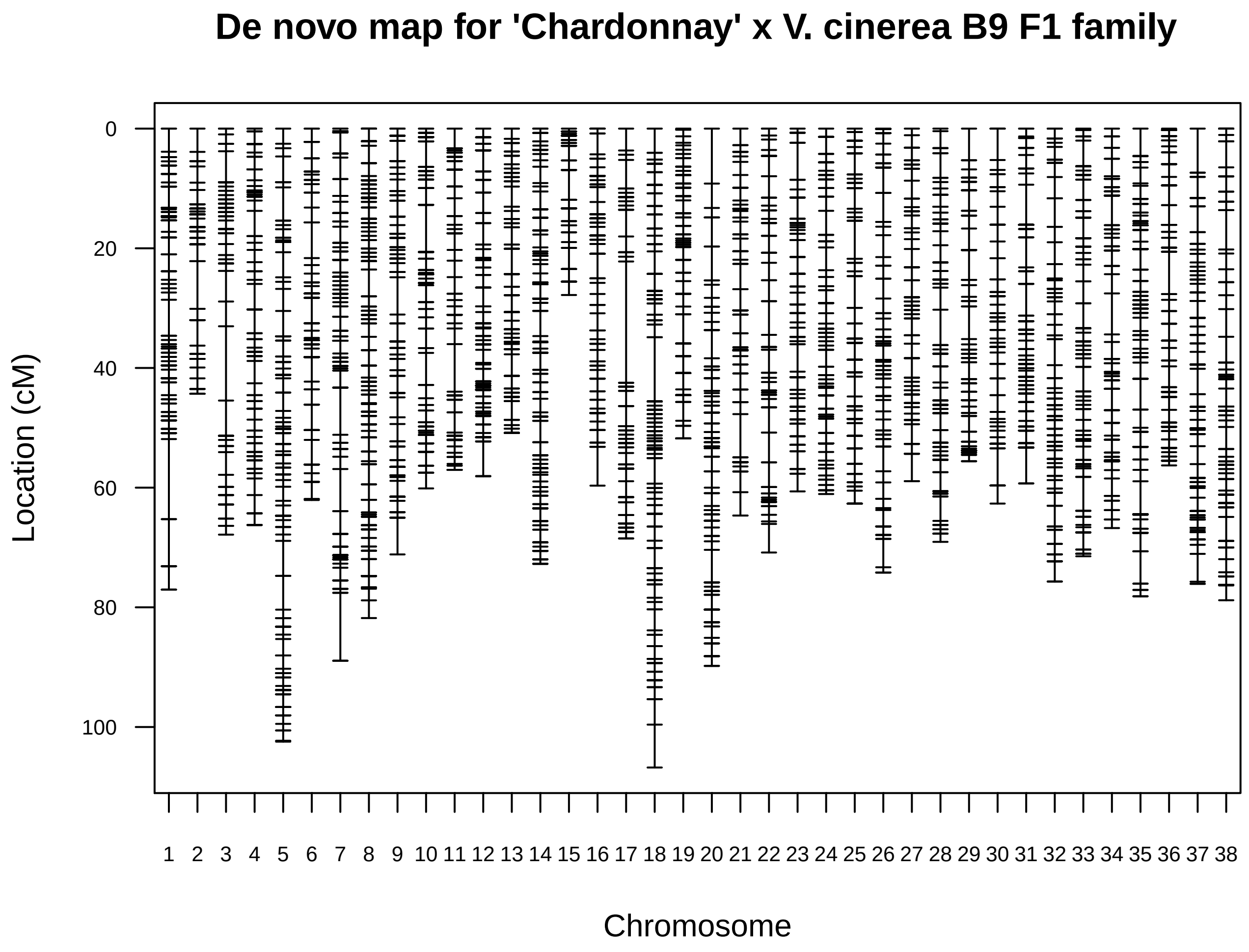
De novo map for 'Chardonnay' x V. cinerea B9 F1 family
Location (cM)
0
20
40
60
80
100
1 2 3 4 5 6 7 8 9 10 11 12 13 14 15 16 17 18 19 20 21 22 23 24 25 26 27 28 29 30 31 32 33 34 35 36 37 38
Chromosome

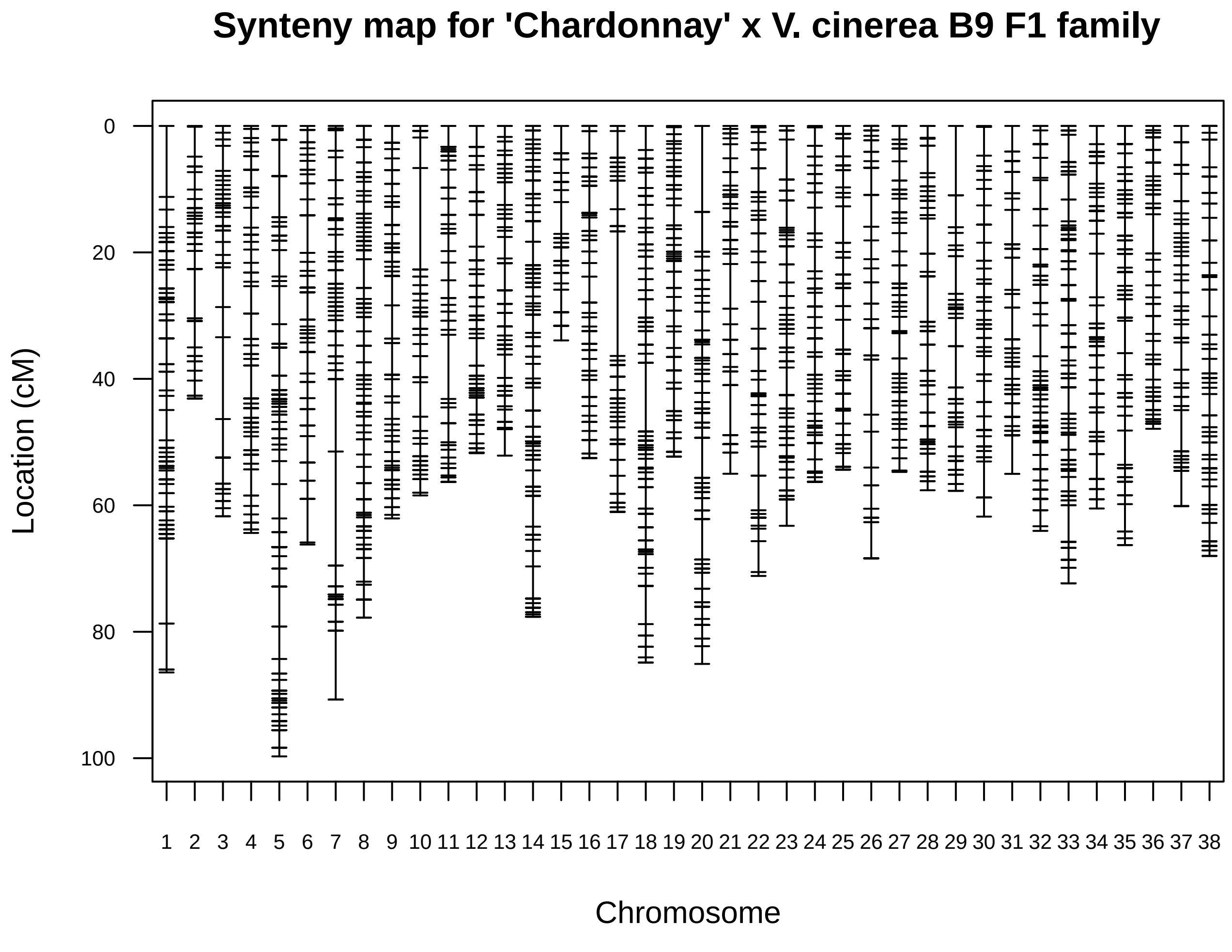
Synteny map for 'Chardonnay' x V. cinerea B9 F1 family
Location (cM)
0
20
40
60
80
100
1 2 3 4 5 6 7 8 9 10 11 12 13 14 15 16 17 18 19 20 21 22 23 24 25 26 27 28 29 30 31 32 33 34 35 36 37 38
Chromosome

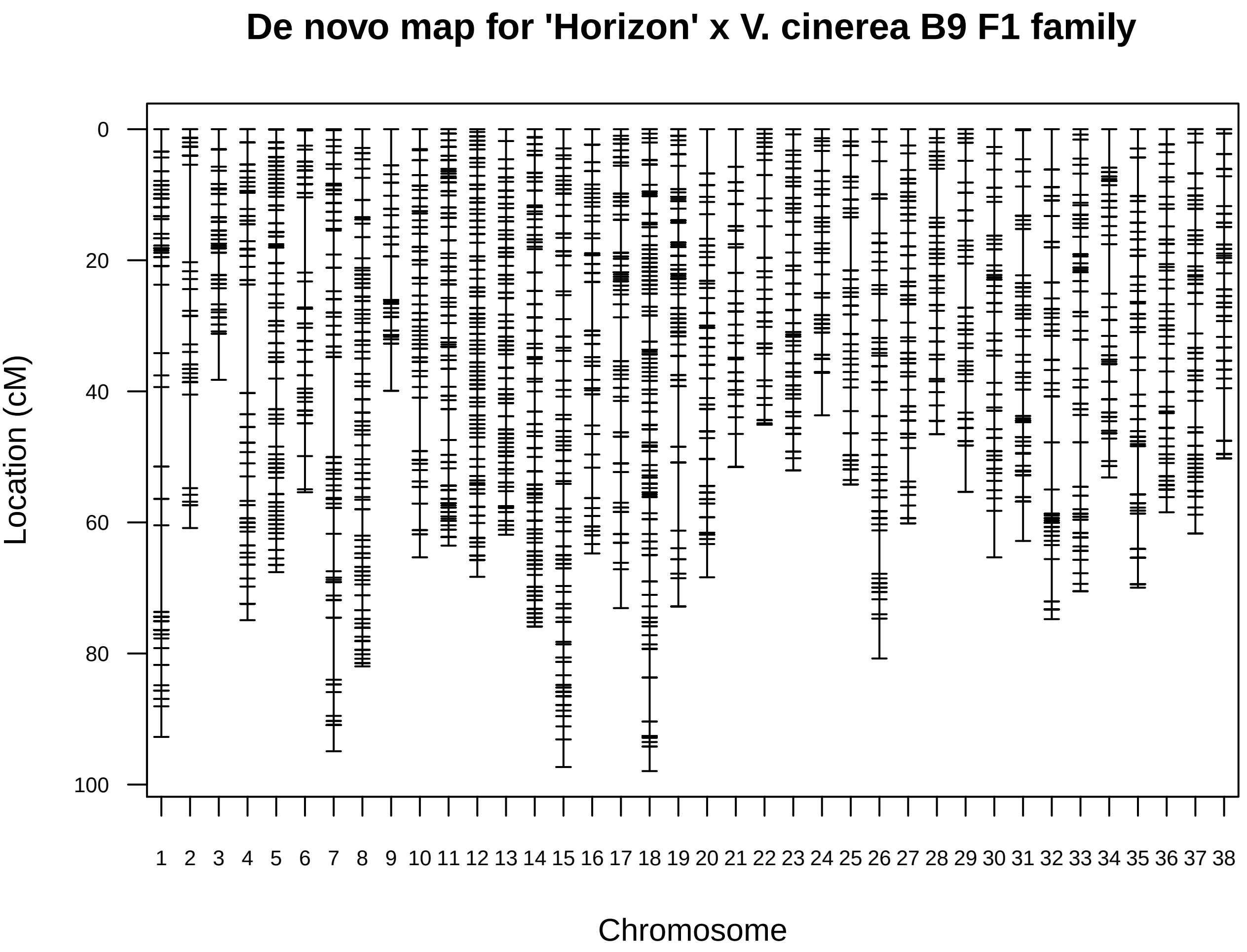
De novo map for 'Horizon' x V. cinerea B9 F1 family
Location (cM)
0
20
40
60
80
100
1 2 3 4 5 6 7 8 9 10 11 12 13 14 15 16 17 18 19 20 21 22 23 24 25 26 27 28 29 30 31 32 33 34 35 36 37 38
Chromosome

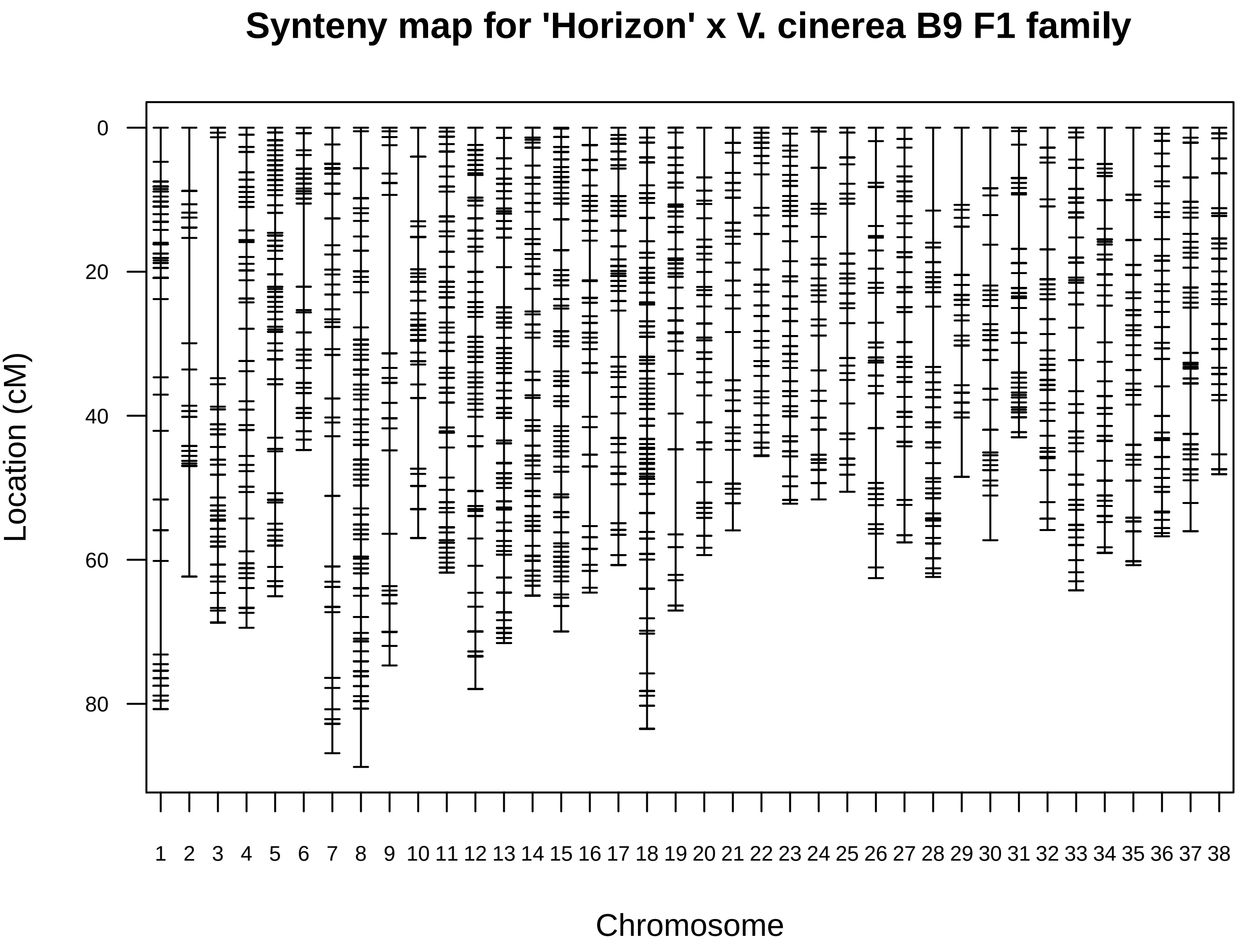
Synteny map for 'Horizon' x V. cinerea B9 F1 family
Location (cM)
0
20
40
60
80
1 2 3 4 5 6 7 8 9 10 11 12 13 14 15 16 17 18 19 20 21 22 23 24 25 26 27 28 29 30 31 32 33 34 35 36 37 38
Chromosome

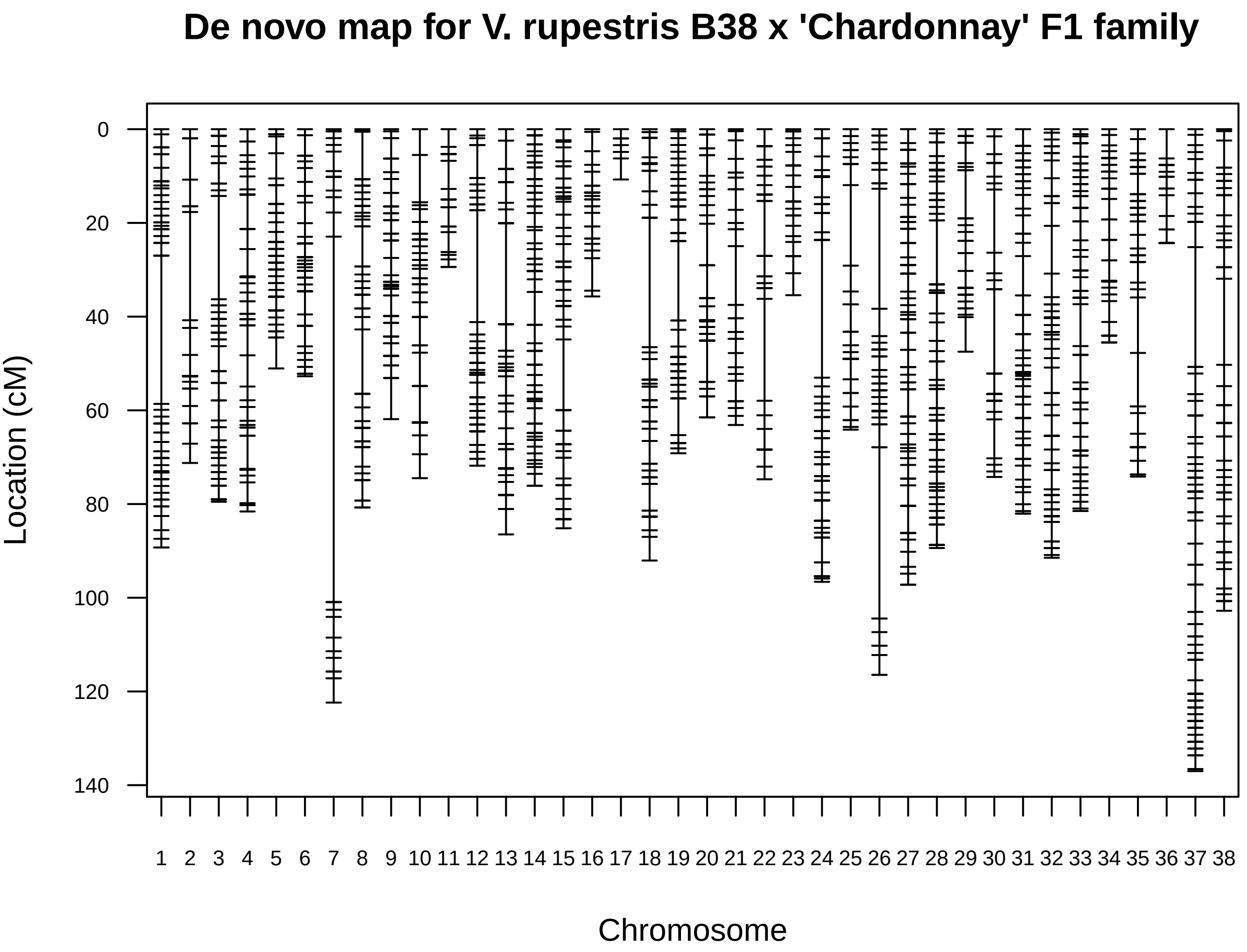
De novo map for V. rupestris B38 x 'Chardonnay' F1 family
Location (cM)
0
20
40
60
80
100
120
140
1 2 3 4 5 6 7 8 9 10 11 12 13 14 15 16 17 18 19 20 21 22 23 24 25 26 27 28 29 30 31 32 33 34 35 36 37 38
Chromosome

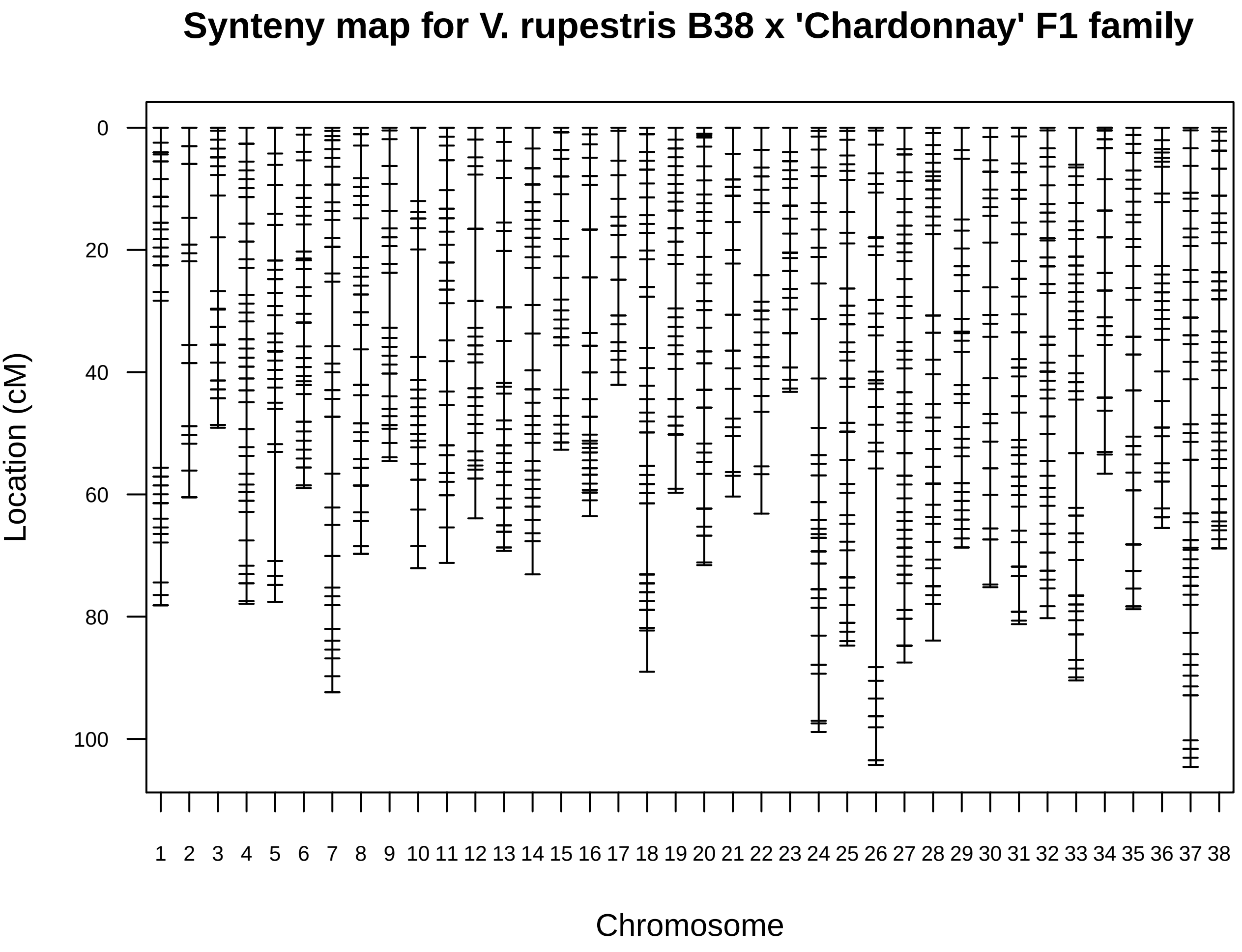
Synteny map for V. rupestris B38 x 'Chardonnay' F1 family
Location (cM)
0
20
40
60
80
100
1 2 3 4 5 6 7 8 9 10 11 12 13 14 15 16 17 18 19 20 21 22 23 24 25 26 27 28 29 30 31 32 33 34 35 36 37 38
Chromosome
