# Supplementary material for: Heterozygous Mapping Strategy (HetMappS) for High Resolution Genotyping-By-Sequencing Markers: A Case Study in Grapevine
Source: PLoS One. 2015 Aug 5;10(8):e0134880. doi: 10.1371/journal.pone.0134880 (PMC4526651; doi:10.1371/journal.pone.0134880)
Supplement: S8 File — Genetic map were independently generated using synteny and de novo pipelines over different sets of progeny. (PDF) [file pone.0134880.s016.pdf]

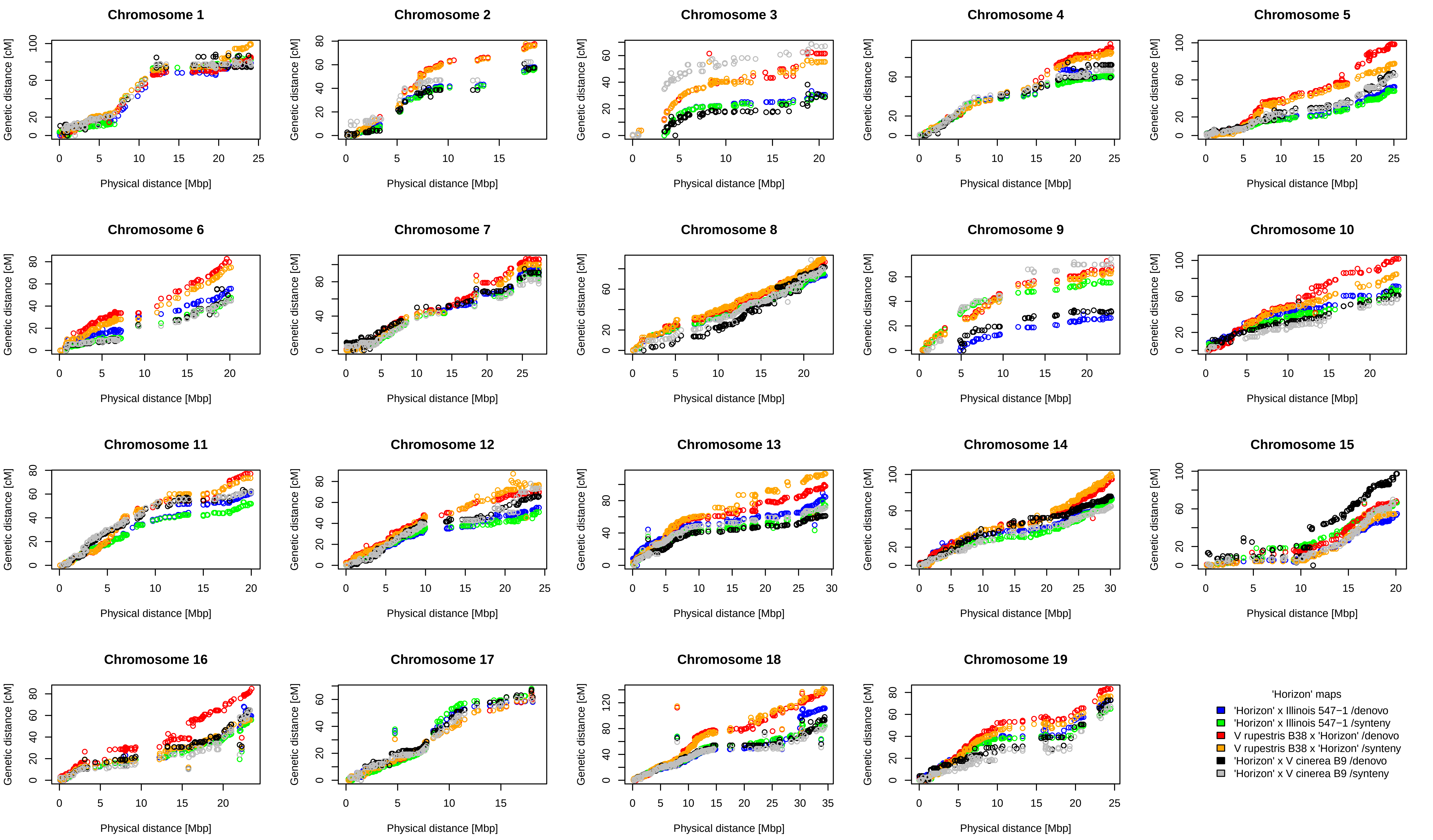
Chromosome 1
Chromosome 2
Chromosome 3
Chromosome 4
Chromosome 5
Chromosome 6
Chromosome 7
Chromosome 8
Chromosome 9
Chromosome 10
Chromosome 11
Chromosome 12
Chromosome 13
Chromosome 14
Chromosome 15
Chromosome 16
Chromosome 17
Chromosome 18
Chromosome 19
Genetic distance [cM]
Physical distance [Mbp]
'Horizon' maps
'Horizon' x Illinois 547−1 /denovo
'Horizon' x Illinois 547−1 /synteny
V rupestris B38 x 'Horizon' /denovo
V rupestris B38 x 'Horizon' /synteny
'Horizon' x V cinerea B9 /denovo
'Horizon' x V cinerea B9 /synteny

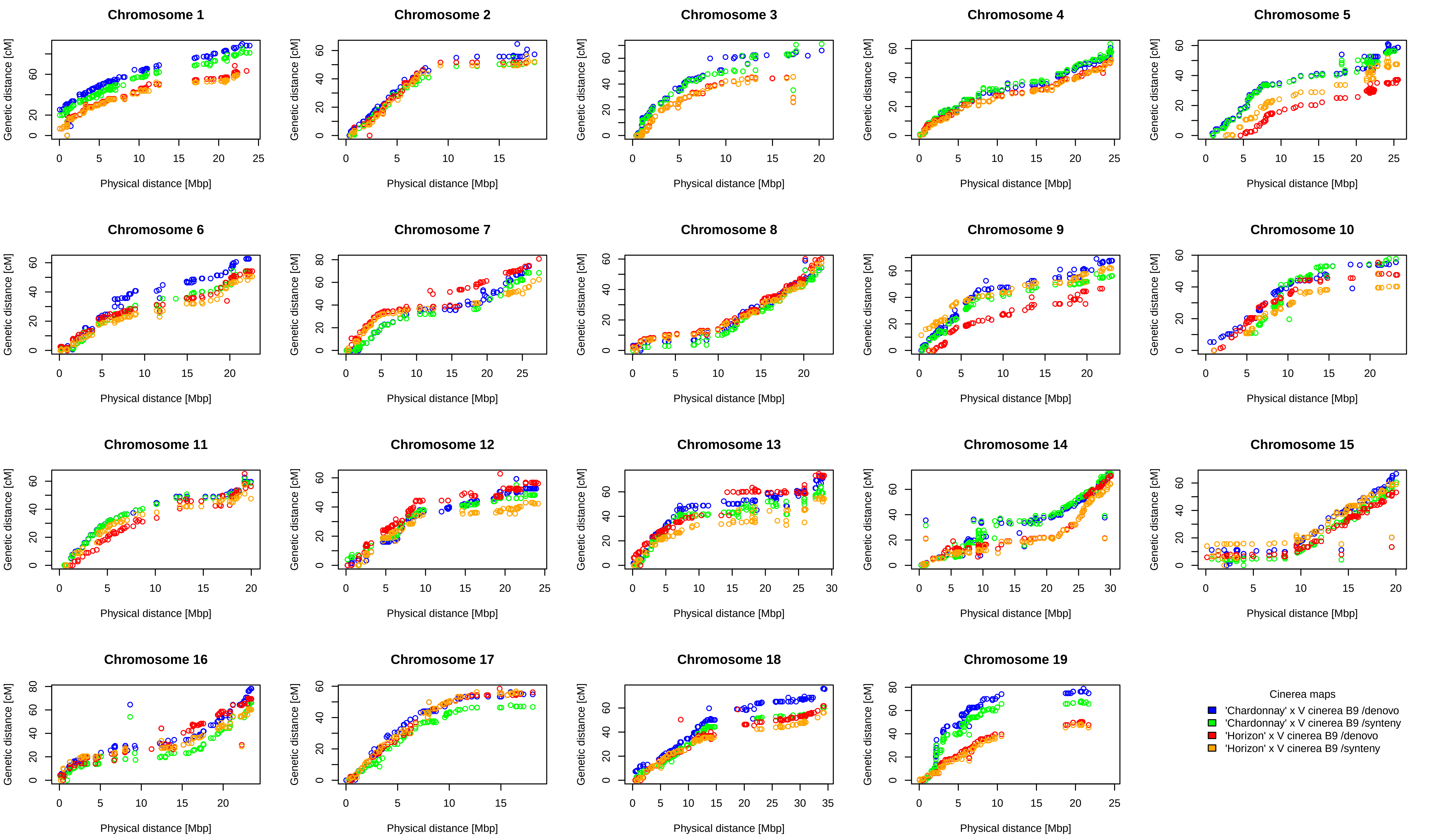
Chromosome 1
Chromosome 2
Chromosome 3
Chromosome 4
Chromosome 5
Chromosome 6
Chromosome 7
Chromosome 8
Chromosome 9
Chromosome 10
Chromosome 11
Chromosome 12
Chromosome 13
Chromosome 14
Chromosome 15
Chromosome 16
Chromosome 17
Chromosome 18
Chromosome 19
Genetic distance [cM]
Physical distance [Mbp]
Cinerea maps
'Chardonnay' x V cinerea B9 /denovo
'Chardonnay' x V cinerea B9 /synteny
'Horizon' x V cinerea B9 /denovo
'Horizon' x V cinerea B9 /synteny

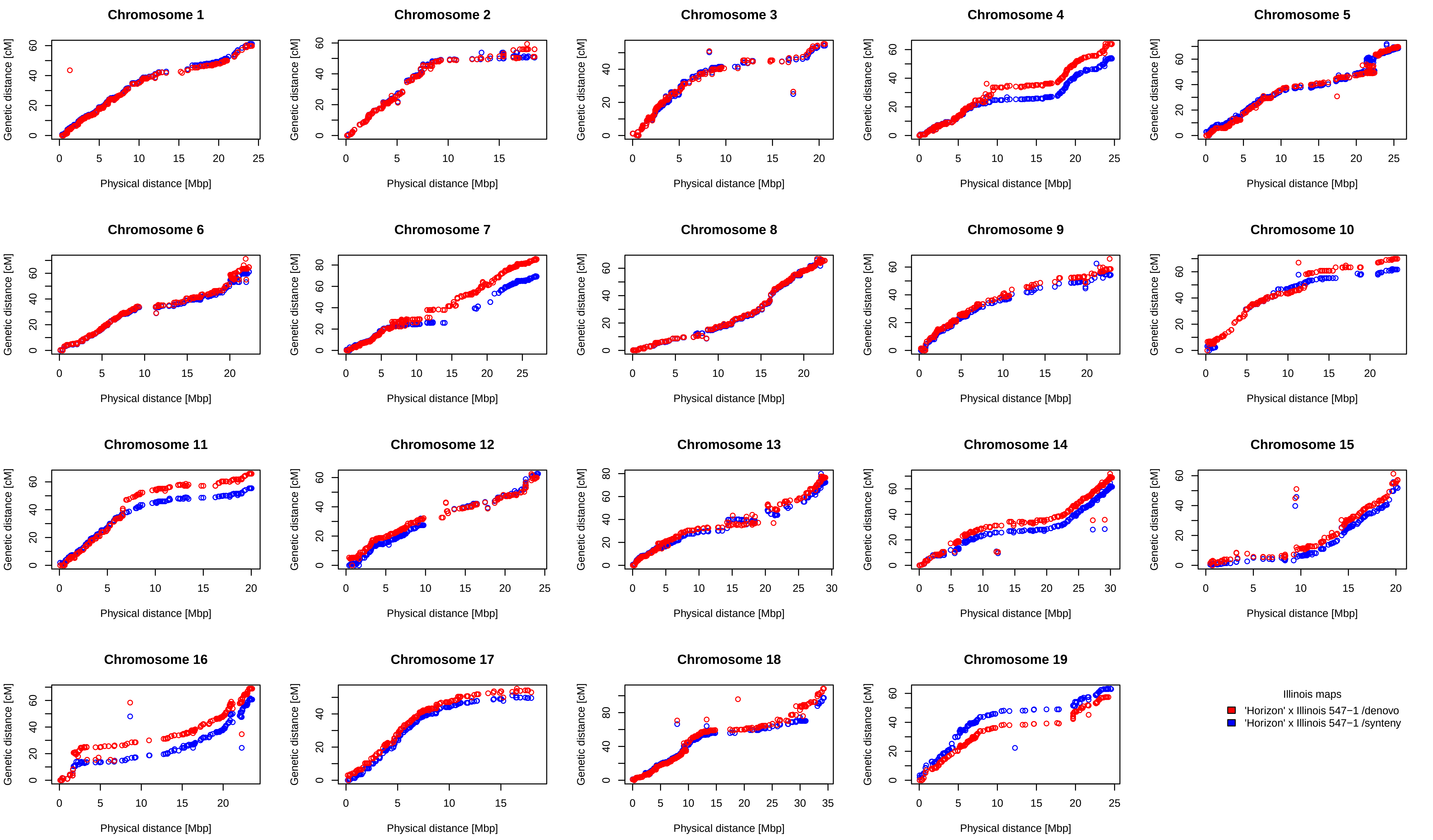
Chromosome 1
Chromosome 2
Chromosome 3
Chromosome 4
Chromosome 5
Chromosome 6
Chromosome 7
Chromosome 8
Chromosome 9
Chromosome 10
Chromosome 11
Chromosome 12
Chromosome 13
Chromosome 14
Chromosome 15
Chromosome 16
Chromosome 17
Chromosome 18
Chromosome 19
Genetic distance [cM]
Physical distance [Mbp]
Illinois maps
'Horizon' x Illinois 547−1 /denovo
'Horizon' x Illinois 547−1 /synteny

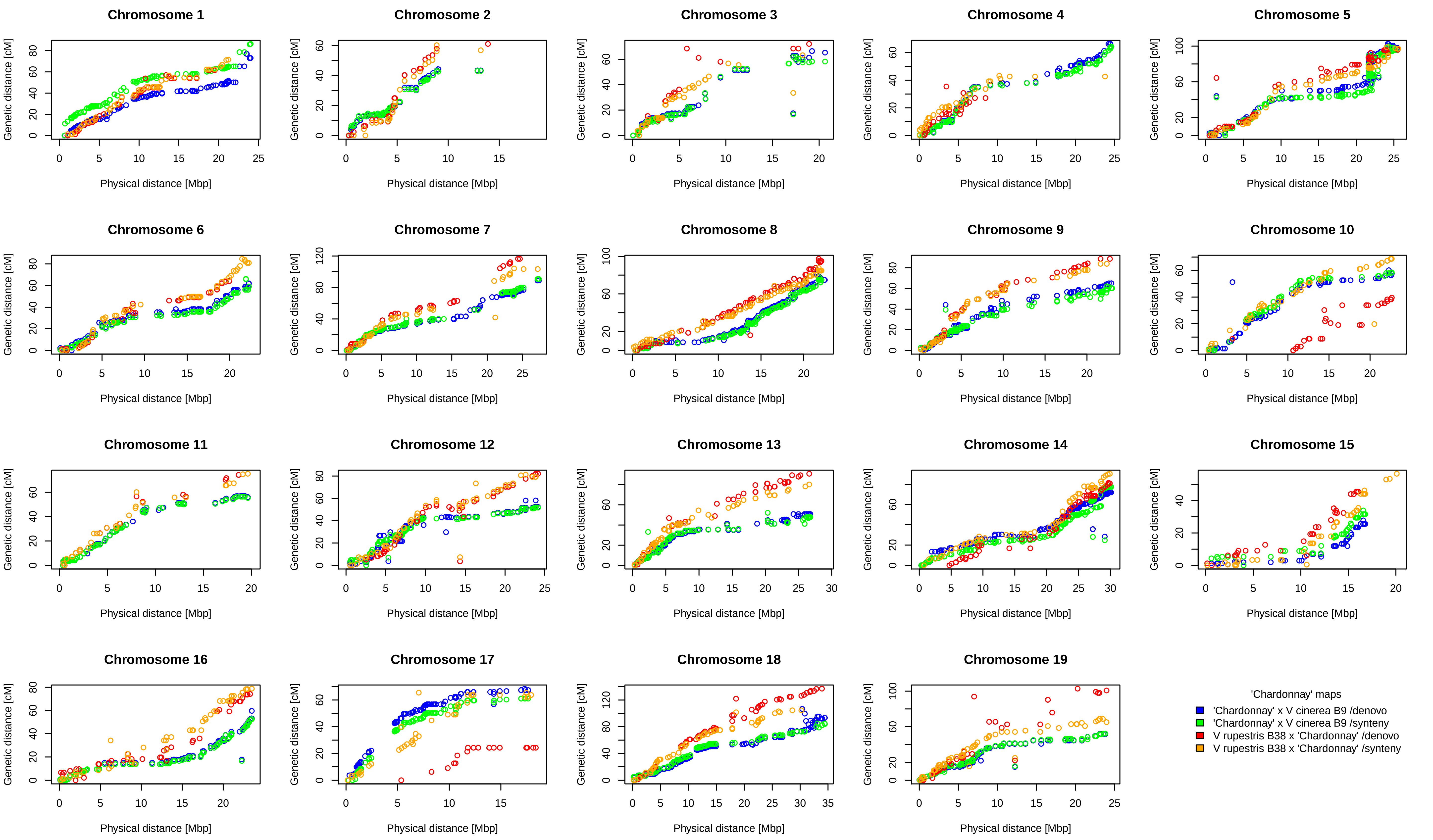
Chromosome 1
Chromosome 2
Chromosome 3
Chromosome 4
Chromosome 5
Chromosome 6
Chromosome 7
Chromosome 8
Chromosome 9
Chromosome 10
Chromosome 11
Chromosome 12
Chromosome 13
Chromosome 14
Chromosome 15
Chromosome 16
Chromosome 17
Chromosome 18
Chromosome 19
Genetic distance [cM]
Physical distance [Mbp]
'Chardonnay' maps
'Chardonnay' x V cinerea B9 /denovo
'Chardonnay' x V cinerea B9 /synteny
V rupestris B38 x 'Chardonnay' /denovo
V rupestris B38 x 'Chardonnay' /synteny

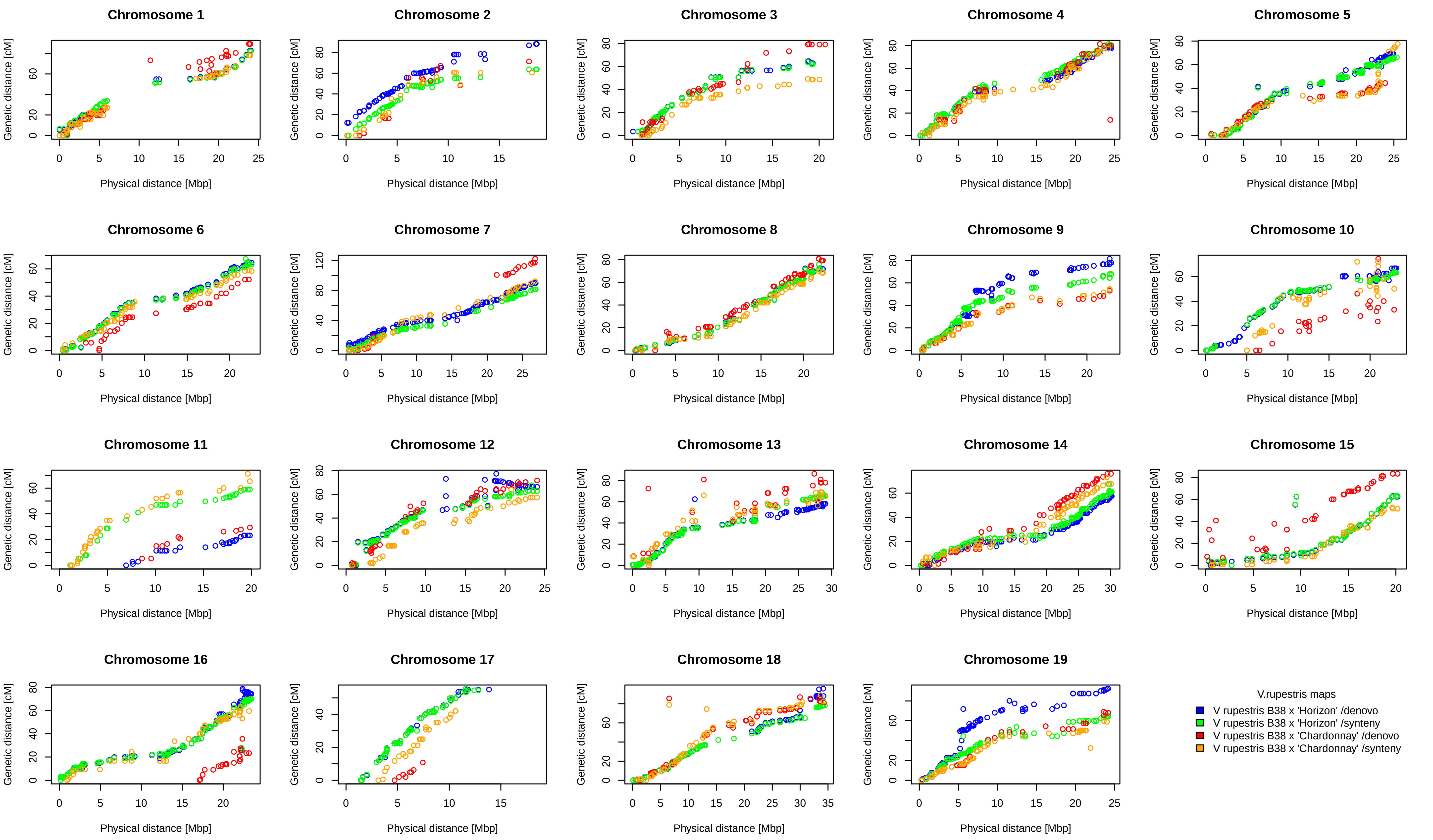
Chromosome 1
Chromosome 2
Chromosome 3
Chromosome 4
Chromosome 5
Chromosome 6
Chromosome 7
Chromosome 8
Chromosome 9
Chromosome 10
Chromosome 11
Chromosome 12
Chromosome 13
Chromosome 14
Chromosome 15
Chromosome 16
Chromosome 17
Chromosome 18
Chromosome 19
Genetic distance [cM]
Physical distance [Mbp]
V.rupestris maps
V rupestris B38 x 'Horizon' /denovo
V rupestris B38 x 'Horizon' /synteny
V rupestris B38 x 'Chardonnay' /denovo
V rupestris B38 x 'Chardonnay' /synteny
