# Supplementary material for: Heterozygous Mapping Strategy (HetMappS) for High Resolution Genotyping-By-Sequencing Markers: A Case Study in Grapevine
Source: PLoS One. 2015 Aug 5;10(8):e0134880. doi: 10.1371/journal.pone.0134880 (PMC4526651; doi:10.1371/journal.pone.0134880)
Supplement: S1 Table — (DOCX) [file pone.0134880.s019.docx]

**S1 Table:** Sequences and barcodes comprising four 96-plex adapter sets used to generate 384-plex GBS libraries

| Adapter Set | Barcode | Top Strand Sequence (5'-3') | Bottom Strand Sequence (5'-3') |
| --- | --- | --- | --- |
| ApeKI_ E | AGGC | ACACTCTTTCCCTACACGACGCTCTTCCGATCTAGGC | CWGGCCTAGATCGGAAGAGCGTCGTGTAGGGAAAGAGTGT |
| ApeKI_ E | GATT | ACACTCTTTCCCTACACGACGCTCTTCCGATCTGATT | CWGAATCAGATCGGAAGAGCGTCGTGTAGGGAAAGAGTGT |
| ApeKI_ E | ACCGT | ACACTCTTTCCCTACACGACGCTCTTCCGATCTACCGT | CWGACGGTAGATCGGAAGAGCGTCGTGTAGGGAAAGAGTGT |
| ApeKI_ E | CGTCA | ACACTCTTTCCCTACACGACGCTCTTCCGATCTCGTCA | CWGTGACGAGATCGGAAGAGCGTCGTGTAGGGAAAGAGTGT |
| ApeKI_ E | TCGCA | ACACTCTTTCCCTACACGACGCTCTTCCGATCTTCGCA | CWGTGCGAAGATCGGAAGAGCGTCGTGTAGGGAAAGAGTGT |
| ApeKI_ E | CGCAT | ACACTCTTTCCCTACACGACGCTCTTCCGATCTCGCAT | CWGATGCGAGATCGGAAGAGCGTCGTGTAGGGAAAGAGTGT |
| ApeKI_ E | TCATAGT | ACACTCTTTCCCTACACGACGCTCTTCCGATCTTCATAGT | CWGACTATGAAGATCGGAAGAGCGTCGTGTAGGGAAAGAGTGT |
| ApeKI_ E | TTACGAT | ACACTCTTTCCCTACACGACGCTCTTCCGATCTTTACGAT | CWGATCGTAAAGATCGGAAGAGCGTCGTGTAGGGAAAGAGTGT |
| ApeKI_ E | GGCTAGA | ACACTCTTTCCCTACACGACGCTCTTCCGATCTGGCTAGA | CWGTCTAGCCAGATCGGAAGAGCGTCGTGTAGGGAAAGAGTGT |
| ApeKI_ E | ACAATGGA | ACACTCTTTCCCTACACGACGCTCTTCCGATCTACAATGGA | CWGTCCATTGTAGATCGGAAGAGCGTCGTGTAGGGAAAGAGTGT |
| ApeKI_ E | ACAAGAGT | ACACTCTTTCCCTACACGACGCTCTTCCGATCTACAAGAGT | CWGACTCTTGTAGATCGGAAGAGCGTCGTGTAGGGAAAGAGTGT |
| ApeKI_ E | GAACATGA | ACACTCTTTCCCTACACGACGCTCTTCCGATCTGAACATGA | CWGTCATGTTCAGATCGGAAGAGCGTCGTGTAGGGAAAGAGTGT |
| ApeKI_ E | AGCATT | ACACTCTTTCCCTACACGACGCTCTTCCGATCTAGCATT | CWGAATGCTAGATCGGAAGAGCGTCGTGTAGGGAAAGAGTGT |
| ApeKI_ E | CTCCGA | ACACTCTTTCCCTACACGACGCTCTTCCGATCTCTCCGA | CWGTCGGAGAGATCGGAAGAGCGTCGTGTAGGGAAAGAGTGT |
| ApeKI_ E | TTGGCA | ACACTCTTTCCCTACACGACGCTCTTCCGATCTTTGGCA | CWGTGCCAAAGATCGGAAGAGCGTCGTGTAGGGAAAGAGTGT |
| ApeKI_ E | CCACGT | ACACTCTTTCCCTACACGACGCTCTTCCGATCTCCACGT | CWGACGTGGAGATCGGAAGAGCGTCGTGTAGGGAAAGAGTGT |
| ApeKI_ E | GATGTC | ACACTCTTTCCCTACACGACGCTCTTCCGATCTGATGTC | CWGGACATCAGATCGGAAGAGCGTCGTGTAGGGAAAGAGTGT |
| ApeKI_ E | TGTTAC | ACACTCTTTCCCTACACGACGCTCTTCCGATCTTGTTAC | CWGGTAACAAGATCGGAAGAGCGTCGTGTAGGGAAAGAGTGT |
| ApeKI_ E | CAGTTA | ACACTCTTTCCCTACACGACGCTCTTCCGATCTCAGTTA | CWGTAACTGAGATCGGAAGAGCGTCGTGTAGGGAAAGAGTGT |
| ApeKI_ E | GCCTAT | ACACTCTTTCCCTACACGACGCTCTTCCGATCTGCCTAT | CWGATAGGCAGATCGGAAGAGCGTCGTGTAGGGAAAGAGTGT |
| ApeKI_ E | AGTGGC | ACACTCTTTCCCTACACGACGCTCTTCCGATCTAGTGGC | CWGGCCACTAGATCGGAAGAGCGTCGTGTAGGGAAAGAGTGT |
| ApeKI_ E | TGACCT | ACACTCTTTCCCTACACGACGCTCTTCCGATCTTGACCT | CWGAGGTCAAGATCGGAAGAGCGTCGTGTAGGGAAAGAGTGT |
| ApeKI_ E | TTGCAC | ACACTCTTTCCCTACACGACGCTCTTCCGATCTTTGCAC | CWGGTGCAAAGATCGGAAGAGCGTCGTGTAGGGAAAGAGTGT |
| ApeKI_ E | CTAGCT | ACACTCTTTCCCTACACGACGCTCTTCCGATCTCTAGCT | CWGAGCTAGAGATCGGAAGAGCGTCGTGTAGGGAAAGAGTGT |
| ApeKI_ E | AATCGTT | ACACTCTTTCCCTACACGACGCTCTTCCGATCTAATCGTT | CWGAACGATTAGATCGGAAGAGCGTCGTGTAGGGAAAGAGTGT |
| ApeKI_ E | CTATGGA | ACACTCTTTCCCTACACGACGCTCTTCCGATCTCTATGGA | CWGTCCATAGAGATCGGAAGAGCGTCGTGTAGGGAAAGAGTGT |
| ApeKI_ E | TACGGTA | ACACTCTTTCCCTACACGACGCTCTTCCGATCTTACGGTA | CWGTACCGTAAGATCGGAAGAGCGTCGTGTAGGGAAAGAGTGT |
| ApeKI_ E | ACTATGT | ACACTCTTTCCCTACACGACGCTCTTCCGATCTACTATGT | CWGACATAGTAGATCGGAAGAGCGTCGTGTAGGGAAAGAGTGT |
| ApeKI_ E | CGTGAAT | ACACTCTTTCCCTACACGACGCTCTTCCGATCTCGTGAAT | CWGATTCACGAGATCGGAAGAGCGTCGTGTAGGGAAAGAGTGT |
| ApeKI_ E | TTGCAGA | ACACTCTTTCCCTACACGACGCTCTTCCGATCTTTGCAGA | CWGTCTGCAAAGATCGGAAGAGCGTCGTGTAGGGAAAGAGTGT |
| ApeKI_ E | AACTTGT | ACACTCTTTCCCTACACGACGCTCTTCCGATCTAACTTGT | CWGACAAGTTAGATCGGAAGAGCGTCGTGTAGGGAAAGAGTGT |
| ApeKI_ E | TGACGTA | ACACTCTTTCCCTACACGACGCTCTTCCGATCTTGACGTA | CWGTACGTCAAGATCGGAAGAGCGTCGTGTAGGGAAAGAGTGT |
| ApeKI_ E | GCTATAA | ACACTCTTTCCCTACACGACGCTCTTCCGATCTGCTATAA | CWGTTATAGCAGATCGGAAGAGCGTCGTGTAGGGAAAGAGTGT |
| ApeKI_ E | ATCGTAT | ACACTCTTTCCCTACACGACGCTCTTCCGATCTATCGTAT | CWGATACGATAGATCGGAAGAGCGTCGTGTAGGGAAAGAGTGT |
| ApeKI_ E | TACTGAT | ACACTCTTTCCCTACACGACGCTCTTCCGATCTTACTGAT | CWGATCAGTAAGATCGGAAGAGCGTCGTGTAGGGAAAGAGTGT |
| ApeKI_ E | CTTGAGA | ACACTCTTTCCCTACACGACGCTCTTCCGATCTCTTGAGA | CWGTCTCAAGAGATCGGAAGAGCGTCGTGTAGGGAAAGAGTGT |
| ApeKI_ E | TCAAGTT | ACACTCTTTCCCTACACGACGCTCTTCCGATCTTCAAGTT | CWGAACTTGAAGATCGGAAGAGCGTCGTGTAGGGAAAGAGTGT |
| ApeKI_ E | GATCATA | ACACTCTTTCCCTACACGACGCTCTTCCGATCTGATCATA | CWGTATGATCAGATCGGAAGAGCGTCGTGTAGGGAAAGAGTGT |
| ApeKI_ E | GCATTGA | ACACTCTTTCCCTACACGACGCTCTTCCGATCTGCATTGA | CWGTCAATGCAGATCGGAAGAGCGTCGTGTAGGGAAAGAGTGT |
| ApeKI_ E | CAGGTAT | ACACTCTTTCCCTACACGACGCTCTTCCGATCTCAGGTAT | CWGATACCTGAGATCGGAAGAGCGTCGTGTAGGGAAAGAGTGT |
| ApeKI_ E | TGCAATA | ACACTCTTTCCCTACACGACGCTCTTCCGATCTTGCAATA | CWGTATTGCAAGATCGGAAGAGCGTCGTGTAGGGAAAGAGTGT |
| ApeKI_ E | ATATCGT | ACACTCTTTCCCTACACGACGCTCTTCCGATCTATATCGT | CWGACGATATAGATCGGAAGAGCGTCGTGTAGGGAAAGAGTGT |
| ApeKI_ E | AGTCTAT | ACACTCTTTCCCTACACGACGCTCTTCCGATCTAGTCTAT | CWGATAGACTAGATCGGAAGAGCGTCGTGTAGGGAAAGAGTGT |
| ApeKI_ E | GTCTGAA | ACACTCTTTCCCTACACGACGCTCTTCCGATCTGTCTGAA | CWGTTCAGACAGATCGGAAGAGCGTCGTGTAGGGAAAGAGTGT |
| ApeKI_ E | ATCAGTT | ACACTCTTTCCCTACACGACGCTCTTCCGATCTATCAGTT | CWGAACTGATAGATCGGAAGAGCGTCGTGTAGGGAAAGAGTGT |
| ApeKI_ E | CAGTTGA | ACACTCTTTCCCTACACGACGCTCTTCCGATCTCAGTTGA | CWGTCAACTGAGATCGGAAGAGCGTCGTGTAGGGAAAGAGTGT |
| ApeKI_ E | TGTGCAA | ACACTCTTTCCCTACACGACGCTCTTCCGATCTTGTGCAA | CWGTTGCACAAGATCGGAAGAGCGTCGTGTAGGGAAAGAGTGT |
| ApeKI_ E | CGACAGT | ACACTCTTTCCCTACACGACGCTCTTCCGATCTCGACAGT | CWGACTGTCGAGATCGGAAGAGCGTCGTGTAGGGAAAGAGTGT |
| ApeKI_ E | ACGTGTA | ACACTCTTTCCCTACACGACGCTCTTCCGATCTACGTGTA | CWGTACACGTAGATCGGAAGAGCGTCGTGTAGGGAAAGAGTGT |
| ApeKI_ E | GATGCAT | ACACTCTTTCCCTACACGACGCTCTTCCGATCTGATGCAT | CWGATGCATCAGATCGGAAGAGCGTCGTGTAGGGAAAGAGTGT |
| ApeKI_ E | CTAATGT | ACACTCTTTCCCTACACGACGCTCTTCCGATCTCTAATGT | CWGACATTAGAGATCGGAAGAGCGTCGTGTAGGGAAAGAGTGT |
| ApeKI_ E | GTCGATA | ACACTCTTTCCCTACACGACGCTCTTCCGATCTGTCGATA | CWGTATCGACAGATCGGAAGAGCGTCGTGTAGGGAAAGAGTGT |
| ApeKI_ E | TATACGT | ACACTCTTTCCCTACACGACGCTCTTCCGATCTTATACGT | CWGACGTATAAGATCGGAAGAGCGTCGTGTAGGGAAAGAGTGT |
| ApeKI_ E | GCGTAAT | ACACTCTTTCCCTACACGACGCTCTTCCGATCTGCGTAAT | CWGATTACGCAGATCGGAAGAGCGTCGTGTAGGGAAAGAGTGT |
| ApeKI_ E | AGCGTTA | ACACTCTTTCCCTACACGACGCTCTTCCGATCTAGCGTTA | CWGTAACGCTAGATCGGAAGAGCGTCGTGTAGGGAAAGAGTGT |
| ApeKI_ E | ATCCGGA | ACACTCTTTCCCTACACGACGCTCTTCCGATCTATCCGGA | CWGTCCGGATAGATCGGAAGAGCGTCGTGTAGGGAAAGAGTGT |
| ApeKI_ E | TCAGTAT | ACACTCTTTCCCTACACGACGCTCTTCCGATCTTCAGTAT | CWGATACTGAAGATCGGAAGAGCGTCGTGTAGGGAAAGAGTGT |
| ApeKI_ E | CAATGTT | ACACTCTTTCCCTACACGACGCTCTTCCGATCTCAATGTT | CWGAACATTGAGATCGGAAGAGCGTCGTGTAGGGAAAGAGTGT |
| ApeKI_ E | GTTACGA | ACACTCTTTCCCTACACGACGCTCTTCCGATCTGTTACGA | CWGTCGTAACAGATCGGAAGAGCGTCGTGTAGGGAAAGAGTGT |
| ApeKI_ E | TGCATAT | ACACTCTTTCCCTACACGACGCTCTTCCGATCTTGCATAT | CWGATATGCAAGATCGGAAGAGCGTCGTGTAGGGAAAGAGTGT |
| ApeKI_ E | CAAGAAGT | ACACTCTTTCCCTACACGACGCTCTTCCGATCTCAAGAAGT | CWGACTTCTTGAGATCGGAAGAGCGTCGTGTAGGGAAAGAGTGT |
| ApeKI_ E | GTCATGGT | ACACTCTTTCCCTACACGACGCTCTTCCGATCTGTCATGGT | CWGACCATGACAGATCGGAAGAGCGTCGTGTAGGGAAAGAGTGT |
| ApeKI_ E | AACAGTGA | ACACTCTTTCCCTACACGACGCTCTTCCGATCTAACAGTGA | CWGTCACTGTTAGATCGGAAGAGCGTCGTGTAGGGAAAGAGTGT |
| ApeKI_ E | GTGCAAGA | ACACTCTTTCCCTACACGACGCTCTTCCGATCTGTGCAAGA | CWGTCTTGCACAGATCGGAAGAGCGTCGTGTAGGGAAAGAGTGT |
| ApeKI_ E | CAATAGGA | ACACTCTTTCCCTACACGACGCTCTTCCGATCTCAATAGGA | CWGTCCTATTGAGATCGGAAGAGCGTCGTGTAGGGAAAGAGTGT |
| ApeKI_ E | TGCAGTGT | ACACTCTTTCCCTACACGACGCTCTTCCGATCTTGCAGTGT | CWGACACTGCAAGATCGGAAGAGCGTCGTGTAGGGAAAGAGTGT |
| ApeKI_ E | AGGCTAGA | ACACTCTTTCCCTACACGACGCTCTTCCGATCTAGGCTAGA | CWGTCTAGCCTAGATCGGAAGAGCGTCGTGTAGGGAAAGAGTGT |
| ApeKI_ E | CTAGTGGT | ACACTCTTTCCCTACACGACGCTCTTCCGATCTCTAGTGGT | CWGACCACTAGAGATCGGAAGAGCGTCGTGTAGGGAAAGAGTGT |
| ApeKI_ E | GCTAGTGT | ACACTCTTTCCCTACACGACGCTCTTCCGATCTGCTAGTGT | CWGACACTAGCAGATCGGAAGAGCGTCGTGTAGGGAAAGAGTGT |
| ApeKI_ E | AGTTGGCA | ACACTCTTTCCCTACACGACGCTCTTCCGATCTAGTTGGCA | CWGTGCCAACTAGATCGGAAGAGCGTCGTGTAGGGAAAGAGTGT |
| ApeKI_ E | TCGCAAGT | ACACTCTTTCCCTACACGACGCTCTTCCGATCTTCGCAAGT | CWGACTTGCGAAGATCGGAAGAGCGTCGTGTAGGGAAAGAGTGT |
| ApeKI_ E | CGATGTGT | ACACTCTTTCCCTACACGACGCTCTTCCGATCTCGATGTGT | CWGACACATCGAGATCGGAAGAGCGTCGTGTAGGGAAAGAGTGT |
| ApeKI_ E | AACGTAGA | ACACTCTTTCCCTACACGACGCTCTTCCGATCTAACGTAGA | CWGTCTACGTTAGATCGGAAGAGCGTCGTGTAGGGAAAGAGTGT |
| ApeKI_ E | CTCACGGA | ACACTCTTTCCCTACACGACGCTCTTCCGATCTCTCACGGA | CWGTCCGTGAGAGATCGGAAGAGCGTCGTGTAGGGAAAGAGTGT |
| ApeKI_ E | TAGCGTGT | ACACTCTTTCCCTACACGACGCTCTTCCGATCTTAGCGTGT | CWGACACGCTAAGATCGGAAGAGCGTCGTGTAGGGAAAGAGTGT |
| ApeKI_ E | ACGTAAGA | ACACTCTTTCCCTACACGACGCTCTTCCGATCTACGTAAGA | CWGTCTTACGTAGATCGGAAGAGCGTCGTGTAGGGAAAGAGTGT |
| ApeKI_ E | CGTATGGT | ACACTCTTTCCCTACACGACGCTCTTCCGATCTCGTATGGT | CWGACCATACGAGATCGGAAGAGCGTCGTGTAGGGAAAGAGTGT |
| ApeKI_ E | GTACGTGT | ACACTCTTTCCCTACACGACGCTCTTCCGATCTGTACGTGT | CWGACACGTACAGATCGGAAGAGCGTCGTGTAGGGAAAGAGTGT |
| ApeKI_ E | TTCGAAGA | ACACTCTTTCCCTACACGACGCTCTTCCGATCTTTCGAAGA | CWGTCTTCGAAAGATCGGAAGAGCGTCGTGTAGGGAAAGAGTGT |
| ApeKI_ E | AATACGGA | ACACTCTTTCCCTACACGACGCTCTTCCGATCTAATACGGA | CWGTCCGTATTAGATCGGAAGAGCGTCGTGTAGGGAAAGAGTGT |
| ApeKI_ E | TGACTGGT | ACACTCTTTCCCTACACGACGCTCTTCCGATCTTGACTGGT | CWGACCAGTCAAGATCGGAAGAGCGTCGTGTAGGGAAAGAGTGT |
| ApeKI_ E | GCGGATGT | ACACTCTTTCCCTACACGACGCTCTTCCGATCTGCGGATGT | CWGACATCCGCAGATCGGAAGAGCGTCGTGTAGGGAAAGAGTGT |
| ApeKI_ E | CATTGAGA | ACACTCTTTCCCTACACGACGCTCTTCCGATCTCATTGAGA | CWGTCTCAATGAGATCGGAAGAGCGTCGTGTAGGGAAAGAGTGT |
| ApeKI_ E | GTAACAGA | ACACTCTTTCCCTACACGACGCTCTTCCGATCTGTAACAGA | CWGTCTGTTACAGATCGGAAGAGCGTCGTGTAGGGAAAGAGTGT |
| ApeKI_ E | AGCTTGGT | ACACTCTTTCCCTACACGACGCTCTTCCGATCTAGCTTGGT | CWGACCAAGCTAGATCGGAAGAGCGTCGTGTAGGGAAAGAGTGT |
| ApeKI_ E | ACAGATGA | ACACTCTTTCCCTACACGACGCTCTTCCGATCTACAGATGA | CWGTCATCTGTAGATCGGAAGAGCGTCGTGTAGGGAAAGAGTGT |
| ApeKI_ E | CAGTTGGT | ACACTCTTTCCCTACACGACGCTCTTCCGATCTCAGTTGGT | CWGACCAACTGAGATCGGAAGAGCGTCGTGTAGGGAAAGAGTGT |
| ApeKI_ E | TGCAAGAA | ACACTCTTTCCCTACACGACGCTCTTCCGATCTTGCAAGAA | CWGTTCTTGCAAGATCGGAAGAGCGTCGTGTAGGGAAAGAGTGT |
| ApeKI_ E | ACTCGAGA | ACACTCTTTCCCTACACGACGCTCTTCCGATCTACTCGAGA | CWGTCTCGAGTAGATCGGAAGAGCGTCGTGTAGGGAAAGAGTGT |
| ApeKI_ E | GGAGCTGT | ACACTCTTTCCCTACACGACGCTCTTCCGATCTGGAGCTGT | CWGACAGCTCCAGATCGGAAGAGCGTCGTGTAGGGAAAGAGTGT |
| ApeKI_ E | CTGAGTGT | ACACTCTTTCCCTACACGACGCTCTTCCGATCTCTGAGTGT | CWGACACTCAGAGATCGGAAGAGCGTCGTGTAGGGAAAGAGTGT |
| ApeKI_ E | GATCAGAA | ACACTCTTTCCCTACACGACGCTCTTCCGATCTGATCAGAA | CWGTTCTGATCAGATCGGAAGAGCGTCGTGTAGGGAAAGAGTGT |
| ApeKI_ E | TGCATAGA | ACACTCTTTCCCTACACGACGCTCTTCCGATCTTGCATAGA | CWGTCTATGCAAGATCGGAAGAGCGTCGTGTAGGGAAAGAGTGT |
| ApeKI_ E | GAACGAAT | ACACTCTTTCCCTACACGACGCTCTTCCGATCTGAACGAAT | CWGATTCGTTCAGATCGGAAGAGCGTCGTGTAGGGAAAGAGTGT |
| ApeKI_ E | TTGGCGGA | ACACTCTTTCCCTACACGACGCTCTTCCGATCTTTGGCGGA | CWGTCCGCCAAAGATCGGAAGAGCGTCGTGTAGGGAAAGAGTGT |
| ApeKI_ E | CGCCGCAT | ACACTCTTTCCCTACACGACGCTCTTCCGATCTCGCCGCAT | CWGATGCGGCGAGATCGGAAGAGCGTCGTGTAGGGAAAGAGTGT |
| ApeKI_ F | CGAA | ACACTCTTTCCCTACACGACGCTCTTCCGATCTCGAA | CWGTTCGAGATCGGAAGAGCGTCGTGTAGGGAAAGAGTGT |
| ApeKI_ F | GTCC | ACACTCTTTCCCTACACGACGCTCTTCCGATCTGTCC | CWGGGACAGATCGGAAGAGCGTCGTGTAGGGAAAGAGTGT |
| ApeKI_ F | ACAT | ACACTCTTTCCCTACACGACGCTCTTCCGATCTACAT | CWGATGTAGATCGGAAGAGCGTCGTGTAGGGAAAGAGTGT |
| ApeKI_ F | GCACT | ACACTCTTTCCCTACACGACGCTCTTCCGATCTGCACT | CWGAGTGCAGATCGGAAGAGCGTCGTGTAGGGAAAGAGTGT |
| ApeKI_ F | AGCTA | ACACTCTTTCCCTACACGACGCTCTTCCGATCTAGCTA | CWGTAGCTAGATCGGAAGAGCGTCGTGTAGGGAAAGAGTGT |
| ApeKI_ F | CAGCT | ACACTCTTTCCCTACACGACGCTCTTCCGATCTCAGCT | CWGAGCTGAGATCGGAAGAGCGTCGTGTAGGGAAAGAGTGT |
| ApeKI_ F | TATCTGA | ACACTCTTTCCCTACACGACGCTCTTCCGATCTTATCTGA | CWGTCAGATAAGATCGGAAGAGCGTCGTGTAGGGAAAGAGTGT |
| ApeKI_ F | TTCAAGT | ACACTCTTTCCCTACACGACGCTCTTCCGATCTTTCAAGT | CWGACTTGAAAGATCGGAAGAGCGTCGTGTAGGGAAAGAGTGT |
| ApeKI_ F | GAGCAGT | ACACTCTTTCCCTACACGACGCTCTTCCGATCTGAGCAGT | CWGACTGCTCAGATCGGAAGAGCGTCGTGTAGGGAAAGAGTGT |
| ApeKI_ F | TAACGAGA | ACACTCTTTCCCTACACGACGCTCTTCCGATCTTAACGAGA | CWGTCTCGTTAAGATCGGAAGAGCGTCGTGTAGGGAAAGAGTGT |
| ApeKI_ F | ATACAGGA | ACACTCTTTCCCTACACGACGCTCTTCCGATCTATACAGGA | CWGTCCTGTATAGATCGGAAGAGCGTCGTGTAGGGAAAGAGTGT |
| ApeKI_ F | CACAGAGT | ACACTCTTTCCCTACACGACGCTCTTCCGATCTCACAGAGT | CWGACTCTGTGAGATCGGAAGAGCGTCGTGTAGGGAAAGAGTGT |
| ApeKI_ F | TAGTGC | ACACTCTTTCCCTACACGACGCTCTTCCGATCTTAGTGC | CWGGCACTAAGATCGGAAGAGCGTCGTGTAGGGAAAGAGTGT |
| ApeKI_ F | GTTCCA | ACACTCTTTCCCTACACGACGCTCTTCCGATCTGTTCCA | CWGTGGAACAGATCGGAAGAGCGTCGTGTAGGGAAAGAGTGT |
| ApeKI_ F | TGAATC | ACACTCTTTCCCTACACGACGCTCTTCCGATCTTGAATC | CWGGATTCAAGATCGGAAGAGCGTCGTGTAGGGAAAGAGTGT |
| ApeKI_ F | CGCCAT | ACACTCTTTCCCTACACGACGCTCTTCCGATCTCGCCAT | CWGATGGCGAGATCGGAAGAGCGTCGTGTAGGGAAAGAGTGT |
| ApeKI_ F | ATCGTC | ACACTCTTTCCCTACACGACGCTCTTCCGATCTATCGTC | CWGGACGATAGATCGGAAGAGCGTCGTGTAGGGAAAGAGTGT |
| ApeKI_ F | GTCACT | ACACTCTTTCCCTACACGACGCTCTTCCGATCTGTCACT | CWGAGTGACAGATCGGAAGAGCGTCGTGTAGGGAAAGAGTGT |
| ApeKI_ F | CATCGC | ACACTCTTTCCCTACACGACGCTCTTCCGATCTCATCGC | CWGGCGATGAGATCGGAAGAGCGTCGTGTAGGGAAAGAGTGT |
| ApeKI_ F | AGGTCT | ACACTCTTTCCCTACACGACGCTCTTCCGATCTAGGTCT | CWGAGACCTAGATCGGAAGAGCGTCGTGTAGGGAAAGAGTGT |
| ApeKI_ F | CCTGCA | ACACTCTTTCCCTACACGACGCTCTTCCGATCTCCTGCA | CWGTGCAGGAGATCGGAAGAGCGTCGTGTAGGGAAAGAGTGT |
| ApeKI_ F | GTACTC | ACACTCTTTCCCTACACGACGCTCTTCCGATCTGTACTC | CWGGAGTACAGATCGGAAGAGCGTCGTGTAGGGAAAGAGTGT |
| ApeKI_ F | ACCTGC | ACACTCTTTCCCTACACGACGCTCTTCCGATCTACCTGC | CWGGCAGGTAGATCGGAAGAGCGTCGTGTAGGGAAAGAGTGT |
| ApeKI_ F | CTTGAC | ACACTCTTTCCCTACACGACGCTCTTCCGATCTCTTGAC | CWGGTCAAGAGATCGGAAGAGCGTCGTGTAGGGAAAGAGTGT |
| ApeKI_ F | CAGCGTA | ACACTCTTTCCCTACACGACGCTCTTCCGATCTCAGCGTA | CWGTACGCTGAGATCGGAAGAGCGTCGTGTAGGGAAAGAGTGT |
| ApeKI_ F | AGTTCGA | ACACTCTTTCCCTACACGACGCTCTTCCGATCTAGTTCGA | CWGTCGAACTAGATCGGAAGAGCGTCGTGTAGGGAAAGAGTGT |
| ApeKI_ F | GCTAATT | ACACTCTTTCCCTACACGACGCTCTTCCGATCTGCTAATT | CWGAATTAGCAGATCGGAAGAGCGTCGTGTAGGGAAAGAGTGT |
| ApeKI_ F | ACGCTGA | ACACTCTTTCCCTACACGACGCTCTTCCGATCTACGCTGA | CWGTCAGCGTAGATCGGAAGAGCGTCGTGTAGGGAAAGAGTGT |
| ApeKI_ F | TAAGCTT | ACACTCTTTCCCTACACGACGCTCTTCCGATCTTAAGCTT | CWGAAGCTTAAGATCGGAAGAGCGTCGTGTAGGGAAAGAGTGT |
| ApeKI_ F | CTTAGAT | ACACTCTTTCCCTACACGACGCTCTTCCGATCTCTTAGAT | CWGATCTAAGAGATCGGAAGAGCGTCGTGTAGGGAAAGAGTGT |
| ApeKI_ F | TGATACA | ACACTCTTTCCCTACACGACGCTCTTCCGATCTTGATACA | CWGTGTATCAAGATCGGAAGAGCGTCGTGTAGGGAAAGAGTGT |
| ApeKI_ F | ACCGAGT | ACACTCTTTCCCTACACGACGCTCTTCCGATCTACCGAGT | CWGACTCGGTAGATCGGAAGAGCGTCGTGTAGGGAAAGAGTGT |
| ApeKI_ F | GTACTTA | ACACTCTTTCCCTACACGACGCTCTTCCGATCTGTACTTA | CWGTAAGTACAGATCGGAAGAGCGTCGTGTAGGGAAAGAGTGT |
| ApeKI_ F | ATGTCAA | ACACTCTTTCCCTACACGACGCTCTTCCGATCTATGTCAA | CWGTTGACATAGATCGGAAGAGCGTCGTGTAGGGAAAGAGTGT |
| ApeKI_ F | CACAGGT | ACACTCTTTCCCTACACGACGCTCTTCCGATCTCACAGGT | CWGACCTGTGAGATCGGAAGAGCGTCGTGTAGGGAAAGAGTGT |
| ApeKI_ F | GGTGGCA | ACACTCTTTCCCTACACGACGCTCTTCCGATCTGGTGGCA | CWGTGCCACCAGATCGGAAGAGCGTCGTGTAGGGAAAGAGTGT |
| ApeKI_ F | CTGAATT | ACACTCTTTCCCTACACGACGCTCTTCCGATCTCTGAATT | CWGAATTCAGAGATCGGAAGAGCGTCGTGTAGGGAAAGAGTGT |
| ApeKI_ F | TCGTTAA | ACACTCTTTCCCTACACGACGCTCTTCCGATCTTCGTTAA | CWGTTAACGAAGATCGGAAGAGCGTCGTGTAGGGAAAGAGTGT |
| ApeKI_ F | TGATCAT | ACACTCTTTCCCTACACGACGCTCTTCCGATCTTGATCAT | CWGATGATCAAGATCGGAAGAGCGTCGTGTAGGGAAAGAGTGT |
| ApeKI_ F | CGTCGGA | ACACTCTTTCCCTACACGACGCTCTTCCGATCTCGTCGGA | CWGTCCGACGAGATCGGAAGAGCGTCGTGTAGGGAAAGAGTGT |
| ApeKI_ F | GACTATT | ACACTCTTTCCCTACACGACGCTCTTCCGATCTGACTATT | CWGAATAGTCAGATCGGAAGAGCGTCGTGTAGGGAAAGAGTGT |
| ApeKI_ F | TAGGTCA | ACACTCTTTCCCTACACGACGCTCTTCCGATCTTAGGTCA | CWGTGACCTAAGATCGGAAGAGCGTCGTGTAGGGAAAGAGTGT |
| ApeKI_ F | GCAACGT | ACACTCTTTCCCTACACGACGCTCTTCCGATCTGCAACGT | CWGACGTTGCAGATCGGAAGAGCGTCGTGTAGGGAAAGAGTGT |
| ApeKI_ F | ACTTGAT | ACACTCTTTCCCTACACGACGCTCTTCCGATCTACTTGAT | CWGATCAAGTAGATCGGAAGAGCGTCGTGTAGGGAAAGAGTGT |
| ApeKI_ F | TCTGATA | ACACTCTTTCCCTACACGACGCTCTTCCGATCTTCTGATA | CWGTATCAGAAGATCGGAAGAGCGTCGTGTAGGGAAAGAGTGT |
| ApeKI_ F | ATAGTCA | ACACTCTTTCCCTACACGACGCTCTTCCGATCTATAGTCA | CWGTGACTATAGATCGGAAGAGCGTCGTGTAGGGAAAGAGTGT |
| ApeKI_ F | TAGCCAT | ACACTCTTTCCCTACACGACGCTCTTCCGATCTTAGCCAT | CWGATGGCTAAGATCGGAAGAGCGTCGTGTAGGGAAAGAGTGT |
| ApeKI_ F | AGGAGTC | ACACTCTTTCCCTACACGACGCTCTTCCGATCTAGGAGTC | CWGGACTCCTAGATCGGAAGAGCGTCGTGTAGGGAAAGAGTGT |
| ApeKI_ F | GTAGAGC | ACACTCTTTCCCTACACGACGCTCTTCCGATCTGTAGAGC | CWGGCTCTACAGATCGGAAGAGCGTCGTGTAGGGAAAGAGTGT |
| ApeKI_ F | GACCTAT | ACACTCTTTCCCTACACGACGCTCTTCCGATCTGACCTAT | CWGATAGGTCAGATCGGAAGAGCGTCGTGTAGGGAAAGAGTGT |
| ApeKI_ F | CATTAGT | ACACTCTTTCCCTACACGACGCTCTTCCGATCTCATTAGT | CWGACTAATGAGATCGGAAGAGCGTCGTGTAGGGAAAGAGTGT |
| ApeKI_ F | ATTAGCA | ACACTCTTTCCCTACACGACGCTCTTCCGATCTATTAGCA | CWGTGCTAATAGATCGGAAGAGCGTCGTGTAGGGAAAGAGTGT |
| ApeKI_ F | GAATCTA | ACACTCTTTCCCTACACGACGCTCTTCCGATCTGAATCTA | CWGTAGATTCAGATCGGAAGAGCGTCGTGTAGGGAAAGAGTGT |
| ApeKI_ F | TGTCATT | ACACTCTTTCCCTACACGACGCTCTTCCGATCTTGTCATT | CWGAATGACAAGATCGGAAGAGCGTCGTGTAGGGAAAGAGTGT |
| ApeKI_ F | GCCAGAT | ACACTCTTTCCCTACACGACGCTCTTCCGATCTGCCAGAT | CWGATCTGGCAGATCGGAAGAGCGTCGTGTAGGGAAAGAGTGT |
| ApeKI_ F | CCAGGTA | ACACTCTTTCCCTACACGACGCTCTTCCGATCTCCAGGTA | CWGTACCTGGAGATCGGAAGAGCGTCGTGTAGGGAAAGAGTGT |
| ApeKI_ F | TGGCAAC | ACACTCTTTCCCTACACGACGCTCTTCCGATCTTGGCAAC | CWGGTTGCCAAGATCGGAAGAGCGTCGTGTAGGGAAAGAGTGT |
| ApeKI_ F | ATGATCT | ACACTCTTTCCCTACACGACGCTCTTCCGATCTATGATCT | CWGAGATCATAGATCGGAAGAGCGTCGTGTAGGGAAAGAGTGT |
| ApeKI_ F | CATGTTA | ACACTCTTTCCCTACACGACGCTCTTCCGATCTCATGTTA | CWGTAACATGAGATCGGAAGAGCGTCGTGTAGGGAAAGAGTGT |
| ApeKI_ F | TGTAAGC | ACACTCTTTCCCTACACGACGCTCTTCCGATCTTGTAAGC | CWGGCTTACAAGATCGGAAGAGCGTCGTGTAGGGAAAGAGTGT |
| ApeKI_ F | ACGTGTGT | ACACTCTTTCCCTACACGACGCTCTTCCGATCTACGTGTGT | CWGACACACGTAGATCGGAAGAGCGTCGTGTAGGGAAAGAGTGT |
| ApeKI_ F | CAAGTGAA | ACACTCTTTCCCTACACGACGCTCTTCCGATCTCAAGTGAA | CWGTTCACTTGAGATCGGAAGAGCGTCGTGTAGGGAAAGAGTGT |
| ApeKI_ F | GTTAACGA | ACACTCTTTCCCTACACGACGCTCTTCCGATCTGTTAACGA | CWGTCGTTAACAGATCGGAAGAGCGTCGTGTAGGGAAAGAGTGT |
| ApeKI_ F | TCGTGGAT | ACACTCTTTCCCTACACGACGCTCTTCCGATCTTCGTGGAT | CWGATCCACGAAGATCGGAAGAGCGTCGTGTAGGGAAAGAGTGT |
| ApeKI_ F | AGCGATAA | ACACTCTTTCCCTACACGACGCTCTTCCGATCTAGCGATAA | CWGTTATCGCTAGATCGGAAGAGCGTCGTGTAGGGAAAGAGTGT |
| ApeKI_ F | CCACCAGT | ACACTCTTTCCCTACACGACGCTCTTCCGATCTCCACCAGT | CWGACTGGTGGAGATCGGAAGAGCGTCGTGTAGGGAAAGAGTGT |
| ApeKI_ F | GGCGTGTA | ACACTCTTTCCCTACACGACGCTCTTCCGATCTGGCGTGTA | CWGTACACGCCAGATCGGAAGAGCGTCGTGTAGGGAAAGAGTGT |
| ApeKI_ F | AAGACAGT | ACACTCTTTCCCTACACGACGCTCTTCCGATCTAAGACAGT | CWGACTGTCTTAGATCGGAAGAGCGTCGTGTAGGGAAAGAGTGT |
| ApeKI_ F | TTGTGCGA | ACACTCTTTCCCTACACGACGCTCTTCCGATCTTTGTGCGA | CWGTCGCACAAAGATCGGAAGAGCGTCGTGTAGGGAAAGAGTGT |
| ApeKI_ F | GAAGACAT | ACACTCTTTCCCTACACGACGCTCTTCCGATCTGAAGACAT | CWGATGTCTTCAGATCGGAAGAGCGTCGTGTAGGGAAAGAGTGT |
| ApeKI_ F | TGTCAAGA | ACACTCTTTCCCTACACGACGCTCTTCCGATCTTGTCAAGA | CWGTCTTGACAAGATCGGAAGAGCGTCGTGTAGGGAAAGAGTGT |
| ApeKI_ F | GCGAGGTT | ACACTCTTTCCCTACACGACGCTCTTCCGATCTGCGAGGTT | CWGAACCTCGCAGATCGGAAGAGCGTCGTGTAGGGAAAGAGTGT |
| ApeKI_ F | CGCTTGAA | ACACTCTTTCCCTACACGACGCTCTTCCGATCTCGCTTGAA | CWGTTCAAGCGAGATCGGAAGAGCGTCGTGTAGGGAAAGAGTGT |
| ApeKI_ F | ATCGGTGT | ACACTCTTTCCCTACACGACGCTCTTCCGATCTATCGGTGT | CWGACACCGATAGATCGGAAGAGCGTCGTGTAGGGAAAGAGTGT |
| ApeKI_ F | CCTACCGA | ACACTCTTTCCCTACACGACGCTCTTCCGATCTCCTACCGA | CWGTCGGTAGGAGATCGGAAGAGCGTCGTGTAGGGAAAGAGTGT |
| ApeKI_ F | GGACAATA | ACACTCTTTCCCTACACGACGCTCTTCCGATCTGGACAATA | CWGTATTGTCCAGATCGGAAGAGCGTCGTGTAGGGAAAGAGTGT |
| ApeKI_ F | AACGGAAT | ACACTCTTTCCCTACACGACGCTCTTCCGATCTAACGGAAT | CWGATTCCGTTAGATCGGAAGAGCGTCGTGTAGGGAAAGAGTGT |
| ApeKI_ F | TCGATGGT | ACACTCTTTCCCTACACGACGCTCTTCCGATCTTCGATGGT | CWGACCATCGAAGATCGGAAGAGCGTCGTGTAGGGAAAGAGTGT |
| ApeKI_ F | GTTCATTA | ACACTCTTTCCCTACACGACGCTCTTCCGATCTGTTCATTA | CWGTAATGAACAGATCGGAAGAGCGTCGTGTAGGGAAAGAGTGT |
| ApeKI_ F | GAATCGAA | ACACTCTTTCCCTACACGACGCTCTTCCGATCTGAATCGAA | CWGTTCGATTCAGATCGGAAGAGCGTCGTGTAGGGAAAGAGTGT |
| ApeKI_ F | CATGGTGT | ACACTCTTTCCCTACACGACGCTCTTCCGATCTCATGGTGT | CWGACACCATGAGATCGGAAGAGCGTCGTGTAGGGAAAGAGTGT |
| ApeKI_ F | TGGCTATT | ACACTCTTTCCCTACACGACGCTCTTCCGATCTTGGCTATT | CWGAATAGCCAAGATCGGAAGAGCGTCGTGTAGGGAAAGAGTGT |
| ApeKI_ F | ACCACCGT | ACACTCTTTCCCTACACGACGCTCTTCCGATCTACCACCGT | CWGACGGTGGTAGATCGGAAGAGCGTCGTGTAGGGAAAGAGTGT |
| ApeKI_ F | CTATGTAA | ACACTCTTTCCCTACACGACGCTCTTCCGATCTCTATGTAA | CWGTTACATAGAGATCGGAAGAGCGTCGTGTAGGGAAAGAGTGT |
| ApeKI_ F | AACGAGTA | ACACTCTTTCCCTACACGACGCTCTTCCGATCTAACGAGTA | CWGTACTCGTTAGATCGGAAGAGCGTCGTGTAGGGAAAGAGTGT |
| ApeKI_ F | CCGACCAT | ACACTCTTTCCCTACACGACGCTCTTCCGATCTCCGACCAT | CWGATGGTCGGAGATCGGAAGAGCGTCGTGTAGGGAAAGAGTGT |
| ApeKI_ F | TGTCTTAA | ACACTCTTTCCCTACACGACGCTCTTCCGATCTTGTCTTAA | CWGTTAAGACAAGATCGGAAGAGCGTCGTGTAGGGAAAGAGTGT |
| ApeKI_ F | GTATACTA | ACACTCTTTCCCTACACGACGCTCTTCCGATCTGTATACTA | CWGTAGTATACAGATCGGAAGAGCGTCGTGTAGGGAAAGAGTGT |
| ApeKI_ F | TGTGCAGT | ACACTCTTTCCCTACACGACGCTCTTCCGATCTTGTGCAGT | CWGACTGCACAAGATCGGAAGAGCGTCGTGTAGGGAAAGAGTGT |
| ApeKI_ F | ATGCTGGT | ACACTCTTTCCCTACACGACGCTCTTCCGATCTATGCTGGT | CWGACCAGCATAGATCGGAAGAGCGTCGTGTAGGGAAAGAGTGT |
| ApeKI_ F | GCAAGTAA | ACACTCTTTCCCTACACGACGCTCTTCCGATCTGCAAGTAA | CWGTTACTTGCAGATCGGAAGAGCGTCGTGTAGGGAAAGAGTGT |
| ApeKI_ F | CAGGAATA | ACACTCTTTCCCTACACGACGCTCTTCCGATCTCAGGAATA | CWGTATTCCTGAGATCGGAAGAGCGTCGTGTAGGGAAAGAGTGT |
| ApeKI_ F | GTCTGGAT | ACACTCTTTCCCTACACGACGCTCTTCCGATCTGTCTGGAT | CWGATCCAGACAGATCGGAAGAGCGTCGTGTAGGGAAAGAGTGT |
| ApeKI_ F | CACCACGT | ACACTCTTTCCCTACACGACGCTCTTCCGATCTCACCACGT | CWGACGTGGTGAGATCGGAAGAGCGTCGTGTAGGGAAAGAGTGT |
| ApeKI_ F | TCATTGTA | ACACTCTTTCCCTACACGACGCTCTTCCGATCTTCATTGTA | CWGTACAATGAAGATCGGAAGAGCGTCGTGTAGGGAAAGAGTGT |
| ApeKI_ F | GGTTCATT | ACACTCTTTCCCTACACGACGCTCTTCCGATCTGGTTCATT | CWGAATGAACCAGATCGGAAGAGCGTCGTGTAGGGAAAGAGTGT |
| ApeKI_ G | GCGA | ACACTCTTTCCCTACACGACGCTCTTCCGATCTGCGA | CWGTCGCAGATCGGAAGAGCGTCGTGTAGGGAAAGAGTGT |
| ApeKI_ G | TGCT | ACACTCTTTCCCTACACGACGCTCTTCCGATCTTGCT | CWGAGCAAGATCGGAAGAGCGTCGTGTAGGGAAAGAGTGT |
| ApeKI_ G | CCTC | ACACTCTTTCCCTACACGACGCTCTTCCGATCTCCTC | CWGGAGGAGATCGGAAGAGCGTCGTGTAGGGAAAGAGTGT |
| ApeKI_ G | TCAGC | ACACTCTTTCCCTACACGACGCTCTTCCGATCTTCAGC | CWGGCTGAAGATCGGAAGAGCGTCGTGTAGGGAAAGAGTGT |
| ApeKI_ G | CATGC | ACACTCTTTCCCTACACGACGCTCTTCCGATCTCATGC | CWGGCATGAGATCGGAAGAGCGTCGTGTAGGGAAAGAGTGT |
| ApeKI_ G | GCTAC | ACACTCTTTCCCTACACGACGCTCTTCCGATCTGCTAC | CWGGTAGCAGATCGGAAGAGCGTCGTGTAGGGAAAGAGTGT |
| ApeKI_ G | CTGAC | ACACTCTTTCCCTACACGACGCTCTTCCGATCTCTGAC | CWGGTCAGAGATCGGAAGAGCGTCGTGTAGGGAAAGAGTGT |
| ApeKI_ G | TCTAGGA | ACACTCTTTCCCTACACGACGCTCTTCCGATCTTCTAGGA | CWGTCCTAGAAGATCGGAAGAGCGTCGTGTAGGGAAAGAGTGT |
| ApeKI_ G | ACACGGT | ACACTCTTTCCCTACACGACGCTCTTCCGATCTACACGGT | CWGACCGTGTAGATCGGAAGAGCGTCGTGTAGGGAAAGAGTGT |
| ApeKI_ G | GACGTGA | ACACTCTTTCCCTACACGACGCTCTTCCGATCTGACGTGA | CWGTCACGTCAGATCGGAAGAGCGTCGTGTAGGGAAAGAGTGT |
| ApeKI_ G | CGGCAGGT | ACACTCTTTCCCTACACGACGCTCTTCCGATCTCGGCAGGT | CWGACCTGCCGAGATCGGAAGAGCGTCGTGTAGGGAAAGAGTGT |
| ApeKI_ G | GCGCGTGA | ACACTCTTTCCCTACACGACGCTCTTCCGATCTGCGCGTGA | CWGTCACGCGCAGATCGGAAGAGCGTCGTGTAGGGAAAGAGTGT |
| ApeKI_ G | ACCGCT | ACACTCTTTCCCTACACGACGCTCTTCCGATCTACCGCT | CWGAGCGGTAGATCGGAAGAGCGTCGTGTAGGGAAAGAGTGT |
| ApeKI_ G | GCTCAC | ACACTCTTTCCCTACACGACGCTCTTCCGATCTGCTCAC | CWGGTGAGCAGATCGGAAGAGCGTCGTGTAGGGAAAGAGTGT |
| ApeKI_ G | CTATGC | ACACTCTTTCCCTACACGACGCTCTTCCGATCTCTATGC | CWGGCATAGAGATCGGAAGAGCGTCGTGTAGGGAAAGAGTGT |
| ApeKI_ G | CAGCCT | ACACTCTTTCCCTACACGACGCTCTTCCGATCTCAGCCT | CWGAGGCTGAGATCGGAAGAGCGTCGTGTAGGGAAAGAGTGT |
| ApeKI_ G | GCCATC | ACACTCTTTCCCTACACGACGCTCTTCCGATCTGCCATC | CWGGATGGCAGATCGGAAGAGCGTCGTGTAGGGAAAGAGTGT |
| ApeKI_ G | CGCTCA | ACACTCTTTCCCTACACGACGCTCTTCCGATCTCGCTCA | CWGTGAGCGAGATCGGAAGAGCGTCGTGTAGGGAAAGAGTGT |
| ApeKI_ G | ACGCTC | ACACTCTTTCCCTACACGACGCTCTTCCGATCTACGCTC | CWGGAGCGTAGATCGGAAGAGCGTCGTGTAGGGAAAGAGTGT |
| ApeKI_ G | CTGATC | ACACTCTTTCCCTACACGACGCTCTTCCGATCTCTGATC | CWGGATCAGAGATCGGAAGAGCGTCGTGTAGGGAAAGAGTGT |
| ApeKI_ G | CCAGTC | ACACTCTTTCCCTACACGACGCTCTTCCGATCTCCAGTC | CWGGACTGGAGATCGGAAGAGCGTCGTGTAGGGAAAGAGTGT |
| ApeKI_ G | TACGCC | ACACTCTTTCCCTACACGACGCTCTTCCGATCTTACGCC | CWGGGCGTAAGATCGGAAGAGCGTCGTGTAGGGAAAGAGTGT |
| ApeKI_ G | CCGTAC | ACACTCTTTCCCTACACGACGCTCTTCCGATCTCCGTAC | CWGGTACGGAGATCGGAAGAGCGTCGTGTAGGGAAAGAGTGT |
| ApeKI_ G | TCGACC | ACACTCTTTCCCTACACGACGCTCTTCCGATCTTCGACC | CWGGGTCGAAGATCGGAAGAGCGTCGTGTAGGGAAAGAGTGT |
| ApeKI_ G | ACGGCAT | ACACTCTTTCCCTACACGACGCTCTTCCGATCTACGGCAT | CWGATGCCGTAGATCGGAAGAGCGTCGTGTAGGGAAAGAGTGT |
| ApeKI_ G | TTCTTGA | ACACTCTTTCCCTACACGACGCTCTTCCGATCTTTCTTGA | CWGTCAAGAAAGATCGGAAGAGCGTCGTGTAGGGAAAGAGTGT |
| ApeKI_ G | GGACCTT | ACACTCTTTCCCTACACGACGCTCTTCCGATCTGGACCTT | CWGAAGGTCCAGATCGGAAGAGCGTCGTGTAGGGAAAGAGTGT |
| ApeKI_ G | GTGTACA | ACACTCTTTCCCTACACGACGCTCTTCCGATCTGTGTACA | CWGTGTACACAGATCGGAAGAGCGTCGTGTAGGGAAAGAGTGT |
| ApeKI_ G | TAGCGGC | ACACTCTTTCCCTACACGACGCTCTTCCGATCTTAGCGGC | CWGGCCGCTAAGATCGGAAGAGCGTCGTGTAGGGAAAGAGTGT |
| ApeKI_ G | CGCTTAA | ACACTCTTTCCCTACACGACGCTCTTCCGATCTCGCTTAA | CWGTTAAGCGAGATCGGAAGAGCGTCGTGTAGGGAAAGAGTGT |
| ApeKI_ G | GATAGCT | ACACTCTTTCCCTACACGACGCTCTTCCGATCTGATAGCT | CWGAGCTATCAGATCGGAAGAGCGTCGTGTAGGGAAAGAGTGT |
| ApeKI_ G | AGAGCCT | ACACTCTTTCCCTACACGACGCTCTTCCGATCTAGAGCCT | CWGAGGCTCTAGATCGGAAGAGCGTCGTGTAGGGAAAGAGTGT |
| ApeKI_ G | TACGAGC | ACACTCTTTCCCTACACGACGCTCTTCCGATCTTACGAGC | CWGGCTCGTAAGATCGGAAGAGCGTCGTGTAGGGAAAGAGTGT |
| ApeKI_ G | TTGACTA | ACACTCTTTCCCTACACGACGCTCTTCCGATCTTTGACTA | CWGTAGTCAAAGATCGGAAGAGCGTCGTGTAGGGAAAGAGTGT |
| ApeKI_ G | CGCCGAT | ACACTCTTTCCCTACACGACGCTCTTCCGATCTCGCCGAT | CWGATCGGCGAGATCGGAAGAGCGTCGTGTAGGGAAAGAGTGT |
| ApeKI_ G | AATTGGC | ACACTCTTTCCCTACACGACGCTCTTCCGATCTAATTGGC | CWGGCCAATTAGATCGGAAGAGCGTCGTGTAGGGAAAGAGTGT |
| ApeKI_ G | ATACTGC | ACACTCTTTCCCTACACGACGCTCTTCCGATCTATACTGC | CWGGCAGTATAGATCGGAAGAGCGTCGTGTAGGGAAAGAGTGT |
| ApeKI_ G | GCGGCTA | ACACTCTTTCCCTACACGACGCTCTTCCGATCTGCGGCTA | CWGTAGCCGCAGATCGGAAGAGCGTCGTGTAGGGAAAGAGTGT |
| ApeKI_ G | CGTTATA | ACACTCTTTCCCTACACGACGCTCTTCCGATCTCGTTATA | CWGTATAACGAGATCGGAAGAGCGTCGTGTAGGGAAAGAGTGT |
| ApeKI_ G | TTAGACT | ACACTCTTTCCCTACACGACGCTCTTCCGATCTTTAGACT | CWGAGTCTAAAGATCGGAAGAGCGTCGTGTAGGGAAAGAGTGT |
| ApeKI_ G | AGGTTAC | ACACTCTTTCCCTACACGACGCTCTTCCGATCTAGGTTAC | CWGGTAACCTAGATCGGAAGAGCGTCGTGTAGGGAAAGAGTGT |
| ApeKI_ G | GGCAACT | ACACTCTTTCCCTACACGACGCTCTTCCGATCTGGCAACT | CWGAGTTGCCAGATCGGAAGAGCGTCGTGTAGGGAAAGAGTGT |
| ApeKI_ G | TCACCGA | ACACTCTTTCCCTACACGACGCTCTTCCGATCTTCACCGA | CWGTCGGTGAAGATCGGAAGAGCGTCGTGTAGGGAAAGAGTGT |
| ApeKI_ G | AAGGTTC | ACACTCTTTCCCTACACGACGCTCTTCCGATCTAAGGTTC | CWGGAACCTTAGATCGGAAGAGCGTCGTGTAGGGAAAGAGTGT |
| ApeKI_ G | CTAGCAT | ACACTCTTTCCCTACACGACGCTCTTCCGATCTCTAGCAT | CWGATGCTAGAGATCGGAAGAGCGTCGTGTAGGGAAAGAGTGT |
| ApeKI_ G | GCCGACC | ACACTCTTTCCCTACACGACGCTCTTCCGATCTGCCGACC | CWGGGTCGGCAGATCGGAAGAGCGTCGTGTAGGGAAAGAGTGT |
| ApeKI_ G | AGTACTT | ACACTCTTTCCCTACACGACGCTCTTCCGATCTAGTACTT | CWGAAGTACTAGATCGGAAGAGCGTCGTGTAGGGAAAGAGTGT |
| ApeKI_ G | TAATTGC | ACACTCTTTCCCTACACGACGCTCTTCCGATCTTAATTGC | CWGGCAATTAAGATCGGAAGAGCGTCGTGTAGGGAAAGAGTGT |
| ApeKI_ G | GCTGGAC | ACACTCTTTCCCTACACGACGCTCTTCCGATCTGCTGGAC | CWGGTCCAGCAGATCGGAAGAGCGTCGTGTAGGGAAAGAGTGT |
| ApeKI_ G | GTCATCA | ACACTCTTTCCCTACACGACGCTCTTCCGATCTGTCATCA | CWGTGATGACAGATCGGAAGAGCGTCGTGTAGGGAAAGAGTGT |
| ApeKI_ G | CAGTGAC | ACACTCTTTCCCTACACGACGCTCTTCCGATCTCAGTGAC | CWGGTCACTGAGATCGGAAGAGCGTCGTGTAGGGAAAGAGTGT |
| ApeKI_ G | GCACACT | ACACTCTTTCCCTACACGACGCTCTTCCGATCTGCACACT | CWGAGTGTGCAGATCGGAAGAGCGTCGTGTAGGGAAAGAGTGT |
| ApeKI_ G | AAGTCTT | ACACTCTTTCCCTACACGACGCTCTTCCGATCTAAGTCTT | CWGAAGACTTAGATCGGAAGAGCGTCGTGTAGGGAAAGAGTGT |
| ApeKI_ G | TGTATCA | ACACTCTTTCCCTACACGACGCTCTTCCGATCTTGTATCA | CWGTGATACAAGATCGGAAGAGCGTCGTGTAGGGAAAGAGTGT |
| ApeKI_ G | CTGGAAC | ACACTCTTTCCCTACACGACGCTCTTCCGATCTCTGGAAC | CWGGTTCCAGAGATCGGAAGAGCGTCGTGTAGGGAAAGAGTGT |
| ApeKI_ G | ATTCGAC | ACACTCTTTCCCTACACGACGCTCTTCCGATCTATTCGAC | CWGGTCGAATAGATCGGAAGAGCGTCGTGTAGGGAAAGAGTGT |
| ApeKI_ G | CAAGATC | ACACTCTTTCCCTACACGACGCTCTTCCGATCTCAAGATC | CWGGATCTTGAGATCGGAAGAGCGTCGTGTAGGGAAAGAGTGT |
| ApeKI_ G | TCGCGCA | ACACTCTTTCCCTACACGACGCTCTTCCGATCTTCGCGCA | CWGTGCGCGAAGATCGGAAGAGCGTCGTGTAGGGAAAGAGTGT |
| ApeKI_ G | AGTGTGC | ACACTCTTTCCCTACACGACGCTCTTCCGATCTAGTGTGC | CWGGCACACTAGATCGGAAGAGCGTCGTGTAGGGAAAGAGTGT |
| ApeKI_ G | GAATTCT | ACACTCTTTCCCTACACGACGCTCTTCCGATCTGAATTCT | CWGAGAATTCAGATCGGAAGAGCGTCGTGTAGGGAAAGAGTGT |
| ApeKI_ G | AAGATCGA | ACACTCTTTCCCTACACGACGCTCTTCCGATCTAAGATCGA | CWGTCGATCTTAGATCGGAAGAGCGTCGTGTAGGGAAAGAGTGT |
| ApeKI_ G | CGAAGAAT | ACACTCTTTCCCTACACGACGCTCTTCCGATCTCGAAGAAT | CWGATTCTTCGAGATCGGAAGAGCGTCGTGTAGGGAAAGAGTGT |
| ApeKI_ G | TCTGATTA | ACACTCTTTCCCTACACGACGCTCTTCCGATCTTCTGATTA | CWGTAATCAGAAGATCGGAAGAGCGTCGTGTAGGGAAAGAGTGT |
| ApeKI_ G | ATCTCTGA | ACACTCTTTCCCTACACGACGCTCTTCCGATCTATCTCTGA | CWGTCAGAGATAGATCGGAAGAGCGTCGTGTAGGGAAAGAGTGT |
| ApeKI_ G | AAGCAGAT | ACACTCTTTCCCTACACGACGCTCTTCCGATCTAAGCAGAT | CWGATCTGCTTAGATCGGAAGAGCGTCGTGTAGGGAAAGAGTGT |
| ApeKI_ G | TGCGGATT | ACACTCTTTCCCTACACGACGCTCTTCCGATCTTGCGGATT | CWGAATCCGCAAGATCGGAAGAGCGTCGTGTAGGGAAAGAGTGT |
| ApeKI_ G | CTCCTCGA | ACACTCTTTCCCTACACGACGCTCTTCCGATCTCTCCTCGA | CWGTCGAGGAGAGATCGGAAGAGCGTCGTGTAGGGAAAGAGTGT |
| ApeKI_ G | GGTACTAT | ACACTCTTTCCCTACACGACGCTCTTCCGATCTGGTACTAT | CWGATAGTACCAGATCGGAAGAGCGTCGTGTAGGGAAAGAGTGT |
| ApeKI_ G | ACAGGATA | ACACTCTTTCCCTACACGACGCTCTTCCGATCTACAGGATA | CWGTATCCTGTAGATCGGAAGAGCGTCGTGTAGGGAAAGAGTGT |
| ApeKI_ G | GATCTGGT | ACACTCTTTCCCTACACGACGCTCTTCCGATCTGATCTGGT | CWGACCAGATCAGATCGGAAGAGCGTCGTGTAGGGAAAGAGTGT |
| ApeKI_ G | CTGAAGAA | ACACTCTTTCCCTACACGACGCTCTTCCGATCTCTGAAGAA | CWGTTCTTCAGAGATCGGAAGAGCGTCGTGTAGGGAAAGAGTGT |
| ApeKI_ G | TCCGCCAA | ACACTCTTTCCCTACACGACGCTCTTCCGATCTTCCGCCAA | CWGTTGGCGGAAGATCGGAAGAGCGTCGTGTAGGGAAAGAGTGT |
| ApeKI_ G | ATATTCGT | ACACTCTTTCCCTACACGACGCTCTTCCGATCTATATTCGT | CWGACGAATATAGATCGGAAGAGCGTCGTGTAGGGAAAGAGTGT |
| ApeKI_ G | CGGTGATT | ACACTCTTTCCCTACACGACGCTCTTCCGATCTCGGTGATT | CWGAATCACCGAGATCGGAAGAGCGTCGTGTAGGGAAAGAGTGT |
| ApeKI_ G | TATGCTGA | ACACTCTTTCCCTACACGACGCTCTTCCGATCTTATGCTGA | CWGTCAGCATAAGATCGGAAGAGCGTCGTGTAGGGAAAGAGTGT |
| ApeKI_ G | GAACAGTT | ACACTCTTTCCCTACACGACGCTCTTCCGATCTGAACAGTT | CWGAACTGTTCAGATCGGAAGAGCGTCGTGTAGGGAAAGAGTGT |
| ApeKI_ G | ACGATTAA | ACACTCTTTCCCTACACGACGCTCTTCCGATCTACGATTAA | CWGTTAATCGTAGATCGGAAGAGCGTCGTGTAGGGAAAGAGTGT |
| ApeKI_ G | GGTTACGT | ACACTCTTTCCCTACACGACGCTCTTCCGATCTGGTTACGT | CWGACGTAACCAGATCGGAAGAGCGTCGTGTAGGGAAAGAGTGT |
| ApeKI_ G | TTACGGTA | ACACTCTTTCCCTACACGACGCTCTTCCGATCTTTACGGTA | CWGTACCGTAAAGATCGGAAGAGCGTCGTGTAGGGAAAGAGTGT |
| ApeKI_ G | CACGCCAT | ACACTCTTTCCCTACACGACGCTCTTCCGATCTCACGCCAT | CWGATGGCGTGAGATCGGAAGAGCGTCGTGTAGGGAAAGAGTGT |
| ApeKI_ G | TCGAGATA | ACACTCTTTCCCTACACGACGCTCTTCCGATCTTCGAGATA | CWGTATCTCGAAGATCGGAAGAGCGTCGTGTAGGGAAAGAGTGT |
| ApeKI_ G | AGAACTGA | ACACTCTTTCCCTACACGACGCTCTTCCGATCTAGAACTGA | CWGTCAGTTCTAGATCGGAAGAGCGTCGTGTAGGGAAAGAGTGT |
| ApeKI_ G | GTTCTAAT | ACACTCTTTCCCTACACGACGCTCTTCCGATCTGTTCTAAT | CWGATTAGAACAGATCGGAAGAGCGTCGTGTAGGGAAAGAGTGT |
| ApeKI_ G | CCTTAGAA | ACACTCTTTCCCTACACGACGCTCTTCCGATCTCCTTAGAA | CWGTTCTAAGGAGATCGGAAGAGCGTCGTGTAGGGAAAGAGTGT |
| ApeKI_ G | GACTGTGT | ACACTCTTTCCCTACACGACGCTCTTCCGATCTGACTGTGT | CWGACACAGTCAGATCGGAAGAGCGTCGTGTAGGGAAAGAGTGT |
| ApeKI_ G | CGCGCCTA | ACACTCTTTCCCTACACGACGCTCTTCCGATCTCGCGCCTA | CWGTAGGCGCGAGATCGGAAGAGCGTCGTGTAGGGAAAGAGTGT |
| ApeKI_ G | TTGCATAT | ACACTCTTTCCCTACACGACGCTCTTCCGATCTTTGCATAT | CWGATATGCAAAGATCGGAAGAGCGTCGTGTAGGGAAAGAGTGT |
| ApeKI_ G | GCTATAGA | ACACTCTTTCCCTACACGACGCTCTTCCGATCTGCTATAGA | CWGTCTATAGCAGATCGGAAGAGCGTCGTGTAGGGAAAGAGTGT |
| ApeKI_ G | ACAGCCTT | ACACTCTTTCCCTACACGACGCTCTTCCGATCTACAGCCTT | CWGAAGGCTGTAGATCGGAAGAGCGTCGTGTAGGGAAAGAGTGT |
| ApeKI_ G | CTGTGGTA | ACACTCTTTCCCTACACGACGCTCTTCCGATCTCTGTGGTA | CWGTACCACAGAGATCGGAAGAGCGTCGTGTAGGGAAAGAGTGT |
| ApeKI_ G | TGCTTAAT | ACACTCTTTCCCTACACGACGCTCTTCCGATCTTGCTTAAT | CWGATTAAGCAAGATCGGAAGAGCGTCGTGTAGGGAAAGAGTGT |
| ApeKI_ G | AATGACGA | ACACTCTTTCCCTACACGACGCTCTTCCGATCTAATGACGA | CWGTCGTCATTAGATCGGAAGAGCGTCGTGTAGGGAAAGAGTGT |
| ApeKI_ G | AGAACGAT | ACACTCTTTCCCTACACGACGCTCTTCCGATCTAGAACGAT | CWGATCGTTCTAGATCGGAAGAGCGTCGTGTAGGGAAAGAGTGT |
| ApeKI_ G | GTGCGATT | ACACTCTTTCCCTACACGACGCTCTTCCGATCTGTGCGATT | CWGAATCGCACAGATCGGAAGAGCGTCGTGTAGGGAAAGAGTGT |
| ApeKI_ G | CTAATTGA | ACACTCTTTCCCTACACGACGCTCTTCCGATCTCTAATTGA | CWGTCAATTAGAGATCGGAAGAGCGTCGTGTAGGGAAAGAGTGT |
| ApeKI_ G | TAGCAGTA | ACACTCTTTCCCTACACGACGCTCTTCCGATCTTAGCAGTA | CWGTACTGCTAAGATCGGAAGAGCGTCGTGTAGGGAAAGAGTGT |
| ApeKI_ H | AACA | ACACTCTTTCCCTACACGACGCTCTTCCGATCTAACA | CWGTGTTAGATCGGAAGAGCGTCGTGTAGGGAAAGAGTGT |
| ApeKI_ H | CTGT | ACACTCTTTCCCTACACGACGCTCTTCCGATCTCTGT | CWGACAGAGATCGGAAGAGCGTCGTGTAGGGAAAGAGTGT |
| ApeKI_ H | TAAC | ACACTCTTTCCCTACACGACGCTCTTCCGATCTTAAC | CWGGTTAAGATCGGAAGAGCGTCGTGTAGGGAAAGAGTGT |
| ApeKI_ H | CTCGA | ACACTCTTTCCCTACACGACGCTCTTCCGATCTCTCGA | CWGTCGAGAGATCGGAAGAGCGTCGTGTAGGGAAAGAGTGT |
| ApeKI_ H | GACTC | ACACTCTTTCCCTACACGACGCTCTTCCGATCTGACTC | CWGGAGTCAGATCGGAAGAGCGTCGTGTAGGGAAAGAGTGT |
| ApeKI_ H | GCATCC | ACACTCTTTCCCTACACGACGCTCTTCCGATCTGCATCC | CWGGGATGCAGATCGGAAGAGCGTCGTGTAGGGAAAGAGTGT |
| ApeKI_ H | CGTACC | ACACTCTTTCCCTACACGACGCTCTTCCGATCTCGTACC | CWGGGTACGAGATCGGAAGAGCGTCGTGTAGGGAAAGAGTGT |
| ApeKI_ H | ATTCAGT | ACACTCTTTCCCTACACGACGCTCTTCCGATCTATTCAGT | CWGACTGAATAGATCGGAAGAGCGTCGTGTAGGGAAAGAGTGT |
| ApeKI_ H | TATGTAC | ACACTCTTTCCCTACACGACGCTCTTCCGATCTTATGTAC | CWGGTACATAAGATCGGAAGAGCGTCGTGTAGGGAAAGAGTGT |
| ApeKI_ H | AGACAAGT | ACACTCTTTCCCTACACGACGCTCTTCCGATCTAGACAAGT | CWGACTTGTCTAGATCGGAAGAGCGTCGTGTAGGGAAAGAGTGT |
| ApeKI_ H | GGCCGAGT | ACACTCTTTCCCTACACGACGCTCTTCCGATCTGGCCGAGT | CWGACTCGGCCAGATCGGAAGAGCGTCGTGTAGGGAAAGAGTGT |
| ApeKI_ H | AACAAGGT | ACACTCTTTCCCTACACGACGCTCTTCCGATCTAACAAGGT | CWGACCTTGTTAGATCGGAAGAGCGTCGTGTAGGGAAAGAGTGT |
| ApeKI_ H | ACGTC | ACACTCTTTCCCTACACGACGCTCTTCCGATCTACGTC | CWGGACGTAGATCGGAAGAGCGTCGTGTAGGGAAAGAGTGT |
| ApeKI_ H | CGATC | ACACTCTTTCCCTACACGACGCTCTTCCGATCTCGATC | CWGGATCGAGATCGGAAGAGCGTCGTGTAGGGAAAGAGTGT |
| ApeKI_ H | ATGCGT | ACACTCTTTCCCTACACGACGCTCTTCCGATCTATGCGT | CWGACGCATAGATCGGAAGAGCGTCGTGTAGGGAAAGAGTGT |
| ApeKI_ H | TCCAGT | ACACTCTTTCCCTACACGACGCTCTTCCGATCTTCCAGT | CWGACTGGAAGATCGGAAGAGCGTCGTGTAGGGAAAGAGTGT |
| ApeKI_ H | GCTTGA | ACACTCTTTCCCTACACGACGCTCTTCCGATCTGCTTGA | CWGTCAAGCAGATCGGAAGAGCGTCGTGTAGGGAAAGAGTGT |
| ApeKI_ H | TCCGTA | ACACTCTTTCCCTACACGACGCTCTTCCGATCTTCCGTA | CWGTACGGAAGATCGGAAGAGCGTCGTGTAGGGAAAGAGTGT |
| ApeKI_ H | TCGGAT | ACACTCTTTCCCTACACGACGCTCTTCCGATCTTCGGAT | CWGATCCGAAGATCGGAAGAGCGTCGTGTAGGGAAAGAGTGT |
| ApeKI_ H | GTCTTA | ACACTCTTTCCCTACACGACGCTCTTCCGATCTGTCTTA | CWGTAAGACAGATCGGAAGAGCGTCGTGTAGGGAAAGAGTGT |
| ApeKI_ H | CGGAGT | ACACTCTTTCCCTACACGACGCTCTTCCGATCTCGGAGT | CWGACTCCGAGATCGGAAGAGCGTCGTGTAGGGAAAGAGTGT |
| ApeKI_ H | CGTCTA | ACACTCTTTCCCTACACGACGCTCTTCCGATCTCGTCTA | CWGTAGACGAGATCGGAAGAGCGTCGTGTAGGGAAAGAGTGT |
| ApeKI_ H | CACGTT | ACACTCTTTCCCTACACGACGCTCTTCCGATCTCACGTT | CWGAACGTGAGATCGGAAGAGCGTCGTGTAGGGAAAGAGTGT |
| ApeKI_ H | GTTAGC | ACACTCTTTCCCTACACGACGCTCTTCCGATCTGTTAGC | CWGGCTAACAGATCGGAAGAGCGTCGTGTAGGGAAAGAGTGT |
| ApeKI_ H | ATGACGC | ACACTCTTTCCCTACACGACGCTCTTCCGATCTATGACGC | CWGGCGTCATAGATCGGAAGAGCGTCGTGTAGGGAAAGAGTGT |
| ApeKI_ H | TATTGCA | ACACTCTTTCCCTACACGACGCTCTTCCGATCTTATTGCA | CWGTGCAATAAGATCGGAAGAGCGTCGTGTAGGGAAAGAGTGT |
| ApeKI_ H | GGATATC | ACACTCTTTCCCTACACGACGCTCTTCCGATCTGGATATC | CWGGATATCCAGATCGGAAGAGCGTCGTGTAGGGAAAGAGTGT |
| ApeKI_ H | CCGAGCT | ACACTCTTTCCCTACACGACGCTCTTCCGATCTCCGAGCT | CWGAGCTCGGAGATCGGAAGAGCGTCGTGTAGGGAAAGAGTGT |
| ApeKI_ H | TTGATAC | ACACTCTTTCCCTACACGACGCTCTTCCGATCTTTGATAC | CWGGTATCAAAGATCGGAAGAGCGTCGTGTAGGGAAAGAGTGT |
| ApeKI_ H | CACGACT | ACACTCTTTCCCTACACGACGCTCTTCCGATCTCACGACT | CWGAGTCGTGAGATCGGAAGAGCGTCGTGTAGGGAAAGAGTGT |
| ApeKI_ H | ATCTAGC | ACACTCTTTCCCTACACGACGCTCTTCCGATCTATCTAGC | CWGGCTAGATAGATCGGAAGAGCGTCGTGTAGGGAAAGAGTGT |
| ApeKI_ H | TGAATTC | ACACTCTTTCCCTACACGACGCTCTTCCGATCTTGAATTC | CWGGAATTCAAGATCGGAAGAGCGTCGTGTAGGGAAAGAGTGT |
| ApeKI_ H | CGGCTCA | ACACTCTTTCCCTACACGACGCTCTTCCGATCTCGGCTCA | CWGTGAGCCGAGATCGGAAGAGCGTCGTGTAGGGAAAGAGTGT |
| ApeKI_ H | GGTACAC | ACACTCTTTCCCTACACGACGCTCTTCCGATCTGGTACAC | CWGGTGTACCAGATCGGAAGAGCGTCGTGTAGGGAAAGAGTGT |
| ApeKI_ H | AATGTCT | ACACTCTTTCCCTACACGACGCTCTTCCGATCTAATGTCT | CWGAGACATTAGATCGGAAGAGCGTCGTGTAGGGAAAGAGTGT |
| ApeKI_ H | TCATGAC | ACACTCTTTCCCTACACGACGCTCTTCCGATCTTCATGAC | CWGGTCATGAAGATCGGAAGAGCGTCGTGTAGGGAAAGAGTGT |
| ApeKI_ H | TTCGGAC | ACACTCTTTCCCTACACGACGCTCTTCCGATCTTTCGGAC | CWGGTCCGAAAGATCGGAAGAGCGTCGTGTAGGGAAAGAGTGT |
| ApeKI_ H | GCTTAGC | ACACTCTTTCCCTACACGACGCTCTTCCGATCTGCTTAGC | CWGGCTAAGCAGATCGGAAGAGCGTCGTGTAGGGAAAGAGTGT |
| ApeKI_ H | ACTGCCA | ACACTCTTTCCCTACACGACGCTCTTCCGATCTACTGCCA | CWGTGGCAGTAGATCGGAAGAGCGTCGTGTAGGGAAAGAGTGT |
| ApeKI_ H | TAGTACT | ACACTCTTTCCCTACACGACGCTCTTCCGATCTTAGTACT | CWGAGTACTAAGATCGGAAGAGCGTCGTGTAGGGAAAGAGTGT |
| ApeKI_ H | GACACTC | ACACTCTTTCCCTACACGACGCTCTTCCGATCTGACACTC | CWGGAGTGTCAGATCGGAAGAGCGTCGTGTAGGGAAAGAGTGT |
| ApeKI_ H | CGAGTAC | ACACTCTTTCCCTACACGACGCTCTTCCGATCTCGAGTAC | CWGGTACTCGAGATCGGAAGAGCGTCGTGTAGGGAAAGAGTGT |
| ApeKI_ H | ATATGTC | ACACTCTTTCCCTACACGACGCTCTTCCGATCTATATGTC | CWGGACATATAGATCGGAAGAGCGTCGTGTAGGGAAAGAGTGT |
| ApeKI_ H | TGGACCT | ACACTCTTTCCCTACACGACGCTCTTCCGATCTTGGACCT | CWGAGGTCCAAGATCGGAAGAGCGTCGTGTAGGGAAAGAGTGT |
| ApeKI_ H | CATATGC | ACACTCTTTCCCTACACGACGCTCTTCCGATCTCATATGC | CWGGCATATGAGATCGGAAGAGCGTCGTGTAGGGAAAGAGTGT |
| ApeKI_ H | TTCGCCA | ACACTCTTTCCCTACACGACGCTCTTCCGATCTTTCGCCA | CWGTGGCGAAAGATCGGAAGAGCGTCGTGTAGGGAAAGAGTGT |
| ApeKI_ H | AGTTACT | ACACTCTTTCCCTACACGACGCTCTTCCGATCTAGTTACT | CWGAGTAACTAGATCGGAAGAGCGTCGTGTAGGGAAAGAGTGT |
| ApeKI_ H | GTGCCAC | ACACTCTTTCCCTACACGACGCTCTTCCGATCTGTGCCAC | CWGGTGGCACAGATCGGAAGAGCGTCGTGTAGGGAAAGAGTGT |
| ApeKI_ H | ACCGGTC | ACACTCTTTCCCTACACGACGCTCTTCCGATCTACCGGTC | CWGGACCGGTAGATCGGAAGAGCGTCGTGTAGGGAAAGAGTGT |
| ApeKI_ H | CGGTAGC | ACACTCTTTCCCTACACGACGCTCTTCCGATCTCGGTAGC | CWGGCTACCGAGATCGGAAGAGCGTCGTGTAGGGAAAGAGTGT |
| ApeKI_ H | GCAGTTC | ACACTCTTTCCCTACACGACGCTCTTCCGATCTGCAGTTC | CWGGAACTGCAGATCGGAAGAGCGTCGTGTAGGGAAAGAGTGT |
| ApeKI_ H | TATAGTC | ACACTCTTTCCCTACACGACGCTCTTCCGATCTTATAGTC | CWGGACTATAAGATCGGAAGAGCGTCGTGTAGGGAAAGAGTGT |
| ApeKI_ H | ACTCCGC | ACACTCTTTCCCTACACGACGCTCTTCCGATCTACTCCGC | CWGGCGGAGTAGATCGGAAGAGCGTCGTGTAGGGAAAGAGTGT |
| ApeKI_ H | GTATTAC | ACACTCTTTCCCTACACGACGCTCTTCCGATCTGTATTAC | CWGGTAATACAGATCGGAAGAGCGTCGTGTAGGGAAAGAGTGT |
| ApeKI_ H | TGAGCGC | ACACTCTTTCCCTACACGACGCTCTTCCGATCTTGAGCGC | CWGGCGCTCAAGATCGGAAGAGCGTCGTGTAGGGAAAGAGTGT |
| ApeKI_ H | GAGTCGC | ACACTCTTTCCCTACACGACGCTCTTCCGATCTGAGTCGC | CWGGCGACTCAGATCGGAAGAGCGTCGTGTAGGGAAAGAGTGT |
| ApeKI_ H | ATTGATC | ACACTCTTTCCCTACACGACGCTCTTCCGATCTATTGATC | CWGGATCAATAGATCGGAAGAGCGTCGTGTAGGGAAAGAGTGT |
| ApeKI_ H | TCCACGC | ACACTCTTTCCCTACACGACGCTCTTCCGATCTTCCACGC | CWGGCGTGGAAGATCGGAAGAGCGTCGTGTAGGGAAAGAGTGT |
| ApeKI_ H | GTTATTC | ACACTCTTTCCCTACACGACGCTCTTCCGATCTGTTATTC | CWGGAATAACAGATCGGAAGAGCGTCGTGTAGGGAAAGAGTGT |
| ApeKI_ H | ATGCACC | ACACTCTTTCCCTACACGACGCTCTTCCGATCTATGCACC | CWGGGTGCATAGATCGGAAGAGCGTCGTGTAGGGAAAGAGTGT |
| ApeKI_ H | TCCGATAT | ACACTCTTTCCCTACACGACGCTCTTCCGATCTTCCGATAT | CWGATATCGGAAGATCGGAAGAGCGTCGTGTAGGGAAAGAGTGT |
| ApeKI_ H | GCCTCCGA | ACACTCTTTCCCTACACGACGCTCTTCCGATCTGCCTCCGA | CWGTCGGAGGCAGATCGGAAGAGCGTCGTGTAGGGAAAGAGTGT |
| ApeKI_ H | AGTCGTGT | ACACTCTTTCCCTACACGACGCTCTTCCGATCTAGTCGTGT | CWGACACGACTAGATCGGAAGAGCGTCGTGTAGGGAAAGAGTGT |
| ApeKI_ H | CATGTATT | ACACTCTTTCCCTACACGACGCTCTTCCGATCTCATGTATT | CWGAATACATGAGATCGGAAGAGCGTCGTGTAGGGAAAGAGTGT |
| ApeKI_ H | TGAAGCAA | ACACTCTTTCCCTACACGACGCTCTTCCGATCTTGAAGCAA | CWGTTGCTTCAAGATCGGAAGAGCGTCGTGTAGGGAAAGAGTGT |
| ApeKI_ H | GCATTGGT | ACACTCTTTCCCTACACGACGCTCTTCCGATCTGCATTGGT | CWGACCAATGCAGATCGGAAGAGCGTCGTGTAGGGAAAGAGTGT |
| ApeKI_ H | AAGCGATA | ACACTCTTTCCCTACACGACGCTCTTCCGATCTAAGCGATA | CWGTATCGCTTAGATCGGAAGAGCGTCGTGTAGGGAAAGAGTGT |
| ApeKI_ H | GTCAATAT | ACACTCTTTCCCTACACGACGCTCTTCCGATCTGTCAATAT | CWGATATTGACAGATCGGAAGAGCGTCGTGTAGGGAAAGAGTGT |
| ApeKI_ H | CGGCCGTA | ACACTCTTTCCCTACACGACGCTCTTCCGATCTCGGCCGTA | CWGTACGGCCGAGATCGGAAGAGCGTCGTGTAGGGAAAGAGTGT |
| ApeKI_ H | TTAGTCGA | ACACTCTTTCCCTACACGACGCTCTTCCGATCTTTAGTCGA | CWGTCGACTAAAGATCGGAAGAGCGTCGTGTAGGGAAAGAGTGT |
| ApeKI_ H | GACTCAAT | ACACTCTTTCCCTACACGACGCTCTTCCGATCTGACTCAAT | CWGATTGAGTCAGATCGGAAGAGCGTCGTGTAGGGAAAGAGTGT |
| ApeKI_ H | ACTAGGAA | ACACTCTTTCCCTACACGACGCTCTTCCGATCTACTAGGAA | CWGTTCCTAGTAGATCGGAAGAGCGTCGTGTAGGGAAAGAGTGT |
| ApeKI_ H | CGCAACTT | ACACTCTTTCCCTACACGACGCTCTTCCGATCTCGCAACTT | CWGAAGTTGCGAGATCGGAAGAGCGTCGTGTAGGGAAAGAGTGT |
| ApeKI_ H | TCGGTTGA | ACACTCTTTCCCTACACGACGCTCTTCCGATCTTCGGTTGA | CWGTCAACCGAAGATCGGAAGAGCGTCGTGTAGGGAAAGAGTGT |
| ApeKI_ H | AATTCTGT | ACACTCTTTCCCTACACGACGCTCTTCCGATCTAATTCTGT | CWGACAGAATTAGATCGGAAGAGCGTCGTGTAGGGAAAGAGTGT |
| ApeKI_ H | CTAGAGTA | ACACTCTTTCCCTACACGACGCTCTTCCGATCTCTAGAGTA | CWGTACTCTAGAGATCGGAAGAGCGTCGTGTAGGGAAAGAGTGT |
| ApeKI_ H | GCGCGCAT | ACACTCTTTCCCTACACGACGCTCTTCCGATCTGCGCGCAT | CWGATGCGCGCAGATCGGAAGAGCGTCGTGTAGGGAAAGAGTGT |
| ApeKI_ H | CGATTAGA | ACACTCTTTCCCTACACGACGCTCTTCCGATCTCGATTAGA | CWGTCTAATCGAGATCGGAAGAGCGTCGTGTAGGGAAAGAGTGT |
| ApeKI_ H | ATTCAGTT | ACACTCTTTCCCTACACGACGCTCTTCCGATCTATTCAGTT | CWGAACTGAATAGATCGGAAGAGCGTCGTGTAGGGAAAGAGTGT |
| ApeKI_ H | TTGACAAT | ACACTCTTTCCCTACACGACGCTCTTCCGATCTTTGACAAT | CWGATTGTCAAAGATCGGAAGAGCGTCGTGTAGGGAAAGAGTGT |
| ApeKI_ H | TACGTTAA | ACACTCTTTCCCTACACGACGCTCTTCCGATCTTACGTTAA | CWGTTAACGTAAGATCGGAAGAGCGTCGTGTAGGGAAAGAGTGT |
| ApeKI_ H | AGATACGA | ACACTCTTTCCCTACACGACGCTCTTCCGATCTAGATACGA | CWGTCGTATCTAGATCGGAAGAGCGTCGTGTAGGGAAAGAGTGT |
| ApeKI_ H | GCTGGATT | ACACTCTTTCCCTACACGACGCTCTTCCGATCTGCTGGATT | CWGAATCCAGCAGATCGGAAGAGCGTCGTGTAGGGAAAGAGTGT |
| ApeKI_ H | GACACCTT | ACACTCTTTCCCTACACGACGCTCTTCCGATCTGACACCTT | CWGAAGGTGTCAGATCGGAAGAGCGTCGTGTAGGGAAAGAGTGT |
| ApeKI_ H | CCGCCTAA | ACACTCTTTCCCTACACGACGCTCTTCCGATCTCCGCCTAA | CWGTTAGGCGGAGATCGGAAGAGCGTCGTGTAGGGAAAGAGTGT |
| ApeKI_ H | TTCTTGGA | ACACTCTTTCCCTACACGACGCTCTTCCGATCTTTCTTGGA | CWGTCCAAGAAAGATCGGAAGAGCGTCGTGTAGGGAAAGAGTGT |
| ApeKI_ H | CAACGCTT | ACACTCTTTCCCTACACGACGCTCTTCCGATCTCAACGCTT | CWGAAGCGTTGAGATCGGAAGAGCGTCGTGTAGGGAAAGAGTGT |
| ApeKI_ H | AGCAGATA | ACACTCTTTCCCTACACGACGCTCTTCCGATCTAGCAGATA | CWGTATCTGCTAGATCGGAAGAGCGTCGTGTAGGGAAAGAGTGT |
| ApeKI_ H | TCTTAGGT | ACACTCTTTCCCTACACGACGCTCTTCCGATCTTCTTAGGT | CWGACCTAAGAAGATCGGAAGAGCGTCGTGTAGGGAAAGAGTGT |
| ApeKI_ H | GTGGCTAT | ACACTCTTTCCCTACACGACGCTCTTCCGATCTGTGGCTAT | CWGATAGCCACAGATCGGAAGAGCGTCGTGTAGGGAAAGAGTGT |
| ApeKI_ H | AGACTGAA | ACACTCTTTCCCTACACGACGCTCTTCCGATCTAGACTGAA | CWGTTCAGTCTAGATCGGAAGAGCGTCGTGTAGGGAAAGAGTGT |
| ApeKI_ H | CATAATGA | ACACTCTTTCCCTACACGACGCTCTTCCGATCTCATAATGA | CWGTCATTATGAGATCGGAAGAGCGTCGTGTAGGGAAAGAGTGT |
| ApeKI_ H | GTGTCAGT | ACACTCTTTCCCTACACGACGCTCTTCCGATCTGTGTCAGT | CWGACTGACACAGATCGGAAGAGCGTCGTGTAGGGAAAGAGTGT |
| ApeKI_ H | AGTGGCTT | ACACTCTTTCCCTACACGACGCTCTTCCGATCTAGTGGCTT | CWGAAGCCACTAGATCGGAAGAGCGTCGTGTAGGGAAAGAGTGT |
| ApeKI_ H | CCACTGCA | ACACTCTTTCCCTACACGACGCTCTTCCGATCTCCACTGCA | CWGTGCAGTGGAGATCGGAAGAGCGTCGTGTAGGGAAAGAGTGT |
| ApeKI_ H | TACAGGAT | ACACTCTTTCCCTACACGACGCTCTTCCGATCTTACAGGAT | CWGATCCTGTAAGATCGGAAGAGCGTCGTGTAGGGAAAGAGTGT |
| ApeKI_Common | None | CWGAGATCGGAAGAGCGGTTCAGCAGGAATGCCGAG | CTCGGCATTCCTGCTGAACCGCTCTTCCGATCT |
