# Supplementary material for: Heterozygous Mapping Strategy (HetMappS) for High Resolution Genotyping-By-Sequencing Markers: A Case Study in Grapevine
Source: PLoS One. 2015 Aug 5;10(8):e0134880. doi: 10.1371/journal.pone.0134880 (PMC4526651; doi:10.1371/journal.pone.0134880)
Supplement: S2 Table — (DOCX) [file pone.0134880.s020.docx]

**S3 Table: TASSEL-GBS plugins (Glaubitz et al., 2014) used in HetMappS (Hyma et al.)**

| **Tag generation Plugin** | **Option** | value | **Description** |
| --- | --- | --- | --- |
| FastqToTagCountPlugin | -s | 300000000 | Maximum number of good reads per lane. Default: 200000000 |
| FastqToTagCountPlugin | -c | 1 | Minimum number of times a tag must be present to be output. Default: 1 |
| MergeMultipleTagCountPlugin | -c | 5 | Minimum number of times a tag must be present to be output. Default: 1 |
| **Tags by taxa Plugin** | **Option** | value | **Description** |
| FastqToTBTPlugin | -c | 5 | Minimum taxa count within a fastq file for a tag to be output. Default: 1 |
| FastqToTBTPlugin | -s | 300,000,000 | Max good reads per lane. (Optional. Default is 200,000,000). |
| FastqToTBTPlugin | -y | -y | Output in TBTByte format (counts from 0-127) instead of TBTBit (0 or 1). |
| MergeTagsByTaxaFilesPlugin | -s | 300,000,000 | Maximum number of tags the TBT can hold while merging (default: 200,000,000). Reduce this only if you run out of memory (omit the commas). |
| MergeTagsByTaxaFilesPlugin | -x | -x | Merges tag counts of taxa with identical names if set to -x. Not performed by default |
| **SNP calling Plugin** | **Option** | value | **Description** |
| tbt2vcfPlugin | -ak | 4 | Maximum number of alleles that are kept for each marker across the population; default: 3 |
| tbt2vcfPlugin | -mnMAF | 0 | Minimum minor allele frequency (default: 0.0) |
| tbt2vcfPlugin | -mnLCov | 0 | Minimum locus coverage (proportion of Taxa with a genotype) (default: 0.0) |
| MergeDuplicateSNP_vcf_Plugin | -ak | 4 | Maximum number of alleles that are kept for each marker across the population; default: 3 |
