# Supplementary material for: Heterozygous Mapping Strategy (HetMappS) for High Resolution Genotyping-By-Sequencing Markers: A Case Study in Grapevine
Source: PLoS One. 2015 Aug 5;10(8):e0134880. doi: 10.1371/journal.pone.0134880 (PMC4526651; doi:10.1371/journal.pone.0134880)
Supplement: S4 Table — (DOCX) [file pone.0134880.s022.docx]

S4 Table. Selected LOD score thresholds from the 'FilterByLODSlidingDroponemarker' function, and number of markers removed during the auto-filter stage of map curation.

|  |  | Synteny Pipeline | | *de novo* Pipeline | |
| --- | --- | --- | --- | --- | --- |
| F1 family | LOD | Autodropped markers (female) | Autodropped markers (male) | Autodropped markers (female) | Autodropped markers (male) |
| *V. rupestris* B38 x ‘Chardonnay' | -12 | 21 (1.7%) | 40 (2.9%) | 87 (5.6%) | 86 (5.2%) |
| *V. rupestris* B38 x ‘Horizon’ | -30 | 46 (2.2%) | 73 (1.9%) | 127 (4.9%) | 182 (4.1%) |
| ‘Horizon’ x Illinois 547-1 | -50 | 69 (1.7%) | 97 (1.8%) | 149 (3.1%) | 170 (2.8%) |
| ‘Chardonnay’ x *V. cinerea* B9 | -20 | 43 (1.8%) | 45 (2.2%) | 96 (3.5%) | 96 (3.8%) |
| ‘Horizon’ x *V. cinerea* B9 | -25 | 117 (4.0%) | 43 (2.6%) | 164 (4.6%) | 110 (4.8%) |

The selected LOD score was applied in both the synteny and *de novo* pipelines. Percentages of markers are relative to the number of ordered markers prior to applying the ‘FilterByLODSlidingDroponemarker’ function.
