# Supplementary material for: Heterozygous Mapping Strategy (HetMappS) for High Resolution Genotyping-By-Sequencing Markers: A Case Study in Grapevine
Source: PLoS One. 2015 Aug 5;10(8):e0134880. doi: 10.1371/journal.pone.0134880 (PMC4526651; doi:10.1371/journal.pone.0134880)
Supplement: S5 Table — (DOCX) [file pone.0134880.s023.docx]

S5 Table. Summary of GBS sequence tags and alignment results for four F_1_ families.

| F1 Family | # Tags | uniquely aligned tags | multiply aligned tags | unaligned tags |
| --- | --- | --- | --- | --- |
| *V. rupestris* B38 x ‘Horizon’ | 1,116,043 | 743,162 (67%) | 126,688 (11%) | 246,193 (22%) |
| ‘Horizon’ x  Illinois 547-1 | 1,219,257 | 806,213 (66%) | 141,359 (12%) | 271,685 (22%) |
| ‘Chardonnay’ x  *V. cinerea* B9 | 852,885 | 616,697 (72%) | 102,661 (12%) | 133,527 (16%) |
| ‘Horizon’ x  *V. cinerea* B9 | 862,415 | 622,904 (72%) | 103,633 (12%) | 135,878 (16%) |
