# Supplementary material for: Heterozygous Mapping Strategy (HetMappS) for High Resolution Genotyping-By-Sequencing Markers: A Case Study in Grapevine
Source: PLoS One. 2015 Aug 5;10(8):e0134880. doi: 10.1371/journal.pone.0134880 (PMC4526651; doi:10.1371/journal.pone.0134880)
Supplement: S6 Table — (DOCX) [file pone.0134880.s024.docx]

S6 Table. Summary of GBS datasets before and after family level quality control.

| F1 Family | Progeny (unfiltered) | Progeny (filtered) | SNPs  (unfiltered) | SNPs  (filtered) | Progenitors genotyped |
| --- | --- | --- | --- | --- | --- |
| *V. rupestris* B38 x ‘Horizon’ | 215 | 214 | 337,459 | 337,365 | 2 |
| ‘Horizon’ x  Illinois 547-1 | 366 | 358 | 450,937 | 449,840 | 4 |
| ‘Chardonnay’ x  *V. cinerea* B9 | 148 | 148 | 300,773 | 300,773 | 2 |
| ‘Horizon’ x  *V. cinerea* B9 | 162 | 156 | 333,366 | 331,356 | 2 |
