# Supplementary material for: Heterozygous Mapping Strategy (HetMappS) for High Resolution Genotyping-By-Sequencing Markers: A Case Study in Grapevine
Source: PLoS One. 2015 Aug 5;10(8):e0134880. doi: 10.1371/journal.pone.0134880 (PMC4526651; doi:10.1371/journal.pone.0134880)
Supplement: S7 Table — (DOCX) [file pone.0134880.s025.docx]

| HetMappS SNP input | | VitisGen F_1_ families mean | *V. rupestris* B38 x ‘Chardonnay’ |
| --- | --- | --- | --- |
|  |  | 354,834 | 116,466 |
| Number (percentage) of input SNPs lost at key steps | **geno,**  **<5% frequency** | 119,667 (34%) | 30,241  (26%) |
|  | **monomorphic** | 129,629 (36%) | 23,730  (20%) |
|  | **segregation ratio** | 47,181  (14%) | 29,619  (25%) |
|  | **low geno after masking** | 43,146  (12%) | 14,396  (12%) |
|  | **high error rate** | 395  (0.1%) | 1,213  (1.0%) |

S7 Table. Pseudotestcross marker identification in a pre-VitisGen sample.
