# Supplementary material for: Heterozygous Mapping Strategy (HetMappS) for High Resolution Genotyping-By-Sequencing Markers: A Case Study in Grapevine
Source: PLoS One. 2015 Aug 5;10(8):e0134880. doi: 10.1371/journal.pone.0134880 (PMC4526651; doi:10.1371/journal.pone.0134880)
Supplement: S8 Table — (DOCX) [file pone.0134880.s026.docx]

|  | VitisGen F_1_ families mean | *V. rupestris* B38 x ‘Chardonnay’ |
| --- | --- | --- |
| Pt input | 14,817 | 17,267 |
| Markers on random chromosomes | 898  (6%) | 1014  (6%) |
| In linkage with another chromosome (“disagree”) | 935  (6%) | 2514  (15%) |
| In linkage with multiple chromosomes (“Unresolved”) | 1,240 (9%) | 5,233  (30%) |
| In linkage with aligned chromosome (“Agree”) | 11,744 (79%) | 8,506  (49%) |

S8 Table. Correlation based chromosome assignment in the synteny pipeline, for a pre-VitisGen sample.
