# Supplementary material for: Heterozygous Mapping Strategy (HetMappS) for High Resolution Genotyping-By-Sequencing Markers: A Case Study in Grapevine
Source: PLoS One. 2015 Aug 5;10(8):e0134880. doi: 10.1371/journal.pone.0134880 (PMC4526651; doi:10.1371/journal.pone.0134880)
Supplement: S9 Table — (DOCX) [file pone.0134880.s027.docx]

S9 Table. Linkage group (LG) and phase assignment for the pre-VitisGen *Vitis rupestris* B38 x ‘Chardonnay’ F_1_ family. Analyzed with the synteny and the *de* novo pipelines.

| F_1_ family  (size) | # PtM in chromosomal groups | Cut Height | # LGs | Split LGs  (Parental map) | # PtM in phased LGs | # ordered PtM after filtering |
| --- | --- | --- | --- | --- | --- | --- |
| Synteny pipeline | 8,506  (49.3%) | 0.825 | 47 | 1,2,7,11,18  (*V. rupestris* B38)  5,7,14,18 (‘Chardonnay’) | 8,356  (48.4%) | 2,669 (15.5%) |
| *De novo* pipeline | n.a. | 0.875 | 66 of 71 | 1,2,3,4,7,8,9,  12,13,15,18,19  (*V. rupestris* B38)  4,8,10,15,17  (‘Chardonnay’) | 10,799 (62.5%) | 3,365 (19.5%) |

Percentages below SNP number are relative to the number of markers entering each pipeline.
