# Supplementary material for: Heterozygous Mapping Strategy (HetMappS) for High Resolution Genotyping-By-Sequencing Markers: A Case Study in Grapevine
Source: PLoS One. 2015 Aug 5;10(8):e0134880. doi: 10.1371/journal.pone.0134880 (PMC4526651; doi:10.1371/journal.pone.0134880)
Supplement: S10 Table — (DOCX) [file pone.0134880.s028.docx]

S10 Table. Linkage groups with suspicious orders from the HetMappS pipelines, detected at genetic map curation.

|  | *de novo* | | synteny | |
| --- | --- | --- | --- | --- |
|  | female | male | female | male |
| *V. rupestris* B38 x 'Chardonnay' | 5 | 13,14,16 | none | 8,14 |
| *V. rupestris* B38 x 'Horizon' | 12,13 | 12 | 1 | none |
| ‘Horizon' x Illinois 547-1 | 12,19 | 7,12,19 | 16 | 7 |
| 'Chardonnay' x *V. cinerea* B9 | none | 13 | none | 16,17 |
| 'Horizon' x *V. cinerea* B9 | 5,8,12,13,14,16 | 2,9 | none | none |
